# Identification of proteins interacting with the N-terminal half of endoribonuclease RNase E in *Escherichia coli*

**DOI:** 10.1080/15476286.2026.2678028

**Published:** 2026-05-21

**Authors:** Yvonne Göpel, Svetlana Durica-Mitic, Solomiia Boyko, Przemyslaw Dudys, Fabian Amman, Karin Schnetz, Boris Görke

**Affiliations:** a Max Perutz Labs, Vienna BioCenter, Vienna, Austria; b University of Vienna, Vienna, Austria; c Vienna BioCenter PhD Program, A Doctoral School of the University of Vienna and the Medical University of Vienna, Vienna, Austria; d Department of Physiology and Biophysics, Case Western Reserve University, Cleveland, OH, USA; e Center for Anatomy and Cell Biology, Medical University of Vienna, Vienna, Austria; f Institute of Theoretical Biochemistry, University of Vienna, Vienna, Austria; g Ludwig Boltzmann Institute for Science Outreach and Pandemic Preparedness at the Medical University of Vienna, Vienna, Austria; h Institute for Genetics, University of Cologne, Cologne, Germany

**Keywords:** RNase E, RapZ, CsrD, RNA decay, bacterial two-hybrid screen, trans-membrane domain, YegJ

## Abstract

In *E. coli* endoribonuclease RNase E (Rne) plays a central role in RNA processing and decay. Rne consists of an N-terminal catalytic domain (NTD) and a C-terminal scaffolding domain, separated by a membrane targeting sequence (MTS). While the scaffolding domain represents a hub for interaction, only few proteins are known to bind the Rne-NTD. One example is protein RapZ, which forms a transient complex with the Rne-NTD to target a small RNA to decay. Here, we screened a genomic bacterial two-hybrid library for proteins binding to the Rne N-terminal half including the MTS. We identified fragments of 43 proteins, of which 15 were validated to bind Rne as full-length proteins. We distinguished three groups: Group I likely uses a membrane domain to bind a segment in Rne that contains the MTS. Group II proteins are cytoplasmic and bind the Rne-NTD. Group III proteins bind the Rne-NTD and additionally the MTS region, indicating two distinct interaction surfaces. RNA-seq revealed that overproduction of three group II proteins including YegJ generates similar effects on the transcriptome. Genes carrying Rne cleavage sites were enriched among downregulated genes but depleted from upregulated genes, suggesting altered RNA decay. Interestingly, strong overexpression of *yegJ* destabilizes rRNA and arrests growth. Apparently, YegJ damages the membrane, granting periplasmic RNase I and presumably also Rne access to cytoplasmic rRNA. We present a catalogue of proteins likely binding the Rne N-terminal half, which may provide a valuable resource for further investigation of these interactions and their roles.

## Introduction

**Figure 1.**
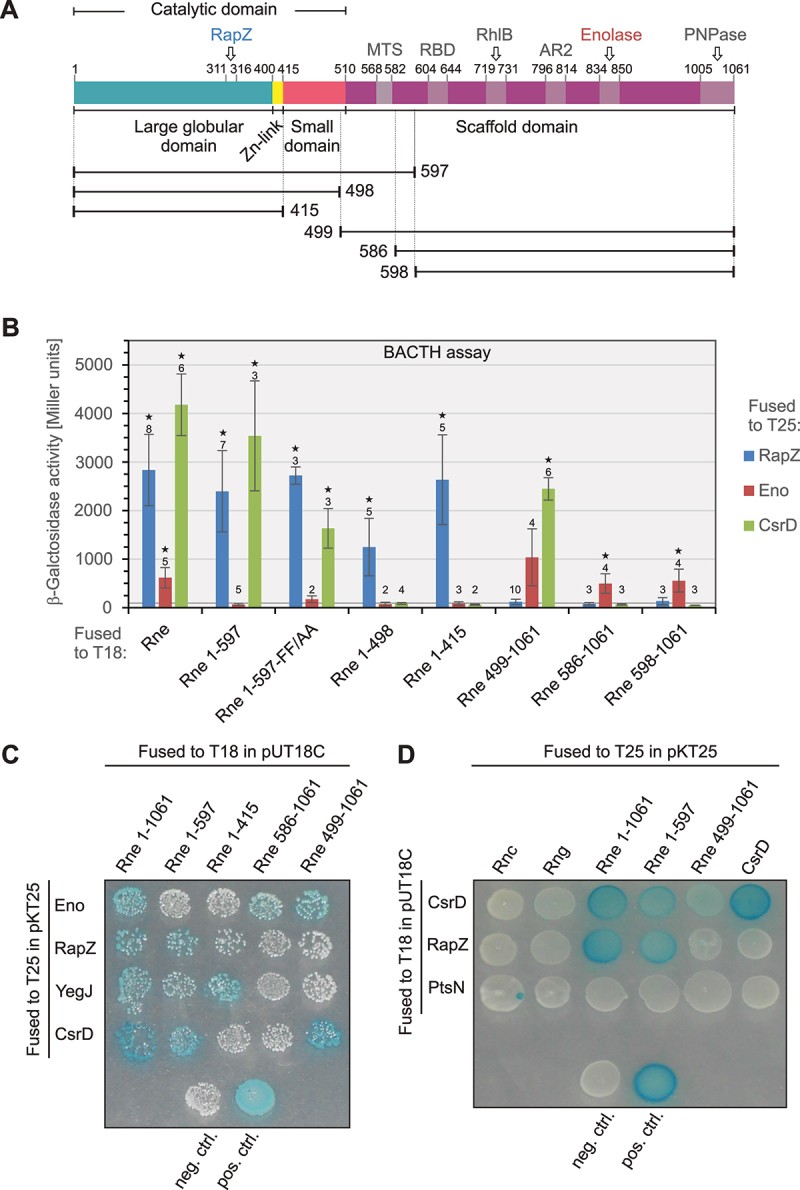
BACTH assays testing interaction of RapZ, enolase and CsrD with various truncated Rne constructs. (A) Schematic representation of the domain organization of Rne depicting location of protein, membrane (MTS) and RNA binding sites (RBD, AR2) as mapped in previous studies [2–4]. The length of the Rne truncations used in this study is indicated by horizontal lines. (B) Quantitative BACTH assays addressing interaction of T25-RapZ (plasmid pBGG348, blue columns), T25-enolase (plasmid pYG95, red columns) and T25-CsrD (plasmid pYG115, green columns) with various Rne variants that were fused to the CyaA T18 fragment. The T18-Rne variants were encoded on the following plasmids: pYG99 (T18-Rne_FL_), pYG97 (T18-Rne_1–597_), pYG195 (T18-Rne_1–597 FF/AA_), pYG168 (T18-Rne_1–498_), pYG144 (T18-Rne_1–415_), pYG98 (T18-Rne_499–1061_), pYG163 (Rne_586–1061_) and pYG162 (T18-Rne_598–1061_). Corresponding double transformants of strain BTH101 were grown for ~16 h at 28°C in LB containing 1 mM IPTG and subsequently the β-galactosidase activities were determined, which are presented as mean ± standard deviation. Replicate numbers (*n*) are indicated. The measurements were analysed by paired two-tailed t-test and activities significantly higher than the negative control as provided by the empty vectors pKT25/pUT18C are labelled with asterisks (*p* < 0.05). The activity produced by the negative control is indicated by a dotted horizontal line. (C) BACTH spotting assays addressing interaction of T25-enolase (pYG95), T25-RapZ (pBGG348), T25-YegJ (pYG141) and T25-CsrD (pYG115) with various truncated Rne variants fused to the CyaA-T18 domain. The plasmids encoding the various T18-Rne variants are described under (B). (D) BACTH spotting assays testing interaction of T18-CsrD (pYG116) with T25-RNase III (Rnc; pYG121), T25-RNase G (Rng; pYG123), T25-CsrD and the following Rne variants, which were fused to T25: T25-Rne_1–1061_ (pYG100), T25-Rne_1–597_ (pYG101) and T25-Rne_499–1061_ (pYG102). T18-RapZ (pBGG349) and T18-PtsN (pYG46) were included as additional positive and negative controls, respectively.

RNase E (Rne) is an essential endoribonuclease that plays a crucial role in RNA processing and degradation, acting as a central regulator of gene expression in *E. coli* and many other bacteria [1,2]. Its functions include the maturation and turnover of stable rRNA and tRNA, and the rapid degradation of mRNA. The Rne protomer consists of a globular N-terminal catalytic domain (residues 1–529) harbouring the endonuclease active site and a largely disordered C-terminal domain (CTD; residues 530–1061) that provides microdomains for binding additional proteins and RNA, and an MTS that anchors the enzyme to the inner membrane (IM; Figure 1(A), top). The helicase RhlB, the 3’→5’ exoribonuclease PNPase and the glycolytic enzyme enolase (eno) bind to distinct sites in the Rne-CTD, collectively forming the degradosome complex, which ensures efficient and coordinated RNA processing and decay. Rne cleaves RNA within single-stranded AU-rich regions with the sequence motif RN↓WU (with R as G/A, W as A/U and ↓ indicating the cleavage site [5]). A recent transcriptome-wide study mapped ~7500 Rne cleavage sites in *E. coli*, of which 64% reside in mRNAs [6]. Thus, at least 25% of ~ 4300 orfs that are transcribed under standard growth conditions are cleaved by Rne.

The Rne-NTD forms a homotetramer and is sufficient for viability. It folds into a large domain and a small domain, both separated by a Zn^2+^ link that contributes to formation of the principal dimer (Figure 1(A) [7,8]). Intersubunit contacts between small domain dimers contribute to assembly of the tetramer. The large domain harbours the catalytic site and the 5’ monophosphate sensing pocket [9]. Interaction of this pocket with the 5’ terminal monophosphate group in RNAs boosts Rne to cleave the transcript at internal sites [10]. Cleavage releases the 3’ cleavage product with a 5’ monophosphate, accelerating further decay. The decapping enzyme RppH converts a group of transcripts that are initially 5’ triphosphorylated to their 5’ monophosphorylated forms, thereby initiating their decay [11,12]. However, other transcripts are recognized by Rne by different means, collectively referred to as the ‘direct entry’ mode of recognition [13,14]. A groove in the Rne-NTD formed by the surface of the small domain and a subdomain within the large domain allows Rne to bind structured RNAs [15,16]. The latter interaction may stimulate Rne allosterically to cleave within adjacent single-stranded regions, independent of the RNA 5’ phosphorylation state [17]. This way, Rne may cleave upstream of transcriptional terminators and initiate rapid 3’→5’ RNA decay by exoribonucleases such as PNPase.

Rne is highly compartmentalized in the bacterial cell [18]. With the help of the MTS, which forms an amphiphatic α-helix, degradosomes are tethered to the IM where they form dynamic short-lived clusters in which RNA degradation occurs [19–21]. A recent study suggests that 93% of all Rne molecules localize at the membrane [22]. This sequestration may protect nascent cytoplasmic RNAs, including rRNAs, from premature cleavage [23]. mRNAs encoding membrane proteins preferentially localize at the membrane due to co-translational insertion of signal peptides and are therefore more prone to degradation by Rne than mRNAs that encode soluble or outer membrane proteins and localize in the cytoplasm [20,23]. In agreement, removal of the MTS in Rne results in slower growth, likely caused by granting Rne access to rRNA precursors in the nucleoid interfering with proper ribosome assembly [24]. Interestingly, certain types of stress including nitrogen starvation, transition to anaerobic growth or arrest of translation elongation may release Rne from the membrane but the underlying mechanisms are unknown [18].

Activity of Rne is subject to extensive regulation, providing an additional level of gene regulation at the post-transcriptional level. Small regulatory RNAs (sRNAs) are known to modulate access of Rne to target RNAs, either inhibiting or stimulating their degradation [25–27]. Additionally, proteins are known to regulate the activity of Rne. Well-known examples include RraA and RraB, which bind to the Rne scaffolding domain thereby inhibiting Rne activity [28–30]. In contrast, only few proteins are known to bind to the N-terminal half of Rne (1–598), which contains the catalytic domain and the MTS. One example is the phage T4 encoded protein Srd, which binds Rne to stimulate degradation of host RNAs [31]. A so far unique mechanism to achieve controlled turnover of a specific transcript by Rne involves the adapter protein RapZ in *E. coli*. RapZ governs stability of the sRNA GlmZ, which in turn controls expression of glucosamine-6-phosphate (GlcN6P) synthase GlmS, the key enzyme initiating cell envelope synthesis [32,33]. GlmZ activates *glmS* expression by base-pairing. RapZ binds and presents GlmZ to programmed decay by Rne, when GlcN6P – the key metabolite initiating envelope biogenesis – is replete and *de novo* synthesis is not required. RapZ forms a tetramer (like the Rne-NTD) and presents GlmZ in a manner that aligns its single-stranded region comprising the cleavage site into the active centre of Rne [3]. The ternary RapZ:GlmZ:Rne complex is stabilized predominantly through mutual binding of the Rne-NTD and RapZ to GlmZ, but direct protein-protein interactions involving the Rne segment 311–316 also contribute (Figure 1(A) [3,34];). An ‘Rne adapter’ function reminiscent of RapZ may also apply to protein CsrD, which controls turnover of sRNAs CsrB/CsrC by Rne [35]. CsrB and CsrC act as sponges and sequester the global RNA-binding protein CsrA, thereby inhibiting its binding to target RNAs. Protection of the sRNAs CsrB/CsrC conferred by binding of CsrA is counteracted by CsrD [36]. CsrD is membrane-associated and attachment of Rne to the cytoplasmic membrane is necessary for the CsrD-mediated degradation of the sRNAs [37]. Whether CsrD also interacts with Rne is unknown.

So far, RapZ and CsrD are the only proteins known to target specific transcripts to programmed decay by Rne. Considering the efficiency of this mechanism allowing cells to quickly degrade or stabilize specific RNAs as required, the existence of yet unknown proteins acting in a similar manner was hypothesized [2]. Such proteins might have escaped previous Rne pull-down approaches [38,39], because they are not or only weakly expressed under standard growth conditions. To overcome these limitations, we used the bacterial two-hybrid system based on the reconstitution of adenylate cyclase in *E. coli* (BACTH [40];) to screen a library of *E. coli* protein fragments for interaction with the Rne-NTD or its paralog Rng. The screen retrieved fragments of 43 proteins, most of which localize to the cytoplasmic membrane. Of these, 15 were confirmed to interact with Rne in BACTH when assessed in their full-length sequences. Using various Rne truncations, we show that these proteins split into three groups regarding the contacted domains. One group includes four proteins interacting exclusively with the Rne-NTD, similar to RapZ, and pull-down approaches confirmed interaction for three of them. RNA-seq analysis of strains overproducing these proteins revealed regulation of mRNAs carrying Rne cleavage sites, suggesting that these proteins affect Rne activity globally when overproduced. One interesting candidate is the so far uncharacterized protein YegJ, which binds the Rne catalytic domain and leads to membrane leakiness and rRNA destabilization accompanied by a growth arrest when overproduced. Taken together, we identify multiple candidate proteins likely binding the N-terminal half of Rne, which provides a groundwork for their future investigation.

## Materials and methods

### Media, growth conditions, strains and plasmids

LB Lennox was used for the routine growth of *E. coli*. Antibiotics and supplements were added at the following concentrations: ampicillin (100 μg/ml), chloramphenicol (15 μg/ml), kanamycin (30 μg/ml), IPTG (1 mM), 5-bromo-4-chloro-3-indolyl-β-D-galactopyranoside (X-Gal, 40 μg/ml). SOB medium contains 20 g bacto tryptone, 5 g bacto yeast extract, 0.5 g NaCl, 1.25 ml 2 M KCl per litre. The pH was adjusted to 7.0 using NaOH. 10 ml 1 M MgCl_2_ was added after autoclavation. To obtain SOC medium, 19.8 ml 20% glucose was added to 1 l SOB medium. M63 maltose minimal medium contained per 1 l: 13.6 g KH_2_PO_4_, 2 g (NH_4_)_2_SO_4_, 0.5 mg FeSO_4_ × 7 H_2_O, 0.5 mM MgSO_4_, 1 μg/ml thiamine, 0.4% (w/v) maltose. The pH was adjusted to pH 7.0 using KOH. To solidify the medium, 15 g/l bacto agar was used. To prepare 1 l M63 maltose minimal medium plates, 15 g bacto agar in 500 ml H_2_O was autoclaved. Subsequently, 200 ml 5× concentrated M63 salts (68 g KH_2_PO_4_, 10 g (NH_4_)_2_SO_4_, 2.5 mg FeSO_4_ × 7 H_2_O per litre, pH 7.0, autoclaved), 0.5 ml MgSO_4_ (1 mg/ml), 1 ml thiamine (1 mg/ml), 20 ml 20% (w/v) maltose and the required antibiotics and supplements were added and filled up with sterile dH_2_O to the final volume.

*E*. *coli* strains and plasmids are listed in Table 1. DNA oligonucleotides are described in Suppl. Table S1. The *Δlon:kan* and *rne598-FLAG-cat* alleles were moved between strains by general transduction using phage T4GT7 [51]. The replacement of the *lac* promoter by the constitutive *P*_*16*_ promoter [52] in strain T3276 was constructed by λRed recombineering [48] using a PCR fragment that was generated with primers OB604/OB605 and plasmid pKES262 as template. Construction of recombinant plasmids followed standard procedures [53]. Details on their construction including information on used vector backbones, restriction endonucleases and primers for generation of inserts by PCR are provided in Suppl. Table S2. The mutagenesis primer BG1308 was used to mutate the Phe (TTC) codons 574 and 575 in *rne*_*1–597*_ to Ala (GCC) codons following the combined chain reaction protocol [54], resulting in plasmid pYG195 (Suppl. Table S2).

**Table 1.** Strains and plasmids used in this study.

Name	Relevant structure or genotype	Reference/construction
Strains		
BL21(DE3)	*F*^−^ *ompT lon gal dcm hsdS*_*B*_*(r*_*B*_^−^ *m*_*B*_^−^*) λ(DE3 [lacI lacUV5-T7 gene 1 ind1 sam7 nin5])*	Laboratory collection
BTH101	*F*^−^, *cya-99, araD139, galE15, galK16, rpsL1 (Str*^*R*^*), hsdR2, mcrA1, mcrB1, relA1*	[41]
BW25113	F^−^, *Δ(araD-araB)567, ΔlacZ4787(::rrnB-3), λ-, rph-1, Δ(rhaD-rhaB)568, hsdR514*	[42]
DH5α	*ϕ 80d lacZΔM15, recA1, endA1, gyrA96, thi-1, hsdR17 (r*_*K*_^−^, *m*_*K*_^*+*^*), supE44, relA1, deoR, Δ(lacZYA-argF) U169*	Laboratory stock
JW0429-1	F^−^, *Δ(araD-araB)567, ΔlacZ4787(::rrnB-3), Δlon-725:kan, λ-, rph-1, Δ(rhaD-rhaB)568, hsdR514*	[42]
JW0603-2	F^−^, *Δ(araD-araB)567, ΔlacZ4787(::rrnB-3), Δrna-749:kan, λ-, rph-1, Δ(rhaD-rhaB)568, hsdR514*	[42]
S4197	MG1655 *rph*^*+*^ *ilvG*^*+*^ *ΔlacZ*	[43]
T407	MG1655 *rph*^*+*^ *ΔlacY*_*FRT*_	[44]
T3276	MG1655 *rph*^*+*^ *Δ(lacI-P*_*lac*_*):kan-P*_*16*_ *lacZ ΔlacY*	PCR OB604+OB605 of pKES262→T407; this work
TM529	W3110 *mlc rne598-FLAG*-cat	[45]
W3110	*F*^−^ *λ*^−^ IN(*rrnD-rrnE*)1	[46]
Z899	S4197 *rne*598-*FLAG-cat*	T4GT7 (TM529)→S4197; this work
Z909	S4197 *rne*598-*FLAG-cat Δlon:kan*	T4GT7 (JW0429-1)→Z899; this work
Plasmids		
pBGG164	*strep-rapZ* under *P*_*tac*_ control, *lacI*^*q*^, *bla*, ori ColEI	[47]
pBGG217	*strep-ptsN* under *P*_*tac*_ control, *lacI*^*q*^, *bla*, ori ColEI	[47]
pBGG237	*strep*-tag under *P*_*tac*_ control, *lacI*^*q*^, *bla*, ori ColEI	[47]
pBGG348	encodes T25-RapZ fusion in pKT25	[32]
pBGG349	encodes T18-RapZ fusion in pUT18C	[32]
pKD13	*FRT-kan-FRT, bla, ori-R6Kγ*	[48]
pKES262	*P*_*16*_ promoter fragment in pKD13, *bla*, *ori-R6Kγ*	this work
pKT25	*P*_*lac*_::*cyaA* [*1–224*] (T25), MCS, *neo*, *ori* p15A	[41]
pKT25-zip	encodes T25-GCN4 leucine zipper fusion in pKT25	[41]
pRne529N	*His*_*6*_*-rne*_*NTD*_ (aa 1–529) in pET16b, *bla*, *ori* ColEI	[49]
pSD2	encodes T25-RNase E (aa 1–762) fusion in pKT25	[34]
pSD3	encodes T25-RNase E (aa 1–415) fusion in pKT25	[34]
pUT18C	*P*_*lac*_::*cyaA* [*225–399*] (T18), MCS, *bla*, *ori* ColEI	[41]
pUT18C-zip	encodes T18-GCN4 leucine zipper fusion in pUT18C	[41]
pYG45	encodes T18-Eno fusion in pUT18C	[32]
pYG46	encodes T18-PtsN fusion in pUT18C	[32]
pYG95	encodes T25-Eno fusion in pKT25	[32]
pYG97	encodes T18-RNase E (aa 1–597) fusion in pUT18C	[32]
pYG98	encodes T18-RNase E (aa 499–1061) fusion in pUT18C	[32]
pYG99	encodes T18-RNase E (aa 1–1061; full-length) fusion in pUT18C	[32]
pYG100	encodes T25-RNase E (aa 1–1061; full-length) fusion in pKT25	[34]
pYG101	encodes T25-RNase E (aa 1–597) fusion in pKT25	[34]
pYG102	encodes T25-RNase E (aa 499–1061) fusion in pKT25	[34]
pYG115	encodes T25-CsrD fusion in pKT25	this work
pYG116	encodes T18-CsrD fusion in pUT18C	this work
pYG121	encodes T25-RNase III fusion in pKT25	this work
pYG123	encodes T25-RNase G fusion in pKT25	this work
pYG124	encodes T18-RNase G fusion in pUT18C	this work
pYG137	encodes T25-RtcB fusion in pKT25	this work
pYG139	encodes T25-CorA fusion in pKT25	this work
pYG141	encodes T25-YegJ fusion in pKT25	this work
pYG144	encodes T18-RNase E (aa 1–415) fusion in pUT18C	this work
pYG147	encodes T25-YohP fusion in pKT25	this work
pYG151	encodes T25-GltP fusion in pKT25	this work
pYG152	encodes T25-DacC fusion in pKT25	this work
pYG155	encodes T25-MurG fusion in pKT25	this work
pYG157	encodes T25-PstC fusion in pKT25	this work
pYG158	encodes T25-YqjF fusion in pKT25	this work
pYG159	encodes T25-FliD fusion in pKT25	this work
pYG162	encodes T18-RNase E (aa 598–1061) fusion in pUT18C	this work
pYG163	encodes T18-RNase E (aa 586–1061) fusion in pUT18C	this work
pYG165	encodes T18-SlyX fusion in pUT18C	this work
pYG166	encodes T18-YdfR fusion in pUT18C	this work
pYG168	encodes T18-RNase E (aa 1–498) fusion in pUT18C	this work
pYG174	encodes T25-SlyX fusion in pKT25	this work
pYG175	encodes T25-YdfR fusion in pKT25	this work
pYG181	*strep-slyX* under *P*_*tac*_ control, *lacI*^*q*^, *bla*, ori ColEI	this work
pYG182	*strep-ydfR* under *P*_*tac*_ control, *lacI*^*q*^, *bla*, ori ColEI	this work
pYG191	*qseG*-strep under *P*_*tac*_ control, *lacI*^*q*^, *bla*, ori ColEI	[50]
pYG195	encodes T18-RNase E (aa 1–597; F574A and F575A substitutions) fusion in pUT18C	this work
pYG206	*yegJ-strep* under *P*_*tac*_ control, *lacI*^*q*^, *bla*, ori ColEI	this work
pYG286	encodes T25-RseA fusion in pKT25	this work
pYG287	encodes T25-ThrB fusion in pKT25	this work
pYG307	*strep-thrB* under *P*_*tac*_ control, *lacI*^*q*^, *bla*, ori ColEI	this work
pYG308	encodes T25-WcaF fusion in pKT25	this work
pYG309	encodes T25-YcbZ fusion in pKT25	this work
pYG310	encodes T25-DcuA fusion in pKT25	this work

### *Transformation of* E. coli *by electroporation*

The *E. coli* strain to be made electro-competent was inoculated in 5 ml SOB medium containing the required antibiotics and grown overnight at 37°C or at 28°C for transformants of strain BTH101. On the next day, 200 µl of the overnight culture were used to inoculate 50 ml fresh SOB medium in a 250 ml conical flask and the culture was grown at the required temperature until the OD_600_ reached 0.6–0.7. The culture was transferred to a pre-chilled 50 ml Falcon centrifuge tube, placed on ice for 1 h, and subsequently centrifuged (4000 rpm, 15 min, 4°C). Cells were resuspended in 50 ml ice-cold H_2_O, and the centrifugation step was repeated. Subsequently, cells were resuspended in 25 ml ice-cold H_2_O and pelleted once more. Cells were resuspended in 2 ml ice-cold 10% glycerol. Following an additional centrifugation (6000 rpm, 15 min, 4°C) and resuspension in 200 µl ice-cold 10% glycerol, the cells were suitable for immediate transformation by electroporation. Alternatively, cells were frozen and stored for later transformation. To this end, cells were incubated for 1 additional h on ice, split into 40 μl aliquots and kept at −80°C following shock-freezing in liquid nitrogen.

For electroporation, the DNA (10 ng in 1 μl was used in case of the genomic library in pKT25) was provided in a pre-chilled 1.5 ml tube on ice, 40 μl competent cells were added, and the mixture was transferred into a pre-chilled 0.1 cm electro-cuvette. Following the electroshock at ~1.8 kV for 5–6 ms (Eppendorf Eporator), 1 ml SOC medium was added immediately, and the cell suspension was incubated at 37°C for 1 h or at 28°C for 2 h in case of BTH101 transformations. For screening the genome library in pKT25, cells were pelleted by centrifugation and resuspended in 500 μl Mg-saline (145 mM NaCl, 10 mM MgSO_4_), of which 100 μl was plated per M63 maltose plate with antibiotics.

### *Construction of two-hybrid libraries in plasmid pKT25 with genomic DNA from* E. coli

Genomic DNA was isolated from 4 ml overnight culture of *E. coli* strain W3110 using the PrestoSpin D Kit (Molzym) according to the instructions of the manufacturer. 20 μg of the genomic DNA eluted in dH_2_O was subjected to partial digest using NEBNext dsDNA Fragmentase (NEB) in a volume of 100 μl containing 1×Fragmentase buffer, 0.1 mg/ml BSA, 10 mM MgCl_2_ and 10 μl Fragmentase at 37°C for 15–20 min. The reaction was stopped by adding 5 μl 0.5 M EDTA pH 8.0. The DNA was precipitated by adding 2.5 volumes ethanol and incubation for 2 h at −20°C. Following centrifugation at 4°C for 30 min, the DNA pellet was washed with 100 μl 70% ethanol, dried and resuspended in 20 μl dH_2_O. The digested genomic DNA was separated on a 1% agarose gel and DNA fragments in size range of 100 bp-1500 bp were excised, purified with the NucleoSpin Gel and PCR Clean-up kit (Macherey-Nagel) and finally eluted in 35 μl dH_2_O. Subsequently, the genomic DNA fragments were converted into blunt-end DNA and provided with phosphate groups at the 5’ ends using the Fast DNA End Repair Kit (Thermo Scientific). To this end, 500 ng DNA fragments were incubated in 50 μl volume with 1× End Repair Reaction Mix and 2.5 μl End Repair Enzyme Mix for 10 min at 20°C and subsequently purified using the NucleoSpin Gel and PCR Clean-up kit (Macherey-Nagel). Following digestion with SmaI, extensive treatment with alkaline phosphatase and purification, 35 ng of the linearized pKT25 plasmid was ligated with a two-fold molar excess of the genomic DNA fragments in a volume of 20 μl. The ligation products were purified using the NucleoSpin Gel and PCR Clean-up kit (Macherey-Nagel) and subsequently eluted in 15 μl H_2_O, of which 1 μl was used to transform 40 μl electrocompetent DH5α cells via electroporation. Following the recovery phase in SOC medium, 50 μl of the cell suspension was removed and directly plated on selective plates using appropriate dilutions to calculate cloning efficiency, which varied between 5.000–47.000 recombinants/ml. A representative number of colonies was checked by analytic PCR for presence and sizes of inserts, which usually ranged in between 100–1000 bp (Suppl. Figure S1). The remaining 950 μl cell suspension was used to inoculate 200 ml LB containing kanamycin and incubated overnight to expand the library. On the next day, plasmids were extracted using the Macherey-Nagel NucleoSpin Plasmid Midi-Prep Kit. Six such plasmid preparations with ~10% insert-less plasmids, each derived from independent ligations and transformations, were pooled (5 μg of each preparation) representing one final genomic library in pKT25. Altogether, 5 such genome libraries were constructed of which three designated GB1, GB4 and GB5 were used for screening. These libraries contained ~150.000 individual plasmid recombinants as deduced from the cloning efficiency calculations. A flow chart illustrating the major steps of construction of the genome libraries and of the subsequent screening procedure is shown in Suppl. Figure S2.

### Screening the genomic library in pKT25 for candidate interactors of Rne and Rng

For screening of the genome library in pKT25, strain BTH101 carrying the pUT18C-*rne* bait plasmid was transformed by electroporation using 10 ng of the genome library and plated on M63-maltose minimal plates as described above. For selection and screening, the plates contained 50 μg/ml ampicillin, 15 μg/ml kanamycin, 1 mM IPTG and 40 μg/ml X-Gal. Blue candidate colonies usually appeared after 1–4 days incubation at 28°C. Candidate colonies were re-streaked and incubated alongside the BACTH positive control (BTH101 carrying pUT18C-zip + pKT25-zip) on the same type of plates to confirm the phenotype, i.e. robust growth and blue colouration following incubation at 28°C for 2–3 days. Subsequently, single colonies were inoculated in 5 ml LB medium supplemented with kanamycin and ampicillin. Plasmids were extracted and used to transform BTH101 once again using standard procedures [55,56] and selection on LB plates containing ampicillin, kanamycin and X-Gal at 28°C for 30–48 h. The latter step was necessary to identify screening candidates that carried mixtures of different pKT25 derivatives (Suppl. Figure S1), which resulted in non-uniform phenotypes. For separation, the latter procedure was repeated until all colonies exhibited a uniform blue phenotype. Finally, the inserts of the isolated pKT25 recombinants were amplified by PCR using oligonucleotides BG646+BG647. PCR products were analysed by agarose gel electrophoresis, purified using the NucleoSpin Gel and PCR Clean-up kit (Macherey-Nagel) and sequenced using primers BG646 and BG647.

### Spotting assays for phenotypic evaluation of interactions using the BACTH system

For spotting assays, strain BTH101 was made competent using a standard procedure [56] and subsequently doubly transformed with the desired combinations of pKT25- and pUT18C-derivatives in one step. 25 µl of the freshly prepared competent cells was mixed with 7.5 ng (in 0.5 μl) of each plasmid and incubated for 20 min on ice. Following a heat shock at 42°C for 90 sec and an additional incubation on ice for 20 min, 200 µl LB was added and cells were incubated at 30°C for 2 h. 5 µl of the cell suspension were spotted alongside other BACTH double transformants on LB-plates containing 100 µg/ml ampicillin, 30 µg/ml kanamycin, 1 mM IPTG and 40 µg/ml X-Gal or on MacConkey-agar plates containing 100 µg/ml ampicillin, 30 µg/ml kanamycin, 1 mM IPTG and 0.2% maltose as carbon source. The plates were incubated at 28°C for ~30 h. BTH101 cells carrying plasmids pUT18C-zip and pKT25-zip served as positive control. The latter plasmids encode fusions of the leucine zipper domain of yeast transcription factor Gcn4 to T18 and T25, respectively. Cells carrying the empty plasmids pUT18C and pKT25 served as negative controls.

### β-Galactosidase activity assay for quantitative assessment of interactions in the BACTH system

BTH101 cells carrying the desired pUT18C and pKT25 derivatives expressing the fusion proteins of interest were grown for ~16 h at 28°C in LB-Lennox medium supplemented with the required antibiotics and with 1 mM IPTG to stationary phase. Subsequently, the β-galactosidase activities were measured as described [57]. Presented values present the average of at least two measurements using independent transformants.

### Ligand fishing using StrepTactin affinity chromatography

Copurification experiments addressing interaction of Strep-tagged bait proteins with the FLAG-tagged Rne_1–597_ variant were performed as described previously [34], except that strain Z899 was used.

### SDS-PAGE and western blotting

SDS-PAGE and subsequent western blotting were carried out as described previously [34,50].

### Purification of Strep- and His_6_-tagged proteins

Strep-tagged PtsN, RapZ, YegJ and His_6_-tagged Rne_1–529_ were purified following previously established protocols [34]. Strep-RapZ and Strep-PtsN were overproduced in strain Z899 using plasmid pBGG164 and pBGG217, respectively. Strain BL21 (DE3) was used to overproduce YegJ-strep from plasmid pYG206. The transformants were grown in LB and protein synthesis was induced with 1 mM IPTG when cultures reached an OD_600_ = 0.5–0.8. Following an additional 1 h of growth, cells were harvested, pelleted and resuspended in buffer W (100 mM Tris-HCl pH 8,0, 150 mM NaCl, 1 mM EDTA). Cells were disrupted using a French pressure cell and lysates were cleared by centrifugation (4000 rpm, 4°C, 20 min, Eppendorf 5810 R and 14,000 rpm, 4°C, 1 h, Eppendorf 5427 R). Thereafter, lysates were loaded on poly-prep chromatography columns (Bio-Rad) containing pre-equilibrated StrepTactin sepharose (IBA, Germany). The volume of the latter corresponded to 0.1% of the original culture volume. The columns were 4× washed using 10 column bed volumes (CBV) of buffer W each and proteins were eluted in three steps using 1 CBV of buffer W containing 2.5 mM D-desthiobiotin, respectively. The elution fractions with the highest protein yield were 2× dialysed for 20 h at 4°C against a buffer containing 10 mM Tris-HCl pH 7.0, 100 mM KCl, 10 mM MgCl_2_, 2 mM β-mercaptoethanol.

For purification of His_6_-tagged Rne (1–529), BL21 cells carrying plasmid pRne529-N were grown and harvested by centrifugation as described for Strep-tagged proteins. Following resuspension in ZAP buffer (50 mM Tris-HCl pH 7.5, 200 mM NaCl) and cell disruption, lysates were cleared by centrifugation (4000 rpm, 4°C, 20 min, Eppendorf 5810 R and 35,000 rpm, 4°C, 1 h, Beckman 60TI) and loaded on chromatography columns containing 3 ml Ni-NTA superflow suspension (IBA, Germany) equilibrated with ZAP buffer. Columns were 5× washed using ZAP buffer containing incremental imidazole concentrations (5, 10, 25, 50, 80 mM) and proteins were finally eluted in three steps using ZAP buffer containing 125 mM, 250 mM and 500 mM imidazole, respectively. The 250 mM fraction contained the highest yield of His_6_-Rne_NTD_ and was 2× dialysed for 20 h at 4°C against a buffer containing 20 mM Tris-HCl pH 7.9, 500 mM NaCl, 10 mM MgCl_2_, 0.5 mM EDTA, 20 mM β-mercaptoethanol. For storage, the protein preparations were mixed with 5% or 10% glycerol and kept at −80°C.

### In vitro *pull-down assay using Strep-XT magnetic agarose beads*

The purified Strep-tagged bait proteins (3 μg each) were coupled to 10 μl Strep-Tactin ‘type 3’ XT magnetic beads (IBA, Germany) by incubation in 250 μl interaction buffer (100 mM Tris/HCl pH8.0, 150 mM NaCl, 0.05% Tween 20) for 1 h at 4°C on an end-over-end shaker. Subsequently, the supernatants were removed and 3.5 μg of the purified His_6_-tagged Rne_1–529_ prey protein provided in 250 μl interaction buffer was added. The mixtures were incubated at RT for 1.5 h and subsequently the beads were 4× washed using 500 μl interaction buffer each. For protein elution, the magnetic beads were re-suspended in 50 μl Laemmli buffer and incubated for 5 min at 40°C. 10 μl of the eluates were separated on SDS-PAA gels, followed by Coomassie blue silver staining of the gels [58]. For removal of RNA, purified His_10_-RNase E_1–529_ and YegJ-Strep were preincubated for 1 h at 37°C with 600 units micrococcal nuclease (NEB) in 1× reaction buffer and subsequently used for the pull-down assay.

### Extraction of nucleic acids from elution fractions obtained by StrepTactin affinity chromatography

For extraction of nucleic acids copurifying with proteins upon StrepTactin affinity purification, 250 μl of the elution fraction with the highest protein yield was extracted using 250 μl phenol:chloroform:isoamylalcohol (25:24:1) and the nucleic acid fraction in the supernatant was precipitated using 750 μl ethanol and 25 μl 4 M LiCl. Following incubation overnight at −20°C, the pellet was washed with 70% ethanol, air-dried, and finally dissolved in 20 µl RNase-free water. Nucleic acid concentrations were determined using a NanoDrop spectrophotometer.

### Extraction of total RNA and whole transcriptome analysis by RNA sequencing

The transcriptome analyses in the current study were carried out in parallel and in the same manner as the RNA-seq analyses of RapZ overproducers, the results of which have been published [34]. RNA extraction, library construction, RNA-sequencing and bioinformatic analyses were carried out as described in the previous study. The raw sequencing data were deposited to the European Nucleotide archive (ENA; https://www.ebi.ac.uk/ena) under the accession number PRJEB108935.

### Envelope permeability assay

Envelope permeability was determined by adopting a method that measures availability of externally provided ortho-nitrophenyl-β-galactoside (ONPG) as substrate for β-galactosidase produced in the cytoplasm [59]. *E. coli* strain T3276 was used, which transcribes *lacZ* from the constitutive *P*_*16*_ promoter and lacks LacY, which is known to transport ONPG [60]. Transformants of this strain carrying the plasmids to be tested were grown to OD_600_ ~0.3, when the culture was split and either received 1 mM IPTG for induction of plasmid-encoded genes or H_2_O as mock. Samples of the cultures were harvested 30 min later and appropriate dilutions were subjected to classical β-galactosidase assays including an SDS/CHCl_3_ permeabilization step as described [57], quantifying the total β-galactosidase activities produced by the cells. In parallel, a similar assay was performed but the membrane permeabilization step was omitted, allowing to assess envelope permeability. The measured ONPG hydrolysis rates are expressed in ‘Miller units’.

## Results

### Validation of T18-RNase E fusion constructs and characterization of CsrD-RNase E interaction

First, we tested the functionality of pUT18C BACTH constructs encoding various truncated Rne variants fused to the C-terminus of the CyaA T18 domain (Figure 1(A,B)). This step served to validate suitability of the constructs as potential baits in the subsequent library screens and to confirm their functionality for later characterization of identified prey proteins. To this end, we tested their interaction with T25-RapZ and T25-enolase fusion proteins in strain BTH101 by BACTH. Interaction of RapZ and enolase with Rne is established and has been characterized previously (Figure 1(A,B)).

Quantitative and phenotypic BACTH assays demonstrated interaction of the T25-RapZ construct with all T18-Rne constructs that retained at least the large domain and the Zn^2+^-link, i.e. full-length Rne (Rne_FL_), Rne_1–597_, Rne_1–498_ and Rne_1–415_ (Figure 1(B,C)). No interaction was detectable when T25-RapZ was combined with T18 constructs comprising only the scaffolding domain of Rne. The latter double transformants produced colourless colonies on LB X-Gal plates and their β-galactosidase activities did not exceed the activity of the negative control i.e. strain BTH101 carrying the empty plasmids pUT18C/pKT25, which produced 91 ± 16 Miller units. Additionally, we included a T18-Rne_1–597_ construct carrying alanine substitutions of phenylalanine residues 574 and 575 (subsequently designated Rne_1–597_-FF/AA) within the MTS, previously described to diminish membrane association of Rne [21,61]. These substitutions did not affect the interaction of Rne_1–529_ with RapZ, corroborating that membrane association plays no role. These data are in agreement with previous results showing that RapZ binds the Rne catalytic domain [32,34] by making direct contacts to residues 311–316 [3]. In contrast, the T25-enolase construct showed interaction only with the T18-Rne constructs that contain the scaffolding domain and include residues 834–850 – the previously mapped enolase binding site [62]. β-Galactosidase activities dropped ~2-fold when T25-enolase was combined with the T18-Rne_586–1061_ or the T18-Rne_598–1061_ constructs as compared to the longer T18-Rne_499–1061_ fusion (Figure 1(B,C)), suggesting that the presence of residues 499–586 including the MTS improves the Rne/enolase interaction. Taken together, the various T18-Rne constructs show the expected interaction characteristics, confirming their functionality.

As interaction of CsrD with Rne was to the best of our knowledge previously not addressed, we also included CsrD in the analysis. The BACTH assay revealed interaction of CsrD with Rne_FL_, Rne_1–597_ and with Rne_499–1061_ but not with any other truncation (Figure 1(B,C)). The interactions were retained when the T25- and T18-domains were swapped between CsrD and Rne constructs (Figure 1(D)). Other RNases such as RNase III (Rnc) or RNase G (Rng), which has sequence similarity to Rne [63], showed no interaction suggesting that the CsrD/Rne interaction as monitored by BACTH is specific. Hence, CsrD and RapZ bind Rne differently: while RapZ contacts the large globular domain of Rne (Figure 1(A)), CsrD requires Rne residues 499–597 including the MTS for interaction.

### Construction and screening of BACTH genomic libraries for identification of proteins interacting with Rne

We hypothesized that there are more proteins in addition to RapZ and CsrD that contact the Rne catalytic domain to alter its activity or to target selected transcripts to decay. To identify such proteins, we constructed a genomic library in the BACTH vector pKT25 and screened for candidate interactors using various T18-Rne constructs as bait (Suppl. Figure S2 [40];). Briefly, chromosomal DNA extracted from *E. coli* K12 strain W3110 was randomly fragmented, DNA fragments were isolated, blunt-end repaired and subsequently shot-gun cloned into the SmaI site of plasmid pKT25. Three genome libraries named GB1, GB4 and GB5 were constructed independently and used for screening. Each library contained ~150.000 individual recombinant plasmids as estimated from plating efficiencies. However, the fraction of clones containing genes or gene fragments fused in frame to *T25* is expected to be significantly lower, considering possible reading frames and ligation of non-coding DNA strands to the *T25 orf*.

The gene libraries were introduced into the BACTH strain BTH101 carrying the *T18-rne* bait plasmid by electroporation and cells were plated on M63-maltose minimal medium containing X-Gal, IPTG and required antibiotics, thereby selecting for growth of doubly transformed bacteria capable of synthesizing cAMP. Typically, colonies able to grow on maltose appeared after 2–4 days incubation at 28°C. Blue candidate colonies were re-streaked on fresh M63-maltose minimal plates to confirm robust growth and maintenance of the blue phenotype. Transformants that passed this test were subjected to plasmid extraction and isolated plasmids were used to transform BTH101 once again followed by selection on LB X-Gal plates to confirm uniformity of the phenotype. Subsequently, the inserts of positive colonies were amplified by PCR and sequenced. The identified protein fragments, the functions and localization of corresponding full-length proteins and the frequencies with which particular candidate clones were identified are listed in Table 2. Additional information on genome coordinates of inserts, used library and bait constructs as well as isolate names is presented in Suppl. Table S3.

**Table 2.** Proteins and protein fragments identified in the BACTH screens for candidate interactors of Rne.

Protein	UniProt acc. no	Function ^a^	Loc. ^a^	Residues (no. of MDs) fused to T25 ^a, b^	No. ^c^	Validation ^d^	Gr. ^e^
Aer	P50466	aerotaxis receptor	IM	36–220 (2)	1	N/A	N/A
AmpE	P0AE14	uncharacterized	IM	219–284 (1)	1	N/A	N/A
BaeS	P30847	sensor histidine kinase	IM	1–36 (1)	1	N/A	N/A
CadC	P23890	transcriptional activator	IM	106–175 (1)	2 (1)	N/A	N/A
CorA	P0ABI4	Ni^2+^/Co^2+^/Mg^2+^ transporter	IM	118–316 (2)	15 (2)	FL+	I
CydH	A5A618	cytochrome *bd*-I accessory SU	IM	1–29 = FL (1)	1	FL+	I
DacA	P0AEB2	penicillin-binding protein 5	IM	347–403 (1); 385–403 (1)	1; 2 (1)	N/A	N/A
DacC	P08506	penicillin-binding protein 6	IM	270–400 (1)	2 (2)	FL-	N/A
DcuA	P0ABN5	C4-dicarboxylate transporter	IM	377–433 (1)	2 (2)	FL-	N/A
DgcN	P46139	diguanylate cyclase	IM	15–189 (4)	1	N/A	N/A
EptA	P30845	phosphoethanolamine transferase	IM	128–271 (2)	1	N/A	N/A
FliD	P24216	flagellar filament capping protein	EC	266–420	38 (2)	FL-	N/A
FtsN	P29131	cell division protein	IM	22–197 (1)	1	N/A	N/A
GlpC	P0A996	anaerobic glycerol-3-P DH SU C	IM	1–69	1	N/A	N/A
GltP	P21345	glutamate/aspartate:H^+^ symporter	IM	1–437 = FL (10); 255–434 (4)	19 (3); 1	FL+	I
MurG	P17443	N-acetylglucosaminyl transferase	IM	2–133	2 (1)	FL+	I
NarQ	P27896	sensor histidine kinase	IM	1–32 (1)	2 (1)	N/A	N/A
NimT	P76242	2-nitroimidazole exporter	IM	4–393 (12)	1	N/A	N/A
PstC	P0AGH8	phosphate ABC transporter SU	IM	14–35 (1)	2 (2)	FL+	I
Rne	P21513	ribonuclease E	IM	506–598 (1)	2 (1)	N/A	N/A
RseA	P0AFX7	anti-sigma-E factor	IM	63–216 (1)	9 (1)	FL-	N/A
RtcB	P46850	3’−5’ RNA ligase	Cyt	113–205	1	FL-	N/A
SlyX	P0A8R4	uncharacterized	Cyt	1–72 = FL	1	FL+, pull-d.	II
ThrB	P00547	homoserine kinase	Cyt	268–310	4 (1)	FL+, pull-d.	II
TorC	P33226	cytochrome c menaquinol DH	IM	1–124 (1)	1	N/A	N/A
TtdT	P39414	L-tartrate:succinate antiporter	IM	455–487 (1)	3 (2)	N/A	N/A
UbiD	P0AAB4	3-octaprenyl-4-hydroxybenzoate DC	IM	344–393	1	N/A	N/A
UspB	P0A8S5	universal stress protein	IM	50–111 (1)	1	N/A	N/A
WcaF	P0ACD2	colanic acid acetyltransferase	Cyt	10–182	1	FL+	III
XapR	P23841	transcriptional activator	Cyt	98–294	1	N/A	N/A
YbbP	P77504	putative ABC transporter SU	IM	1–267 (2)	1	N/A	N/A
YbhG	P75777	HlyD_D23 family protein	IM/PP	1–97	1	N/A	N/A
YcbZ	P75867	putative ATP-dependent protease	Cyt	565–586	1	FL+	III
YdiN	P76198	putative transporter	IM	193–382 (6)	1	N/A	N/A
YdfR	P76160	Qin prophage protein	Cyt	1–72	3 (2)	FL+, pull-d.	II
YegJ	P76394	DUF2314 domain protein	Cyt	16–153	48 (3)	FL+, pull-d.	II
YfcC	P39263	putative transporter	IM	464–506 (1)	1	N/A	N/A
YfgM	P76576	ancillary SecYEG translocon SU	IM	1–206 = FL (1)	1	FL+	I
YfjP	P52131	putative GTP-binding protein	Cyt	259–287	2 (1)	N/A	N/A
YgaZ	P76630	putative L-valine exporter SU	IM	107–249 (4)	1	N/A	N/A
YjhF	P39357	putative transporter	IM	414–449 (1)	1	N/A	N/A
YohP	C1P609	uncharacterized membrane protein	IM	1–27 = FL (1)	3 (1)	FL+	I
YphA	P0AD47	putative inner membrane protein	IM	1–140 = FL (4)	5 (1)	FL+	I
YqjF	P42619	DoxX family protein	IM	1–35 (1)	2 (2)	FL+	I

Abbreviations: Cyt = cytosol, DC = decarboxylase, DH = dehydrogenase, EC = extracellular, FL = full-length, IM = cytoplasmic membrane, PP = periplasm, SU = subunit.

^a^Information on protein function, localization (loc.) and location of membrane domains (MDs) are according to annotations in the Ecocyc [64] and UniProt databases.

^**b**^The amino acid residues that were found to be fused to the T25 domain in the clones identified in the BACTH screens are indicated. The number of membrane domains (MDs), i.e. *trans*-membrane domains or other membrane anchoring domains contained within the recovered peptide, is indicated in parentheses.

^c^Frequency with which the candidate was identified in total. The number in parentheses indicates how often the candidate was identified in independent screens using different BACTH libraries or bait constructs.

^d^N/A = not available, FL+ = validated to bind Rne when tested as full-length protein by BACTH, FL- = failed to bind Rne when tested as full-length protein by BACTH, pull-d. = validated by pull-down or ligand-fishing approaches.

^e^Grouping of the candidate interactors according to BACTH analysis using various Rne truncations (Figure 2; current work).

**Figure 2.**
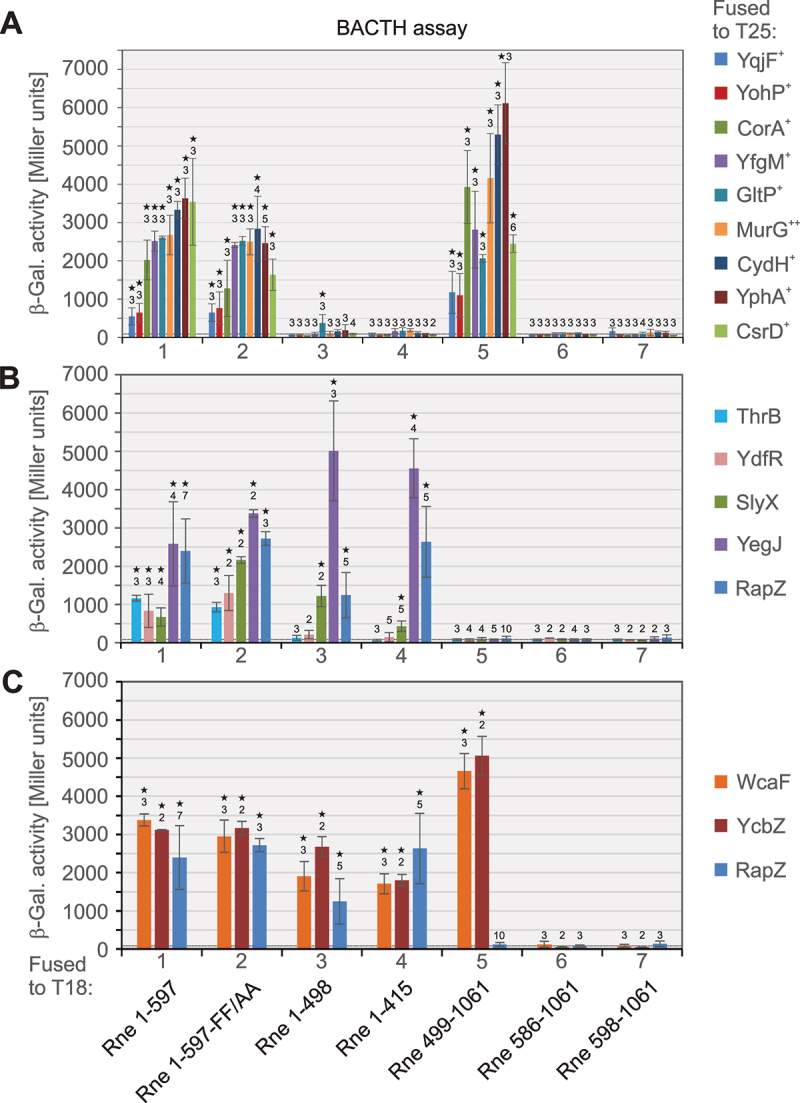
Quantitative BACTH assays addressing interaction of the full-length versions of various prey proteins identified in the BACTH screen with various Rne truncations. Double transformants of strain BTH101 were tested, which carried the pKT25 derivatives encoding the various prey proteins fused to T25, each combined with a pUT18C derivative encoding a particular T18-Rne variant, as follows: pYG97 (T18-Rne_1–597_, columns 1), pYG195 (T18-Rne_1–597 FF/AA_, columns 2), pYG168 (T18-Rne_1–498_, columns 3), pYG144 (T18-Rne_1–415_, columns 4), pYG98 (T18-Rne_499–1061_, columns 5), pYG163 (Rne_586–1061_, columns 6) and pYG162 (T18-Rne_598–1061_, columns 7). The double transformants were grown in LB containing 1 mM IPTG for ~16 h at 28°C and the β-galactosidase activities were determined, which are presented as mean ± standard deviation. Replicate numbers (*n*) are indicated. The measurements were analysed by paired two-tailed t-test and activities significantly higher than the negative control as provided by the empty vectors pKT25/pUT18C are labelled with asterisks (*p* < 0.05). The activity produced by the negative control is indicated by a dotted horizontal line in the graphs. Proteins with transmembrane domains are indicated with ‘+’, proteins associated with the IM are labelled with ‘++’. (A) The following pKT25 derivatives encoding the indicated T25-prey protein fusions were tested (referring to the individual columns from left to right in 1–7, respectively): pYG158 (T25-YqjF), pYG147 (T25-YohP), pYG139 (T25-CorA), pYG195D_c87 (T25-YfgM), pYG151 (T25-GltP), pYG155 (T25-MurG), pYG195D_c36 (T25-CydH), pYG195D_c5 (T25-YphA), pYG115 (T25-CsrD). (B) The following pKT25 derivatives encoding the indicated T25-prey protein fusions were tested: pYG287 (T25-ThrB, blue columns), pYG175 (T25-YdfR, green columns), pYG141 (T25-YegJ, purple columns), pBGG348 (T25-RapZ, dark blue columns). (C) The following pKT25 derivatives encoding the indicated T25-prey protein fusions were tested: pYG308 (T25-WcaF, orange columns); pYG309 (T25-YcbZ, red columns); pBGG348 (T25-RapZ, dark blue columns).

### BACTH screen of genomic libraries identifies various candidate interactors for RNase E, most of which are membrane proteins

We performed several rounds of screenings of the three gene libraries using the C-terminally truncated T18-Rne_1–597_ variant that lacks most of the scaffolding domain as bait. Collectively, the isolated prey clones contained fragments of 22 *orfs* that were fused in frame to *T25* in all cases (Suppl. Table S3). Nine of these inserts were identified repeatedly, emphasizing that they are likely true hits, i.e. fragments of *dacC* (penicillin-binding protein 6; 2×), *fliD* (flagellar filament capping protein; 34×), *gltP* (glutamate/aspartate transporter; 18×), *murG* (N-acetylglucosaminyl transferase; 2×), *pstC* (membrane domain of ABC phosphate transporter; 2×), *ttdT* (tartrate/succinate antiporter; 3×), *yegJ* (uncharacterized protein; 11×), *yohP* (uncharacterized membrane protein; 3×) and *yqjF* (uncharacterized IM protein; 2×). The remaining inserts, i.e. fragments of *aer*, *ampE*, *baeS*, *dacA*, *dcuA*, *dgcN*, *nimT*, *ubiD*, *wcaF*, *ybbP*, *ycbZ*, *ygaZ* and *yjhF,* were identified only once (Suppl. Table S3). Additionally, we performed a preliminary screen using T18-Rne_FL_ as bait, which yielded fragments of *corA* (divalent metal ion transporter; identified 14×), *eptA* (phosphoethanolamine transferase), *rtcB* (RNA ligase), *ydiN* (putative transporter) and the same fragment of *yegJ* (identified 3×) that was already identified before with T18-Rne_1–597_ as bait (Suppl. Table S3).

Most of the inserts encode fragments of proteins that contain at least one transmembrane domain (TMD) or encode fragments of proteins that are likely associated with the membrane such as MurG and UbiD (Table 2). The only exceptions are the inserts derived from *fliD*, *rtcB*, *wcaF*, *ycbZ* and *yegJ*. Considering that the used bait constructs contain the MTS of Rne and are likely membrane-associated, we questioned whether our screen is biased towards identification of membrane-localized proteins, reflecting that their diffusion is restricted to the two-dimensional space. To avoid this potential bias, we performed additional screens using Rne_1–415_ as bait, which lacks the MTS. Thereby, we identified 34× the *yegJ* fragment found already before, but only 3 additional fragments derived from genes *dcuA* (1×), *glpC* (1×) and *gltP* (1×) encoding once again membrane (−attached) proteins. Notably, the latter insert comprised only the 3’ half of *gltP* whereas full-length *gltP* was identified in the previous screens, suggesting that the site interacting with Rne resides in the C-terminal portion of the protein. Thus, the screens with RNase E_1–415_ yielded only *glpC* as novel candidate, which encodes a subunit of a glycerol-3-phosphate dehydrogenase.

We reasoned that either a too short bait sequence and/or an overrepresentation of the *T25-yegJ* clones in the library were limiting the results obtained in the screens with the Rne_1–415_ variant as bait. To account for these possible bottlenecks, we chose the Rne_1–597_-FF/AA variant (diminished membrane association) as bait. Furthermore, we pooled equal amounts of gene libraries GB1, GB4 and GB5 and treated the mix with the restriction enzyme BstZ17I to deplete the library from plasmids carrying T25-*yegJ*. BstZ17I cleaves in the 3’ end of *yegJ* but not elsewhere in pKT25. Using this set-up, we performed additional screens, which yielded novel candidates, but also recovered previously identified ones except for *yegJ*, reflecting successful depletion of corresponding plasmids (Suppl. Table S3). We identified once more *corA* (1×), *fliD* (4×), *gltP* (1×) and *dacA* (1×; penicillin-binding protein 5), but the latter represented by a different insert than in the previously identified clone. Additionally, we identified novel fragments corresponding to the DNA-binding transcriptional activators CadC (2×) and XapR (1×), the accessory subunit CydH of the cytochrome bd-I complex (1×), the cell division protein FtsN (1×), the histidine kinase NarQ (2×), the anti-sigma E factor RseA (9×), the homoserine kinase ThrB (4×), the cytochrome c menaquinol dehydrogenase TorC (1×), the SecYEG translocon subunit YfgM (1×) and the uncharacterized proteins YbhG (1×), YfcC (1×), YfjP (2×) and YphA (5×). Once again, most of these proteins are known to localize within or at the cytoplasmic membrane except for FliD, ThrB, XapR and YfjP (Table 2). Interestingly, we also identified twice a fragment comprising codons 506–598 of Rne suggesting that this region contributes to Rne self-interaction.

While the isolates described above carry annotated *orfs* fused in frame to *T25*, we additionally obtained 15 clones (out of 208 isolates in total ≈7.2%) that did not contain an in-frame fusion of an annotated *orf* (Suppl. Table S4). Instead, they encode non-annotated peptides or proteins derived from alternative reading frames or from the opposite strand of coding genes fused to T25. Such clones were obtained with every bait except for Rne_1–415_. The transformants that carried these plasmids together with the bait constructs required somewhat longer incubation times, i.e. 3–4 days until they formed visible blue colonies on M63 maltose plates, suggesting weaker interactions. Analysis of these peptides and proteins using the ProtParam tool revealed that they are enriched in positively charged Arg and Lys residues and are therefore all characterized by a high pI ranging from 7.8–12.45 (Supplementary Results). In contrast, the region comprising residues 416–597 of Rne is highly enriched in negatively charged residues (i.e. 22×Glu, 3×Asp) and has a pI of 5.2. It appears conceivable that these peptides bind Rne through ionic interactions. It is questionable whether these interactions occur in the living cell, albeit we cannot exclude that some of these *orfs* are translated, considering the ongoing identification of so far unrecognized microproteins [65]. In this context, it might be noteworthy that one of these clones contained 71 codons derived from the opposite strand of the *rne* gene itself. Closer inspection revealed that these 71 codons are part of a 551 codon long hypothetical *orf* on the DNA strand opposite to the 3’ half of *rne* (Supplementary Results). An amenable Shine-Dalgarno sequence (AGGAGC) is present 9 bp upstream of the first methionine codon occurring in this *orf*, providing the possibility that it is translated into a yet non-annotated protein.

### A BACTH library screen using T18-Rng as bait identifies YdfR and SlyX as candidate interactors of Rne

Rng is a paralog of Rne and shares 34% sequence similarity with the catalytic domain of RNase E [63], while lacking a C-terminal scaffolding domain. We wondered whether Rng might also be contacted by other proteins as observed for the Rne-NTD. Therefore, we additionally screened our BACTH libraries using the T18-Rng construct pYG124 as bait. Following four rounds of screening of ~10^8^ transformants in total, we obtained six positive clones that retained the ability to confer a blue phenotype and to mediate growth on M63 minimal plates following re-streaking and re-transformation of BTH101 with the isolated plasmids. Two of these clones carried the almost complete *cyaA* gene (codons 1–802 out of 848 codons) oriented in opposite direction to the *T25* fragment (Suppl. Table S4). Expression of this fragment from a plasmid-located promoter likely complements the *cyaA* deletion of BTH101, explaining the phenotype. One clone carried *slyX*, a 72 codon long gene of unknown function, fused in frame with *T25*. The three remaining clones were identical and comprised the first 72 codons of *ydfR*, a 309 bp long gene of unknown function. In this case, the linker connecting the *ydfR* 5’ fragment in frame with *T25* additionally included 53 bp provided by the *ydfR* 5’ UTR (Suppl. Table S3).

To assess the interaction properties of SlyX and YdfR further, we cloned the full-length genes into plasmid pKT25. This construction fuses the gene of interest to the *T25* fragment through an only 15 bp long linker. Subsequently, we tested these fusions for interaction with T18-Rng as well as T18-Rne, through quantitative BACTH assays (Suppl. Figure S3). The results indicate an interaction of SlyX with Rng of moderate strength. T25-RapZ, which was included for comparison, did not produce activities above background when combined with T18-Rng, confirming that RapZ cannot bind Rng (Figure 1(D)). Interestingly, T25-SlyX produced higher activities when combined with T18-Rne_FL_ or T18-Rne_1–597_ rather than with T18-Rng suggesting that SlyX preferably binds Rne. Surprisingly, the cloned T25-YdfR fusion (short linker) produced activities above background only in combination with the T18-Rne_1–597_ construct, but not with the two other fusions. In contrast, the T25-YdfR (1–72) clone (long linker) originally isolated in the screen, generated activities above background in combination with all three T18 fusion constructs. Again, higher activities with the T18-Rne constructs as compared to the T18-Rng fusion were measured. Apparently, SlyX and YdfR bind both, Rng as well as the Rne-NTD, with preference for the latter. In case of YfdR, the interaction surface should be localized within the first 72 residues. Possibly, interaction with Rng and Rne_FL_ might be hindered by the fused T25 domain when connected through a short linker only. Alternatively, the C-terminal portion of YdfR (residues 73–103) may shield residues required for these interactions, albeit this is not supported by AlphaFold (identifier AF-P76160-F1), which predicts an α-helical structure for this region, pointing away from the N-terminal domain. We concluded that SlyX and YdfR bind the Rne-NTD and included these proteins in subsequent analyses.

### BACTH analysis of full-length versions of the candidate proteins classifies them into three distinct groups

Except for a few cases, our screens recovered protein fragments fused to the T25 domain. For validation, it is good practice to test the prey proteins in their full-length sequences [40]. Of the 43 prey proteins (Table 2), we shortlisted 20 for further analysis. On one hand, we chose soluble proteins localizing to the cytosol (RtcB, SlyX, ThrB, WcaF, YcbZ, YdfR, YegJ) or being secreted (FliD). On the other hand, we selected inner membrane proteins that were reiteratively identified in the screens emphasizing that they are likely true hits (i.e. CorA, DacC, DcuA, GltP, MurG, PstC, RseA, YohP, YphA, YqjF). Additionally, we included the inner membrane proteins CydH and YfgM (identified once), as these proteins were recovered in their full-length versions in the BACTH screen, making novel constructions superfluous. The corresponding full-length genes were cloned in frame with *T25* between the XbaI/KpnI sites of plasmid pKT25. In the first test, we combined each T25 fusion construct with the T18-Rne construct that led to identification of that prey protein in the BACTH screen. Quantitative and/or phenotypic BACTH assays failed to confirm interaction of the DacC, DcuA, FliD, RseA and RtcB full-length protein fusions with the respective Rne bait construct. In contrast, interactions with the Rne bait constructs were detectable for all remaining candidate proteins when tested in their full-length forms (Table 2; Suppl. Table S5; Suppl. Figure S4).

Next, we wanted to gain insight into the domains of Rne that are required for the interactions with the various prey proteins. Therefore, we combined the T25-constructs encoding the positively tested full-length candidate proteins with the T18 constructs expressing various truncated versions of Rne as shown in Figure 1(A). Quantitative and/or phenotypic BACTH assays were performed to assess interactions (Figure 2, Suppl Figure S4, Suppl. Table S5). Interestingly, the proteins split into three groups with respect to interaction with the various Rne truncations: The first group comprising CorA, CydH, GltP, MurG, YfgM, YohP, YphA and YqjF exhibits a pattern reminiscent of CsrD. These proteins interact with the Rne_1–597_ and Rne_499–1061_ truncations, but not with any other variant (Figure 2(A)). Mutation of FF/AA of the MTS within the RNase E_1–597_ construct had no large effects on these interactions. The second group comprising proteins SlyX, YegJ, YdfR and ThrB produced enzyme activities above background exclusively when combined with Rne constructs comprising the catalytic domain, similar to RapZ, but with some deviations (Figure 2(B); Suppl. Table S5). Whereas YegJ and SlyX perfectly recapitulated the interaction pattern exhibited by RapZ and produced enzyme activities above background even in combination with the short Rne_1–415_ variant, this was not the case for the two other proteins. The T25-ThrB and T25-YdfR fusions generated positive BACTH signals only in combination with the Rne_1–597_ constructs. Finally, proteins WcaF and YcbZ form a third group: They interact with the Rne_1–597_ as well as the even shorter Rne_1–498_ and Rne_1–415_ variants, similar to RapZ (Figure 2(C); Suppl. Table S5). As a difference, T25-WcaF and T25-YcbZ also produced high enzyme activities when combined with the T18-Rne_499–1061_ construct. According to these results, WcaF and YcbZ contact at least two distinct sites in Rne, which apparently localize within the first 415 residues and between residues 499–586 of Rne, respectively.

### Copurification and pull-down approaches validate interaction of SlyX, YdfR and YegJ with the catalytic domain of RNase E

**Figure 3.**
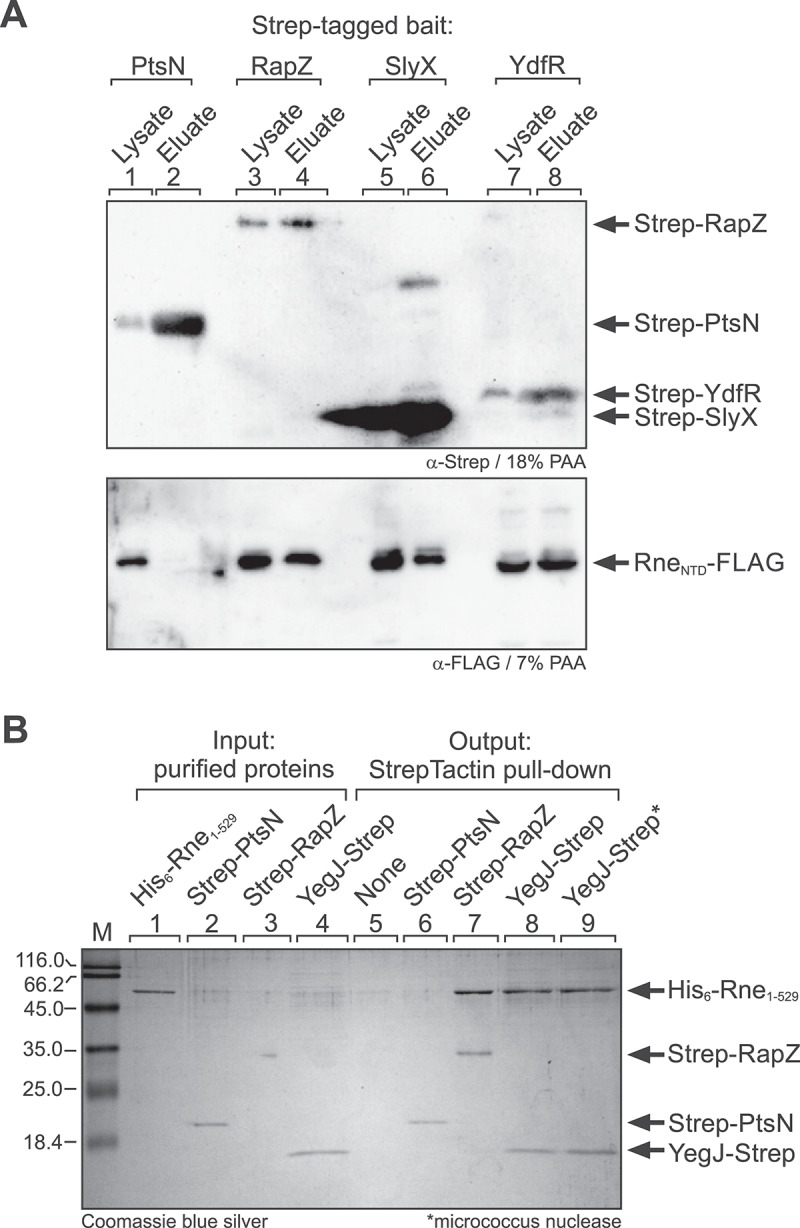
Validation of interaction of SlyX, YdfR and YegJ with the catalytic domain of Rne using copurification and pull-down approaches. (A) Ligand fishing experiment based on StrepTactin affinity chromatography indicating interaction of Strep-RapZ, Strep-SlyX and Strep-YdfR with Rne_1–598_. Strain Z899 was used, which encodes a FLAG-tagged version of Rne_1–598_ in the chromosome, and carried either plasmid pBGG217 encoding Strep-PtsN (lanes 1, 2; negative control), plasmid pBGG164 encoding Strep-RapZ (lanes 3, 4; positive control), plasmid pYG181 encoding Strep-SlyX (lanes 5, 6) or plasmid pYG182 encoding Strep-YdfR (lanes 7, 8). The transformants were grown in LB and synthesis of Strep-tagged proteins was induced with 1 mM IPTG. Lysates were prepared and subjected to column-based StrepTactin affinity chromatography. Subsequently, 10 μl of each lysate and elution fraction II were separated by SDS-PAGE and presence of the Strep-tagged bait proteins was confirmed by Western blotting using anti-Strep antiserum (top panel). Copurifying Rne_1–598_-FLAG was detected by Western blotting using anti-FLAG antiserum (bottom panel). (B) *in vitro* pull-down assays testing interaction of YegJ-Strep with the His_6_-tagged Rne_1–529_ variant. Strep-PtsN (lane 6, negative control), Strep-RapZ (lane 7, positive control), YegJ-Strep (lanes 8, 9) or no protein (lane 5, negative control) was immobilized on Strep-Tactin XT magnetic beads and subsequently incubated with His_6_-Rne_1–529_. Bound proteins were analysed by SDS-PAGE and Coomassie blue silver staining (‘output’, lanes 5–9). In lane 9, Strep-RapZ and His_6_-Rne_1–529_ preparations were treated with micrococcus nuclease prior to their copurification (indicated by an asterisk). Purified protein preparations were analysed in lanes 1–4 (‘input’). Note that the Strep-RapZ signal in lane 3 is partially quenched by a gel artefact.

Our analysis suggested that SlyX, YdfR and YegJ contact exclusively the N-terminal catalytic domain of Rne. To validate their interaction with the Rne-NTD by a complementary approach, we performed ligand fishing experiments investigating whether the Rne-NTD would co-elute with these proteins upon their affinity purification on StrepTactin columns. To this end, we expressed N-terminally Strep-tagged SlyX and YdfR from plasmids in strain Z899, which carries a FLAG-tagged variant of the Rne-NTD (residues 1–598) encoded in the chromosome. Examination of total protein extracts by SDS-PAGE/Coomassie blue staining confirmed accumulation of both recombinant proteins following induction of expression with IPTG (Suppl. Figure S5A). In previous work, we demonstrated co-purification of Rne_1–598_ with Strep-RapZ upon StrepTactin affinity chromatography, whereas the phosphotransferase protein PtsN does not co-purify with Rne [32,34], reflecting that it does not bind Rne (Figure 1(D)). Therefore, we included Strep-RapZ and Strep-PtsN as positive and negative controls, respectively. Cell extracts of the various transformants were subjected to StrepTactin column affinity chromatography and the Strep-tagged bait proteins were eluted in three fractions (Suppl. Figure S5B, C). Presence of the Strep-tagged bait proteins in the total cell extracts as well as in the elution fractions II was confirmed by Western blotting using α-Strep antiserum (Figure 3(A), top panel) and by Coomassie blue staining (Suppl. Fig S6). Western blotting using α-FLAG antiserum detected the Rne_1–598_ variant in the eluate of RapZ, but not in the eluate of PtsN, as expected. Importantly, Rne_1–598_ was also present in the eluates of SlyX and YdfR, confirming their interaction.

We failed to detect and to purify protein YegJ via an N-terminal Strep-tag. C-terminally Strep-tagged YegJ was detectable by Western blotting in strain Z899, but only weakly (Suppl. Figure S7A). Deletion of Lon protease (strain Z909) somewhat improved YegJ-strep amounts, but purification failed (Suppl. Figure S7A, B). Fortunately, we succeeded with overproduction and purification of YegJ-strep using strain BL21 (DE3), albeit several contaminating proteins were also detectable in the eluates (Suppl. Figure S7A, B). Therefore, we made use of an *in vitro* pull-down assay to confirm binding of YegJ to the Rne-NTD. Purified YegJ-Strep (Figure 3(B), lane 4) was immobilized on Strep-Tactin XT magnetic beads and co-incubated with the purified His_6_-Rne_1–529_ variant (Figure 3(B), lane 1) that comprises the catalytic domain. Following washing and elution, the eluates were analysed by SDS-PAGE/Coomassie blue silver staining. YegJ-Strep retained Rne_1–529_ on the beads, similar as Strep-RapZ, which was used as positive control (Figure 3(B), lanes 7, 8). In contrast, Rne_1–529_ could not be detected in the eluate when incubated with Strep-PtsN immobilized on the StrepTactin beads or with the beads alone (Figure 3(B), lanes 5, 6). We performed an additional experiment, in which we treated the YegJ-Strep and His_6_-Rne_1–529_ preparations with micrococcal nuclease to remove all nucleic acids prior to the pull-down assay. Nonetheless, His_6_-Rne_1–529_ remained detectable in the eluate indicating that the YegJ/Rne_1–529_ interaction involves direct contacts between both proteins (Figure 3(B), lane 9).

### Overproduction of SlyX, ThrB or YegJ shapes the transcriptome in a similar manner

We wanted to learn whether the group II prey proteins that contact exclusively the Rne-NTD (Figure 2(B)) affect its catalytic activity – either stimulating or inhibiting. These proteins could target specific transcripts to Rne like RapZ or affect Rne activity globally like RraA. We used an RNA sequencing approach to identify transcripts possibly affected by SlyX, ThrB, YdfR and YegJ. As we could not know whether these candidates are expressed under standard growth conditions, we analysed the effects of plasmid-based overproduction rather than using deletion mutants. Indeed, almost no read counts for *yegJ* and *ydfR* were obtained in our subsequent RNA-seq analyses, except in the strains overexpressing these genes. Total RNA was isolated from exponentially growing cells 30 min after induction of overexpression with IPTG. As IPTG-induction of *yegJ* overexpression results in a growth arrest (see the last results section), we additionally included non-induced cells of this transformant in the analysis. We assessed total protein extracts by SDS-PAGE/Coomassie blue staining to verify accumulation of the various proteins in the IPTG treated cultures (Suppl. Figure S8A). All proteins visibly accumulated, except YegJ-Strep. As observed before, a faint band referring to YegJ-Strep could only be detected by Western blot analysis (Suppl. Figure S8B).

**Figure 4.**
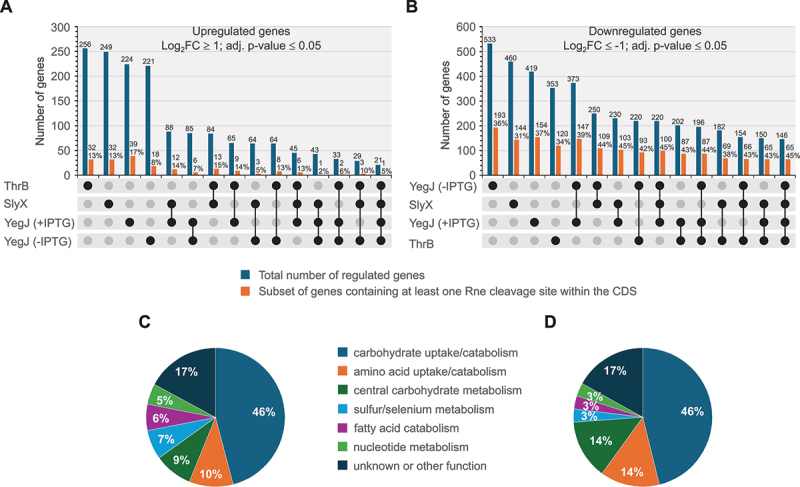
Transcriptome changes elicited by overproduction of ThrB, SlyX and YegJ. (A) Upset plot depicting the number of upregulated genes in the transcriptomes of the ThrB, SlyX and YegJ (-IPTG and +IPTG datasets) overproducers and the number of shared upregulated genes in all possible cross-comparisons (blue columns with number of upregulated genes shown above). Only genes with log2-fold changes ≥ 1 and adjusted *p*-values ≤ 0.05 were considered. Orange columns indicate the fraction of these upregulated genes that contain at least one Rne cleavage site in the CDS. The number of corresponding genes and their relative percentage proportion are depicted above. (B) Similar to (A), but as a difference the downregulated genes were analysed. (C) Functional categorization of the 146 downregulated genes shared by all four transcriptomes (last blue column in (B)). Functions were deduced from gene annotations in the ecocyc database [64]. (D) Functional categorization of the 65 downregulated genes that contain at least one Rne cleavage site and are shared by all four transcriptomes (last orange column in (B)).

To identify regulated genes in the RNAseq data sets of the various overproducers, the corresponding normalized mean reads were each compared with the reads obtained from the transformant carrying the corresponding empty vector pBGG237 (Suppl. Excel file). In each of these comparisons, the overexpressed candidate represents the most highly upregulated gene, as expected. This was also the case for *yegJ* in the corresponding non-induced cells, showing that the basal expression level of the plasmid already triggers a moderate overexpression, reflecting leakiness of repression by LacI. Upon addition of IPTG to this strain, the mean normalized read counts for *yegJ* further increased 29-fold, verifying inducibility of *yegJ* expression. Overproduction of YdfR had the smallest impact on the transcriptome: 24 genes were upregulated, and 34 genes were downregulated when considering only genes regulated with log2-fold changes ≥ 1 or ≤−1 and adjusted *p*-values ≤ 0.05. In contrast, the effects of the three other overproduced proteins on the transcriptome were roughly one order of magnitude higher. In these cases, more than 200 genes were upregulated, and even more, i.e. ~300 to 500, were downregulated, respectively, when applying the same cut-off criteria (Figure 4(A,B), blue columns 1–4). To see whether regulated transcripts are shared between data sets, cross-comparisons were performed. This analysis revealed a high overlap between the datasets obtained from the uninduced and IPTG-induced YegJ overproducers, as expected: 85 upregulated genes and 373 downregulated genes are shared by both data sets (Figure 4). Interestingly, for many genes the fold-changes were even greater in the non-induced YegJ overproducer than in the induced strain, suggesting that the deleterious effect of *yegJ* overexpression (see the last results chapter) limits the regulatory capacity of the cells (Suppl. Excel file and Suppl. Tables S9, S10). To our surprise, comparable high overlaps were observable when performing additional cross-comparisons including also the RNA-seq results obtained for SlyX and ThrB (Figure 4). Generally, the three overproducers shared more down- than up-regulated genes with each other (Figure 4, compare A and B). 21 upregulated and 146 downregulated genes were shared by all four data sets. Thus, a portion ranging from 32% (YegJ) to 39% (ThrB) of all genes that were downregulated by one candidate were also downregulated by the other two proteins.

**Figure 5.**
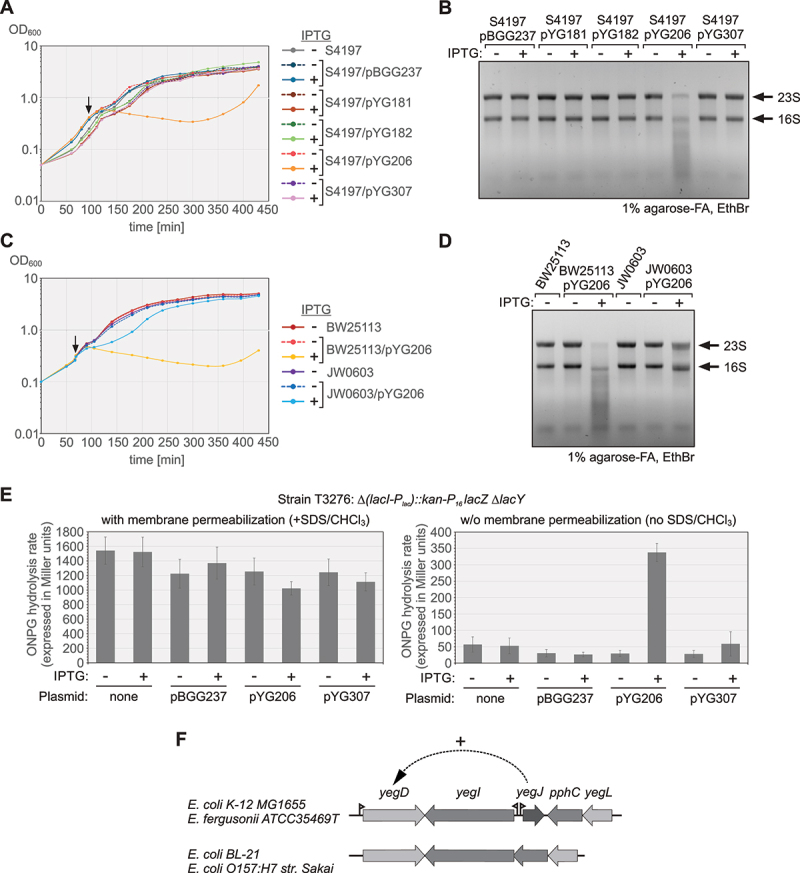
Overexpression of *yegJ* destabilizes rRNA, arrests growth and confers cell envelope leakiness. (A) Growth curves for strain S4197 and its transformants carrying the vector control pBGG237, plasmid pYG181 for overexpression of *slyX*, plasmid pYG182 for overexpression of *ydfR*, plasmid pYG206 for overexpression of *yegJ* or plasmid pYG307 for overexpression of *thrB*. The various strains were inoculated in LB to an initial OD_600_ = 0.05 and subsequently grown until cultures reached OD_600_ ~ 0.3. At this point, each culture was split into two subcultures, one of which received 1 mM IPTG for induction of plasmid-encoded gene expression, whereas the second culture received H_2_0 as mock. Growth was continued and the OD_600_ values were recorded at regular time intervals. The arrow indicates the time of addition of IPTG or H_2_0 to the cultures of S4197/pYG206. (B) 23S and 16S rRNA profiles of the various transformants tested in (A), following 30 min of induction with IPTG or addition of H_2_O as mock. Total RNAs were extracted from samples that were harvested from the various transformants 30 min after addition of 1 mM IPTG or H_2_O as mock. Total RNAs (3 μg each) were separated by denaturing formaldehyde agarose gel electrophoresis and gels were stained with ethidium bromide for visualization of the rRNAs. (C) Growth curves of strains BW25113 (*wild-type*) and its isogenic *Δrna* mutant JW0603 as well as their transformants carrying plasmid pYG206 for overexpression of *yegJ*. The experimental setup was as described for (A) but as a difference the cultures were initially inoculated to an OD_600_ = 0.1. (D) 23S and 16S rRNA profiles of the strains and transformants tested in (C). The experimental setup was as described in (B), but as a difference, samples for total RNA extraction were harvested 1 h after induction with IPTG or addition of H_2_O as mock. The arrow indicates the time of addition of IPTG or H_2_0 to the cultures of BW25113/pYG206 and JW0603/pYG206. (E) Envelope permeability assay using the *ΔlacY* strain T3276, which expresses *lacZ* constitutively. Strain T3276 and its transformants carrying plasmid pBGG237 (vector control), plasmid pYG206 (*yegJ*) or plasmid pYG307 (*thrB*) were grown as described under (a). Samples were harvested 30 min after addition of IPTG or H_2_O and cell suspensions were subjected to β-galactosidase activity assays following a membrane permeabilization step (left diagram) or omitting the latter (right diagram). The measured ONPG hydrolysis rates are expressed in Miller units and presented as mean ± standard deviation. Replicate numbers are *n* = 3 for the empty strain T3276 and *n* = 4 for the transformants carrying plasmids. A paired two-tailed t-test indicates that the ONPG hydrolysis rate of T3276/pYG206 (+IPTG) is significantly higher than the hydrolysis rate of the vector control T3276/pBGG237 (+IPTG) when membrane permeabilization was omitted (right panel); *p*-value = 5,70966e-07. (F) Gene synteny of the *yegD-yegL* locus in *Escherichia fergusonii* and various *E. coli* strains. Overexpression of *yegJ* activates expression of *yegD* in *E. coli* K-12 (Suppl. Table S9).

To identify genes that are specifically regulated by a particular prey but not by any other of the three remaining candidate proteins, we applied a rigorous filtering to the RNA-seq data. First, we filtered all genes that are regulated by a particular prey with log2 fold change ≥ 1.5 or ≤−1.5 (adjusted *p*-value ≤ 0.05). Subsequently, we eliminated from these lists all genes that are also regulated by at least one of the three other overproducers in the same direction with a log2 fold change ≥ 0.5 or ≤−0.5 and an adjusted *p*-value ≤ 0.1. As result we obtained lists of genes that are specifically up- or down-regulated by each of the four prey proteins, respectively (Suppl. Tables S6-S10). For instance, only SlyX overexpression upregulated the SOS response genes *umuD* and *sulA*, and down-regulated the co-transcribed *rsxG-rsxE-nth* genes, which are involved in reduction of the iron-sulphur cluster of SoxR (Suppl. Table S6). Similarly, down-regulation of *ptsG* and *cpxP* encoding the glucose transporter and a regulatory protein of the Cpx stress response, respectively, occurred exclusively in the ThrB overproducing strain (Suppl. Table S8). Moreover, it became evident that moderate overproduction of YegJ (−IPTG data set) causes strong upregulation of *yegD*, a gene encoding presumably an HSP70 chaperone that is separated from *yegJ* by only 2159 bp (Suppl. Table S9; Figure 5(F)). Taken together, the various overproductions had a surprisingly similar effect on the transcriptome, except for YdfR affecting only few transcripts, and relatively few genes appear to be specifically regulated by a particular candidate.

### The genes downregulated by SlyX, ThrB or YegJ are enriched with Rne cleavage sites

Previous work has shown that protein overproduction *per se* provides a burden to *E. coli* and may change the levels of mRNAs and proteins and induce stress pathways [66]. In particular, ‘heat shock genes’ encoding chaperones and proteases are highly upregulated in such overproducers, whereas the levels of certain ribosomal proteins (RpsF, RplI) are decreased [67] - effects that are also visible in our data: In all RNA-seq datasets, genes for chaperones such as *ibpA*, *ibpB*, *dnaK*, *dnaJ*, *clpB* and for the proteases *hslV* and *hslU* are up-regulated, while *rpsF* and *rpslI* are down-regulated (Suppl. Excel file). As an exception, this is only partially observed or less pronounced in the uninduced (−IPTG) YegJ overproducer, which may reflect the only moderate overproduction. In conclusion, many of the upregulations that are shared among the transformants overproducing SlyX, ThrB and YegJ may have resulted from the burden caused by protein overproduction.

Functional categorization of the 146 downregulated genes that were shared by the transcriptomes of the SlyX, YegJ and ThrB overproducers (Figure 4(B)) revealed a strong enrichment of metabolic genes. These genes refer mostly to functions for uptake and catabolism of carbohydrates and amino acids and include genes of the central carbohydrate metabolism such as *mdh, sucA*, *acs, fumA* and *sdhCDAB* (Figure 4(C)). To a lesser extent, genes involved in sulphur or selenium metabolism, fatty acid degradation, and nucleotide metabolism are also represented. Previous work found that the levels of most TCA cycle enzymes increase upon protein overproduction in *E. coli* [67,68], providing no obvious explanation for their collective repression in the various RNA-seq experiments. To learn whether Rne could be involved, we made use of a recently published transcriptome-wide atlas of Rne cleavage sites in *E. coli* [6]. We filtered the 7490 Rne cleavage sites mapped in that work to keep those that are located within the CDS of annotated genes, considering that the effects of Rne cleavages within untranslated regions on gene expression are hard to predict. The filtering resulted in a list of 869 genes collectively carrying 4609 Rne cleavage sites. That is, out of the 4247 genes, 869 genes possess at least one Rne cleavage site within the CDS, referring to 20.5% of all genes in total (Suppl. Excel file). Next, we determined how many of the genes that were up- or downregulated in the individual RNA-seq experiments and their cross-comparisons contained at least one Rne cleavage site (Figure 4(A,B), orange columns). Interestingly, among the upregulated genes, the fraction of genes containing at least one Rne cleavage site is well below the 20.5% value in the individual datasets and cross-comparisons (Figure 4(A)). The highest proportion, referring to 17%, is detected in the YegJ (−IPTG) data set. This value decreases to 8% in the YegJ (+IPTG) dataset, suggesting that induction of YegJ selectively eliminated mRNAs that are cleaved by Rne. In contrast, the proportion of genes containing at least one Rne cleavage site is much higher among the downregulated genes, ranging from 31% (SlyX) to 37% (YegJ +IPTG) for the three prey proteins (Figure 4(B)). This proportion increases further in the various cross-comparisons, reaching a maximum of 45% (=65 genes) among the 146 downregulated genes that are shared by all four transcriptome analyses (Figure 4(B)). Examining the observed proportions for the probability of obtaining them under the null hypothesis of independent draws from the genomic background, where 20.5% of the genes harbour an RNase E (Rne) cleavage site, confirms that these genes are significantly enriched among down-regulated genes across all overproducers analysed (Fisher’s exact test, Benjamini-Hochberg adjusted *p*-value < 0.01, odds ratios 1.9–2.6), whereas such genes were significantly depleted among up-regulated genes for most overproducers (Fisher’s exact test, adjusted *p* < 0.01, odds ratios 0.3–0.6). The only overproducer deviating from this pattern is YegJ (+IPTG), which exhibits no significant association between Rne cleavage site presence and gene up-regulation (Fisher’s exact test, adjusted *p* = 0.3, odds ratio 0.8) (Suppl. Figure S9).

In respect to the encoded function no major difference between the 146 genes downregulated by all candidate proteins and the fraction of 65 genes that carry Rne cleavage sites was apparent, except that the prevalence of genes involved in central carbohydrate and amino acid metabolisms was somewhat increased in the latter group (cf Figure 4(C,D)). Taken together, genes with Rne cleavage sites appear to be selectively depleted from the genes that are upregulated by the candidate proteins. In contrast, genes carrying Rne cleavage sites appear to be enriched among the downregulated genes, and this enrichment increases further for those genes that are shared between datasets (Figure 4(B)). Thus, overproduction of SlyX, ThrB or YegJ correlates with increased Rne activity. However, it cannot be ruled out yet that the changed RNA decay patterns resulted from proteotoxic stress caused by overproduction, rather than from binding of the candidate proteins to Rne.

### Overproduction of YegJ compromises envelope integrity and destabilizes rRNA

We observed that induction of *yegJ* expression with IPTG confers a strong growth defect on the *E. coli* ‘*wild-type*’ strain S4197, whereas this could not be observed for the other transformants carrying isogenic constructs for overexpression of *slyX*, *ydfR*, *thrB* or the empty vector (Figure 5(A)). We isolated total RNAs from the various transformants 30 min after addition of IPTG or H_2_O as mock control. To check for integrity, the total RNAs were separated by denaturing formaldehyde agarose gel electrophoresis followed by ethidium bromide staining. Interestingly, massive degradation of the 23S and 16S rRNA species became visible in the sample extracted from the IPTG-induced transformant S4197/pYG206 overproducing YegJ, but not in any other sample (Figure 5(B)). This strong rRNA destabilization could explain the growth defect of the corresponding transformant and our unsuccessful attempts to purify Strep-YegJ from *E. coli*-K12 strains.

RNase I encoded by gene *rna* is a non-specific endoribonuclease that localizes in the periplasm. It has been reported that increased permeability of the membrane, as resulting from its damage, may provide access of RNase I to the cytoplasm, which may then degrade rRNAs non-specifically [69]. Interestingly, the RNA-seq analysis of the YegJ-overproducer (+IPTG) revealed the strong upregulation of nine genes that are positively controlled by response regulator CpxR of the CpxAR envelope stress response system (Suppl. Table S10), which responds primarily to alterations of the cytoplasmic membrane [70]. Additionally, the expression of two genes (*pspB* and *pspC*) of the phage shock protein system, which likewise responds to extracytoplasmic stress, is increased. Notably, these upregulations are not observable in any of the other RNA-seq analyses. It appeared feasible that YegJ overproduction causes membrane leakage, thereby possibly granting RNase I access to the cytoplasmic rRNAs. To test this idea, we introduced the *yegJ* overexpression plasmid pYG206 into the *rna* deletion mutant JW0603 of the Keio strain collection and included the isogenic *wild-type* strain BW25113 for comparison. Once again, dramatic rRNA destabilization accompanied with a severe growth defect was observable upon IPTG-induction of *yegJ* expression in the *wild-type* BW25113, demonstrating that this phenomenon is not restricted to a particular *E. coli*-K12 strain lineage (Figure 5(C,D)). Indeed, overproduction of YegJ (+IPTG) caused a less severe growth defect in the *Δrna* deletion mutant JW0603 as compared to the *wild-type* BW25113 (Figure 5(C)). In agreement, 23S and 16S rRNA species became stabilized in the *Δrna* mutant (Figure 5(D)). Nonetheless, a remarkable growth retardation accompanied by some residual rRNA destabilization, remained even detectable in the *Δrna* mutant overproducing YegJ (Figure 5(C,D)).

Finally, we wanted to confirm that YegJ overexpression compromises the inner membrane as suggested by the RNA-seq data indicating upregulation of cognate stress response systems. To this end, we adopted a previously established envelope integrity assay [59]. This assay measures the availability of externally provided ONPG as substrate for cytoplasmic β-galactosidase (LacZ) in *E. coli* mutants lacking permease LacY, known to transport ONPG. Usually, such mutants show a low ONPG hydrolysis rate because LacZ has no access to its substrate in the exterior. However, conditions that cause leakiness of the cytoplasmic membrane allow ONPG to pass, resulting in increased ONPG hydrolysis rates. For this test, we introduced plasmids pYG206 (overexpressing *yegJ*), as well as plasmids pBGG237 (empty vector) and pYG307 (overexpressing *thrB*) as controls, into *E. coli* strain T3276, which lacks *lacY* and transcribes the chromosomal *lacZ* gene constitutively from the synthetic *P*_*16*_ promoter. The transformants as well as the plasmid-less strain, which was included as additional control, were grown to exponential phase and supplemented with of IPTG or H_2_O. Samples were harvested 30 min later, i.e. before cell density of the culture overproducing YegJ started to decline (Suppl. Figure S10). The cells were subjected to β-galactosidase assays using ONPG as substrate and either including or omitting prior membrane permeabilization with SDS and CHCl_3_. The SDS/CHCl_3_ treatment allows for unrestricted diffusion of ONPG into the cell and the results prove that all transformants produced comparable β-galactosidase activities i.e. LacZ levels (Figure 5(E), left). However, when omitting the membrane permeabilization step, only low background ONPG hydrolysis rates were measured, except for the transformant overproducing YegJ (+IPTG). In this case, a ~ 10-fold higher activity was produced indicating membrane leakiness (Figure 5(E), right). Taken together, these data show that high YegJ levels damage the cytoplasmic membrane, allowing RNase I to enter the cytoplasm and to degrade rRNAs. However, additional yet unknown RNase(s) or processes also contribute to rRNA destabilization.

## Discussion

In this work, we used a BACTH screen to identify proteins binding to Rne bait constructs lacking sequences from the C-terminus. The screen recovered fragments of 43 prey proteins (Suppl. Table S3), of which 15 were validated to bind also as full-length proteins to Rne, as tested by BACTH (Table 2, Figure 2). These 15 hits split into three groups (Figure 2). Group I proteins and also CsrD yielded only positive BACTH signals when combined with Rne constructs that included residues 499–586, also comprising the MTS (residues 568–582), suggesting that this region contains the binding site (Figure 2(A)). In contrast to groups II and III, group I proteins are all membrane-bound and at least some of them may use a TMD to bind the MTS region. Group II proteins likewise interact with Rne_1–597_ but not with Rne_499–1061_, indicating that the interaction surface is located within the catalytic domain of Rne (Figure 2(B); Figure 3). This is confirmed for SlyX and YegJ, which also bind Rne_1–498_ (Figure 2(B)). In contrast, ThrB and YdfR do not bind Rne_1–498_. Perhaps, their binding sites overlap with residues 498/499 and are consequently disrupted in the Rne_1–498_ and Rne_499–1061_ constructs. Finally, proteins WcaF and YcbZ form a third group, apparently contacting two distinct sites in Rne, one in the large domain and another within the MTS-containing segment of Rne (Figure 2(C)). Thus, group II and group III proteins could potentially regulate activity of the Rne catalytic domain like phage T4 protein Srd or present transcripts for regulated turnover like RapZ, whereas group I proteins likely act by different means.

Interestingly, all group I proteins localize in the IM. The only exception is MurG, which is associated with the IM by other means [71]. Among all screening hits, 32 of the 46 (=70%) different fragments include at least one annotated TMD (Table 2), indicating an enrichment of fragments localizing to the IM [72]. Notably, several hits from the BACTH screen add little more than a single TMD to the T25 domain. This applies to YqjF (Figure 2(A)), and the inserts of several uncharacterized prey clones (i.e. *baeS*, *dacA*, *narQ*, *pstC*, *ttdT*, *yfcC*, *yjhF*; Suppl. Table S3). CydH and YohP (Figure 2(A)), both of which were recovered in full-length in the screen, are small proteins that consist almost entirely of a single TMD [73]. The obvious conclusion is that the encoded TMD is responsible for the positive BACTH signals in these cases and perhaps this applies to more if not all preys in group I. A possible binding surface for the hydrophobic TMD α-helices [74] is offered by the hydrophobic face of the amphipathic α-helix of the Rne MTS [61]. Interestingly, the pole-to-pole oscillating protein MinD was recently reported to bind the Rne-MTS, demonstrating that this site can be contacted by other proteins with functional consequences for Rne localization and activity [75]. The FF/AA (F574A F575A) substitutions within the MTS, previously suggested to weaken Rne membrane localization [21,61], had no major effect on interaction with the various preys (Figure 2), which may at first glance contradict the hypothesis that group I proteins bind the Rne-MTS through their TMDs. However, the MTS aligns 9 hydrophobic residues on one side of its amphipathic α-helix and two exchanges with likewise hydrophobic Ala residues might be insufficient to disrupt membrane binding and interaction as also indicated by a recent study [22]. Instead, data presented by Troyer at al. suggest that substitutions with negatively charged Glu residues should be used to diminish membrane binding of Rne. The BACTH system has been extensively used in the past to study and characterize interaction between membrane proteins [76,77]. However, unspecific weak BACTH signals are more often observed for membrane proteins than for soluble proteins, likely due to overcrowding of the membrane with the candidate proteins, driving their unspecific association in the two-dimensional space [40]. Albeit group I proteins generated robust BACTH signals with the Rne constructs containing the MTS (Figure 2(A)) and we identified some of the membrane proteins or even domains (and not others) reiteratively in the screens (Table 2), we currently cannot exclude that some of these candidates, in particular those generating weaker BACTH signals such as YqjF and YohP (Figure 2(A)), are false-positive isolates. Our attempts to confirm the CsrD-Rne interaction by a ligand fishing approach failed, suggesting that this technique is less amenable to detect interaction between membrane-bound proteins. Likely, orthogonal methods such as Förster resonance energy transfer (FRET) or pull-down following protein cross-linking are required to validate group I proteins as interaction partners for Rne.

Group II and group III proteins, which bind to the catalytic Rne domain, localize in the cytoplasm. To get insight into the physiological roles of these interactions, we performed RNA-seq analyses of strains overproducing the group II proteins and observed that many regulated genes are shared between the strains overproducing SlyX, ThrB or YegJ (Figure 4(A,B)). Interestingly, 45% of the genes collectively down-regulated by these candidates contain Rne cleavage sites (Figure 4(B)), whereas this applies to only 20.5% when considering all *E. coli* genes that were analysed in our RNA-seq experiments. Vice versa, the upregulated genes are depleted from Rne cleavage sites (Figure 4(A)), suggesting that cleavage by Rne prevented the accumulation of transcripts. Apparently, overproduction of these three prey proteins correlates with increased Rne activity in the cell. However, it remains unclear whether these effects are direct and caused by binding of the candidate proteins, which could stimulate Rne activity or delocalize it from its sites at the membrane, granting it access to otherwise protected transcripts. It is also possible that these altered Rne decay patterns arose indirectly from proteotoxic stress associated with protein overproduction [66]. Overproduction of a protein that causes proteotoxic stress but does not bind Rne could provide insight. In any case, these shared global effects on Rne activity are most likely without physiological relevance as they may only occur upon strong overproduction and not at normal expression levels of the proteins. The physiologically relevant targets, possibly regulated by a mechanism involving Rne, are likely present in the lists reporting the genes that are specifically regulated by these proteins (Suppl. Table S6, S8, S9). Future work may address whether these expression changes depend on functional RNase E.

Except for the homoserine kinase ThrB, which catalyzes a step in threonine biosynthesis and acetyltransferase WcaF, which is involved in colanic acid synthesis, little is known about the roles of the four remaining group II and group III proteins. SlyX is encoded adjacently and in opposite direction to SlyD, which is a peptidyl prolyl cis/trans-isomerase required for phage φX174-induced cell lysis [78]. Interestingly, two genes of the SOS response, *umuD* and *sulA*, are specifically upregulated in the SlyX overproducer (Suppl. Table S6). Considering that the SOS response triggers the lytic life cycle of temperate phages, SlyX could have a role for phage propagation. Gene *ydfR* is part of the Qin prophage and co-transcribed with three additional genes of unknown function. Our RNA-seq analysis suggests that this gene cluster is not expressed under standard growth conditions, but data available at the *E. coli* Gene Expression Database (https://genexpdb.okstate.edu/) indicate upregulation of these genes under cold stress. Moreover, a genetic screen identified *ydfR* (and also *yegJ*, see below) as being important for cell envelope integrity [79], also suggesting expression under particular stress conditions. Overexpression of *ydfR* had only a limited impact on the transcriptome and except upregulation of *ascFB* (Suppl. Table S7), these changes also occurred in other overproducers (Suppl. Excel file). Interestingly, we measured increased nucleic acid concentrations of 328.9 ng/μl (≡ 586.8 ng/μg protein) in the Strep-YdfR preparation obtained by StrepTactin affinity chromatography, as determined by spectrophotometric measurement of the nucleic acid fraction extracted from the eluate (Suppl. Figure S5B, C). Similar high nucleic acids concentrations of ≥ 100 ng/μl are typically also observed in Strep-RapZ eluates reflecting co-purification of GlmY/GlmZ [32]. In contrast, ≤11 ng/μl nucleic acids were measured in the Strep-SlyX and Strep-PtsN eluates, respectively (Suppl. Figure S5B, C). An independent experiment confirmed pull-down of nucleic acids by Strep-YdfR (Suppl. Figure S11). Analysis of the nucleic acids in the Strep-YdfR eluate by agarose gel electrophoresis revealed ethidium-bromide stainable material that resisted prior digestion with DNase I, indicating it is RNA (Suppl. Figure S12). Hence, YdfR could be an RNA-binding protein, albeit we cannot exclude that a contaminating protein was responsible for RNA retention in the YdfR elution fraction (Suppl. Figure S11B).

Finally, protein YegJ represents the top hit in our BACTH screen, reflecting that it was 48× identified across various screens using different Rne bait constructs (Suppl. Table S3). The BACTH signals and pull-down results suggest that YegJ binds Rne directly (Figures 2(B) and 3B). Following IPTG induction of plasmid encoded *yegJ* expression, we observed membrane leakiness (Figure 5(E)) and massive degradation of rRNA (Figure 5(A,B)) likely responsible for the concomitant growth arrest. However, absence of periplasmic RNase I largely rescued growth and rRNA stability, albeit not completely (Figure 5(C,D)). These results indicate that overproduction of YegJ causes membrane leakage, allowing RNase I to access the cytoplasm and to degrade rRNA. Membrane damage is also reflected in our RNA-seq results, revealing that the Cpx and Psp envelope stress response systems are activated in the IPTG-induced YegJ overproducer (Suppl. Table S10). The residual rRNA destabilization that remains in the *Δrna* mutant might be caused by Rne, which is known to initiate rRNA degradation in stress and rRNA quality control processes [80]. Perhaps YegJ stimulates this latter process by binding Rne. Interestingly, gene *yegJ* is only present in *E. coli* K strains but absent in other *E. coli* lineages such as *E. coli* B strains [81]. In *E. coli* K strains, *yegJ* is located between genes *pphC* (*yegK*) and *yegI* and reads in their opposite direction (Figure 5(F)). In contrast, *pphC* and *yegI* are consecutive and *yegJ* is lacking in other *E. coli* strains. Recent studies have shown that YegI is a membrane bound eukaryotic-like Ser/Thr kinase and capable of autophosphorylation on serine residues [82]. PphC in turn is a PP2C-like Ser/Thr phosphatase and dephosphorylates YegI, suggesting that PphC and YegI act together in a common pathway [81]. It is tempting to speculate that YegJ acts in the same pathway, which is also supported by our observation that expression of *yegD*, which is encoded immediately downstream of *yegI*, is strongly increased upon overexpression of *yegJ* (Figure 5(F); Suppl. Table S9). Our RNA-seq analysis shows that *yegJ* and its neighbouring genes are not or only weakly expressed during standard growth, confirming previous work that also failed to define any condition that promotes expression of *yegI* and *pphC* [81,82]. Interestingly, data available at the *E. coli* Gene Expression Database (https://genexpdb.okstate.edu/) suggest accumulation of *yegD* transcripts upon recovery from stationary growth phase. Future work must address whether this latter effect is mediated by YegJ, and clarify the role of the YegJ-Rne interaction in this regulatory network.

BACTH assays and screens come with limitations [40], which also apply to our screen and therefore it was likely not saturated. For instance, proteins CsrD, MinD and RapZ, which are known or shown here to bind Rne_1–597_ ([34,83]; Figure 1), were not identified in the screen. Previous work showed that removal of few amino acids from either the N- or C-terminus of RapZ abolishes its interaction with Rne as detectable by BACTH [34]. However, the average length of the 61 prey inserts is 488 bp (Suppl. Table S3) and only four out of them exceed 855 bp, the length of the *rapZ* orf (Suppl. Figure S13). Thus, insert length was likely a limiting factor, impeding the recovery of proteins that require their entirety for proper oligomerization. It also appears possible that certain inserts were present at an above-average rate in the BACTH libraries, as we reiteratively recovered identical inserts for a particular prey protein. Only in two cases, different inserts were identified (Table 2). It is possible that this bias resulted from low gene coverage in the BACTH library. On the other hand, a second *dacA* fragment and novel fragments were identified when a BstZ17I-digested BACTH library was used (Suppl. Table S3), arguing that overrepresentation of *yegJ* fragments was a limitation. If so, it is possible that similar screens in the future will discover even more Rne binding partners in *E. coli*.

Additional topology-dependent limitations apply to BACTH [40], which possibly also impacted our results with the full-length sequences of the candidates recovered in our screen (Table 2). To obtain a positive BACTH signal, the CyaA domain fused to the candidate proteins must localize in the cytoplasm. However, the N-termini of the membrane proteins DacC and DcuA likely localize in the periplasm [84,85], which would make the fused T25 domain unavailable for interaction with cytoplasmic T18-Rne, explaining the negative BACTH signals (Table 2). Consistently, all other full-length membrane proteins that tested positively by BACTH (Table 2), are known or predicted to have the N-terminus in the cytoplasm [35,86–90]. The only deviations are the small proteins YohP and CydH (Figure 2(A)), for which a dual membrane orientation was reported [73]. In addition to constraints by topology, the fusion to the CyaA domain may also impair folding, stability or simply prevent interaction of the candidate with its partner by sterical hindrance, yielding false-negative results. Taking these limitations into account, it appears still possible that those candidates that failed to generate a positive BACTH signal when tested in full-length (Table 2), bind Rne through the domains identified in the screen.

In summary, a BACTH screen for proteins binding the Rne N-terminus in *E. coli* retrieved fragments of 43 proteins, many of which localize to the cytoplasmic membrane (Table 2). 15 of these candidates, which were selected for further evaluation, also generate BACTH signals with Rne when assessed in full-length. BACTH assays with truncated Rne variants classifies them into three groups regarding the contacted domains (Figure 2). Group II comprises the cytoplasmic proteins SlyX, ThrB, YdfR and YegJ, which according to BACTH bind the catalytic domain of Rne (Figure 2(B)), and pull-down approaches confirm this interaction for three of them (Figure 3). RNA sequencing analysis of strains that overexpress group II proteins identified specifically regulated genes in each case (Supplementary Tables S6–S10) but also revealed common effects on transcripts containing Rne cleavage sites, suggesting enhanced RNA degradation (Figure 4). Whether the latter results from binding of the group II proteins to Rne or from stress associated with their overproduction is currently unclear. One interesting candidate is protein YegJ, which binds the Rne catalytic domain directly (Figure 3(B)) and leads to rRNA destabilization accompanied by a growth arrest when overproduced (Figure 5(A,B)). This phenotype results from envelope leakiness granting periplasmic RNase I and perhaps also Rne access to cytoplasmic RNAs (Figure 5(C-E)). Finally, we show that the cytoplasmic membrane proteins including protein CsrD, a known regulator of sRNA decay, form an own group and generate positive BACTH signals when combined with Rne constructs comprising the MTS (Figure 2(A)). Whether these interactions are specific or driven by increased concentrations of the BACTH partner proteins at the membrane remains to be clarified.

## Supplementary Material

Supplementary Material Goepel et al 2026_revised_clean copy.docx

## Data Availability

The raw RNA sequencing data that support the findings of this study are openly available in the European Nucleotide archive (ENA) at https://www.ebi.ac.uk/ena under the accession number PRJEB108935. The authors confirm that all other data supporting the findings of this study are available within the article and its supplementary materials.
