# Supplementary material for: Identification of proteins interacting with the N-terminal half of endoribonuclease RNase E in Escherichia coli
Source: RNA Biol. 2026 May 21;23(1):1–28. doi: 10.1080/15476286.2026.2678028 (PMC13232886; doi:10.1080/15476286.2026.2678028)
Supplement: Supplementary Material Goepel et al 2026_revised_clean copy.docx [file KRNB_A_2678028_SM2798.docx]

**SUPPLEMENTARY MATERIAL**

**for**

**Identification of proteins interacting with the N-terminal half of endoribonuclease RNase E in *Escherichia coli***

Yvonne Göpel^1,2^, Svetlana Durica-Mitic^1,2,3^, Solomiia Boyko ^1,2,8^, Przemyslaw Dudys^1,2^, Fabian Amman^4,5,6^, Karin Schnetz^7^ and Boris Görke^1,2*^

^1^Max Perutz Labs, Vienna BioCenter, 1030 Vienna, Austria

^2^University of Vienna, Vienna, Austria

^3^Vienna BioCenter PhD Program, a Doctoral School of the University of Vienna and the Medical University of Vienna, Vienna 1030, Austria

^4^Center for Anatomy and Cell Biology, Medical University of Vienna, 1090 Vienna, Austria.

^5^Institute of Theoretical Biochemistry, University of Vienna, 1090 Vienna, Austria.

^6^Ludwig Boltzmann Institute for Science Outreach and Pandemic Preparedness at the Medical University of Vienna, Spitalgasse 23, 1090 Vienna, Austria

^7^Institute for Genetics, University of Cologne, Zülpicher Str. 47a, 50674 Cologne, Germany.

Current affiliations:

^8^Department of Physiology and Biophysics, Case Western Reserve University, Cleveland, Ohio, USA

^*^To whom correspondence should be addressed: boris.goerke@univie.ac.at

**Content:**

Supplementary Figures S1-S12…………………………………………………………………3

Supplementary TABLES S1-S10………………………………………………………………….16

SUPPLEMENTARY RESULTS…………………………………………………………………………28

SUPPLEMENTARY REFERENCES…………………………………………………………………..30

**Supplementary Figures**


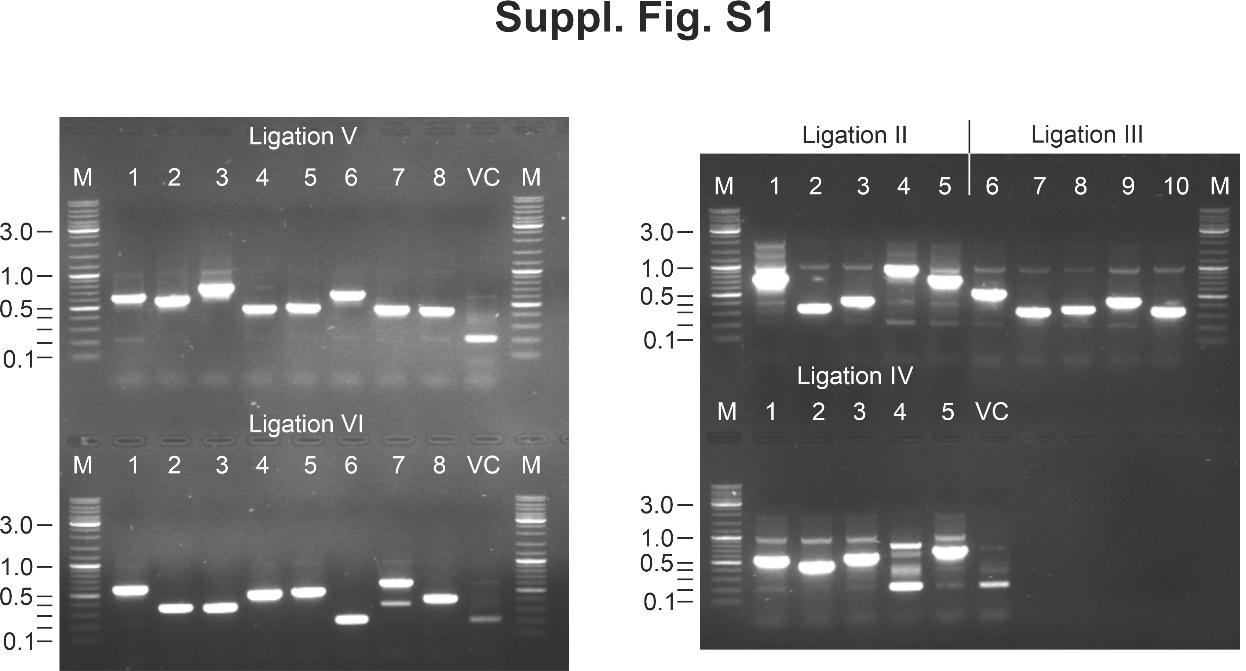


**Suppl. Fig. S1.** **PCR analysis of individual recombinants obtained from shotgun cloning of genomic DNA fragments into plasmid pKT25.** Agarose gel electrophoresis analysis of colony PCR products using individual DH5α transformants obtained from the shotgun cloning as templates. Primers BG646 and BG647 were used. Roman numbers indicate independent ligations and transformations. VC: control PCR with empty plasmid pKT25 as template, which generates a 198 bp fragment. Clones VI/7 and IV/4 likely carried a mixture of distinct plasmids.


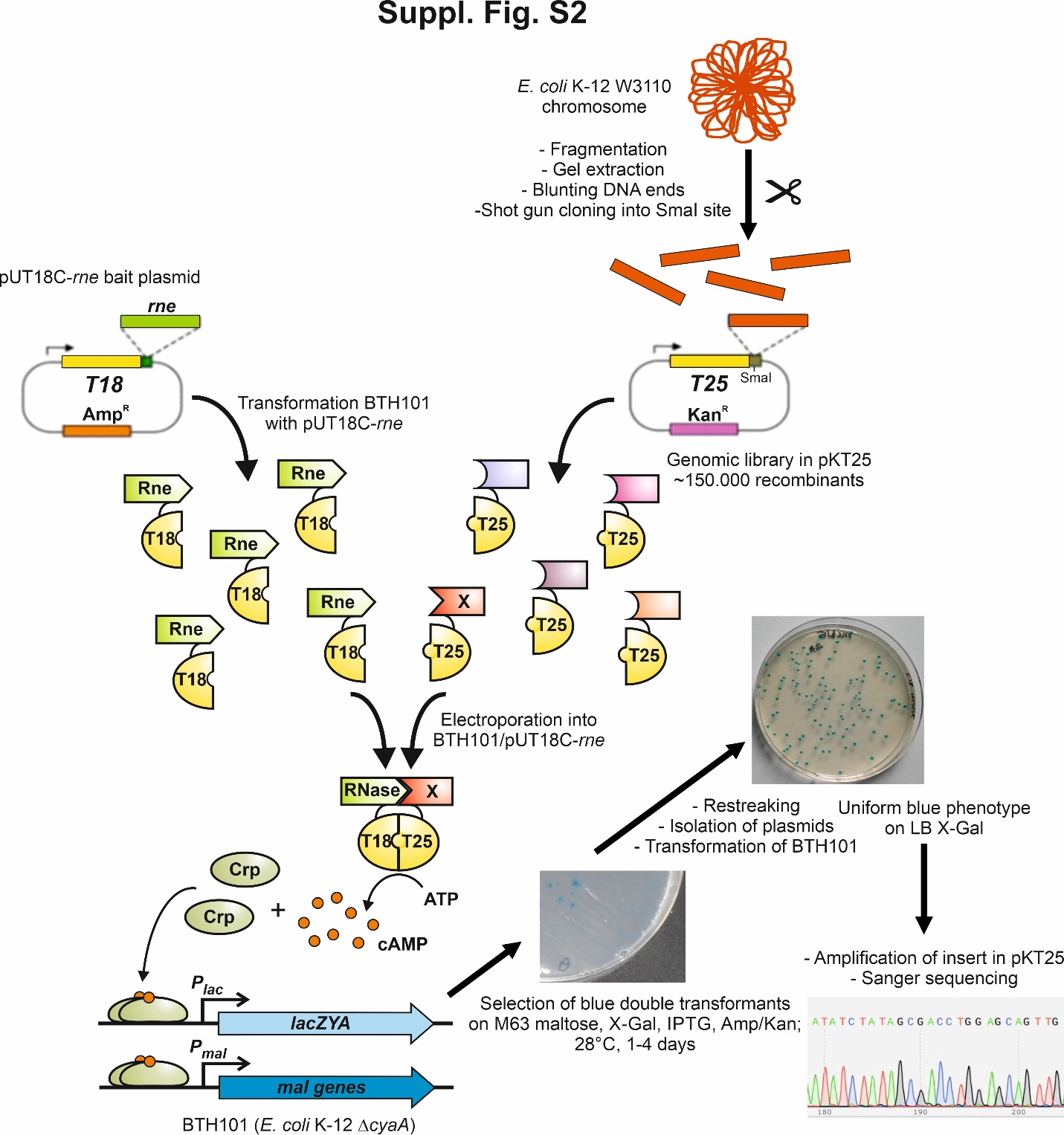


**Suppl. Fig. S2. Flowchart illustrating the major steps of genomic library preparation in the BACTH plasmid pKT25 and the principle of the double selection screen for finding proteins interacting with the T18-Rne baits.** See main manuscript for further explanations.

**
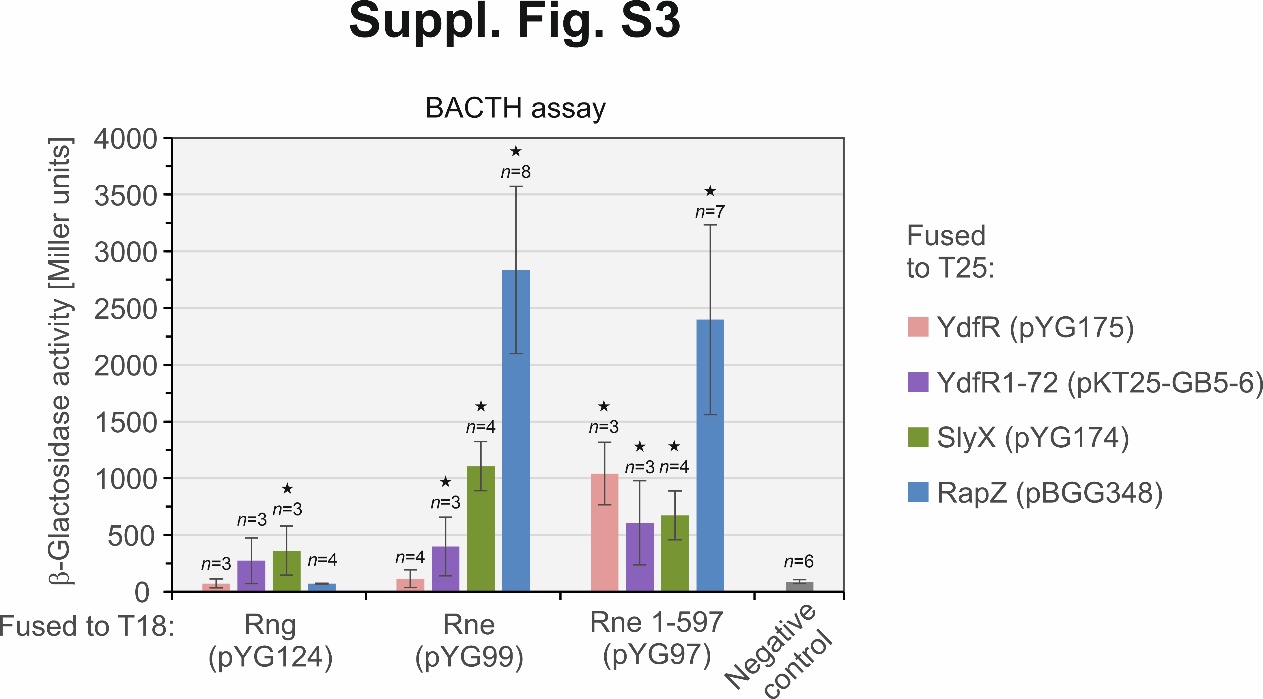
**

**Suppl. Fig. S3. Quantitative BACTH assay to assess interaction of the full-length variants of YdfR and SlyX fused to T25 with the T18-Rng, T18-Rne_FL_ and T18-Rne_1-597_ variants.** β-Galactosidase activities produced by double transformants of strain BTH101 carrying the plasmids indicated in parentheses grown to stationary phase at 28°C. The T25-YdfR (1-72) clone from the screen and T25-RapZ were included for comparison. Negative control: BTH101 pKT25/pUT18C. The β‐galactosidase activities are presented as mean ± SD and replicate numbers (*n*) are indicated. The measurements were analyzed by paired two-tailed t-test and activities significantly higher than the negative control are labeled with asterisks (*p*<0.05).


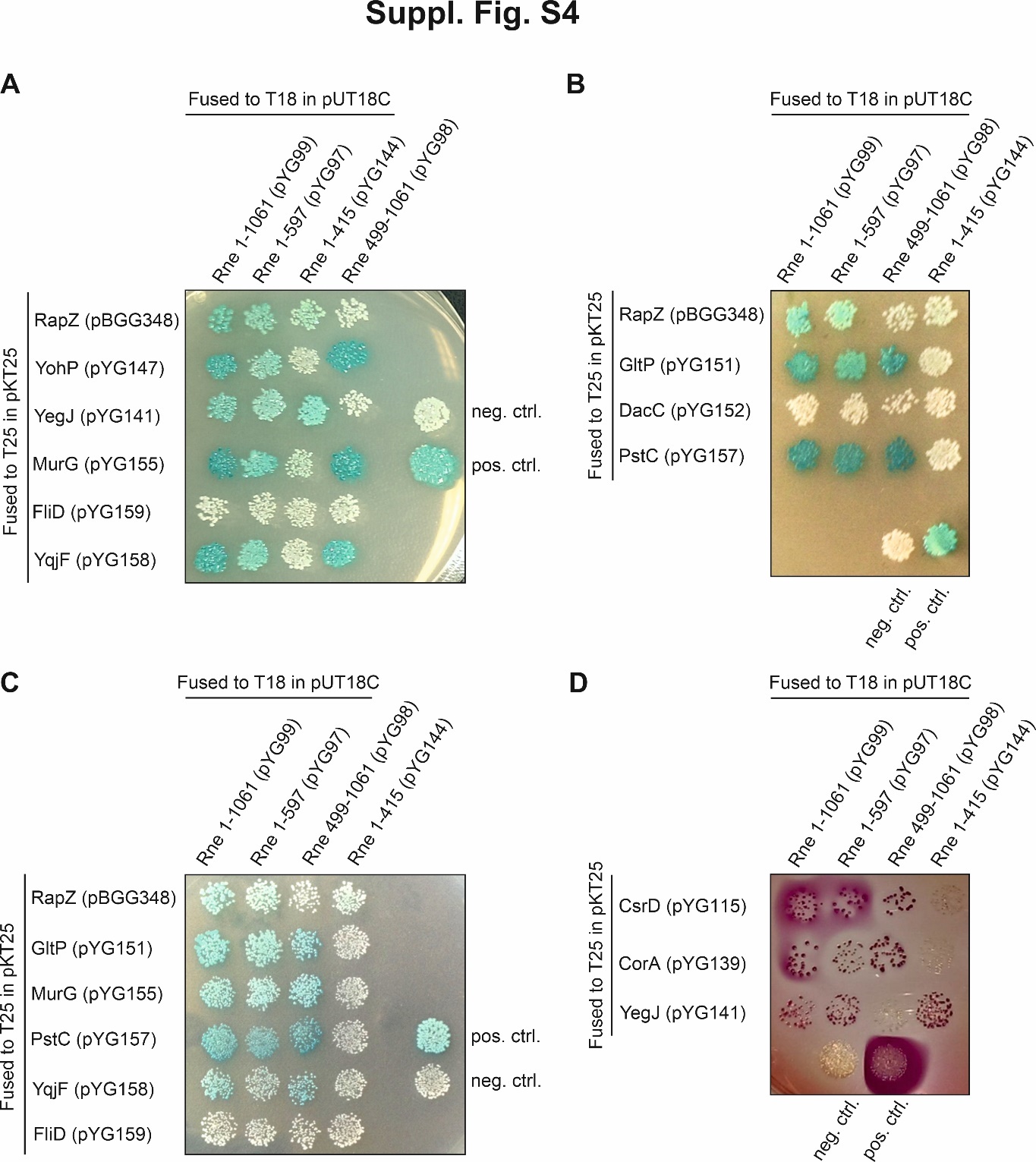


**Suppl. Fig. S4.** **BACTH spotting assays to assess interaction of full-length versions of candidate proteins fused to T25 with various Rne truncations fused to T18.** Double transformants of BTH101 carrying the indicated plasmids were spotted onto LB X-Gal plates **(A-C)** or on MacConkey maltose plates **(D)** and incubated at 28°C. Negative control: BTH101 pKT25/pUT18C; positive control: BTH101 pKT25-zip/pUT18C-zip.


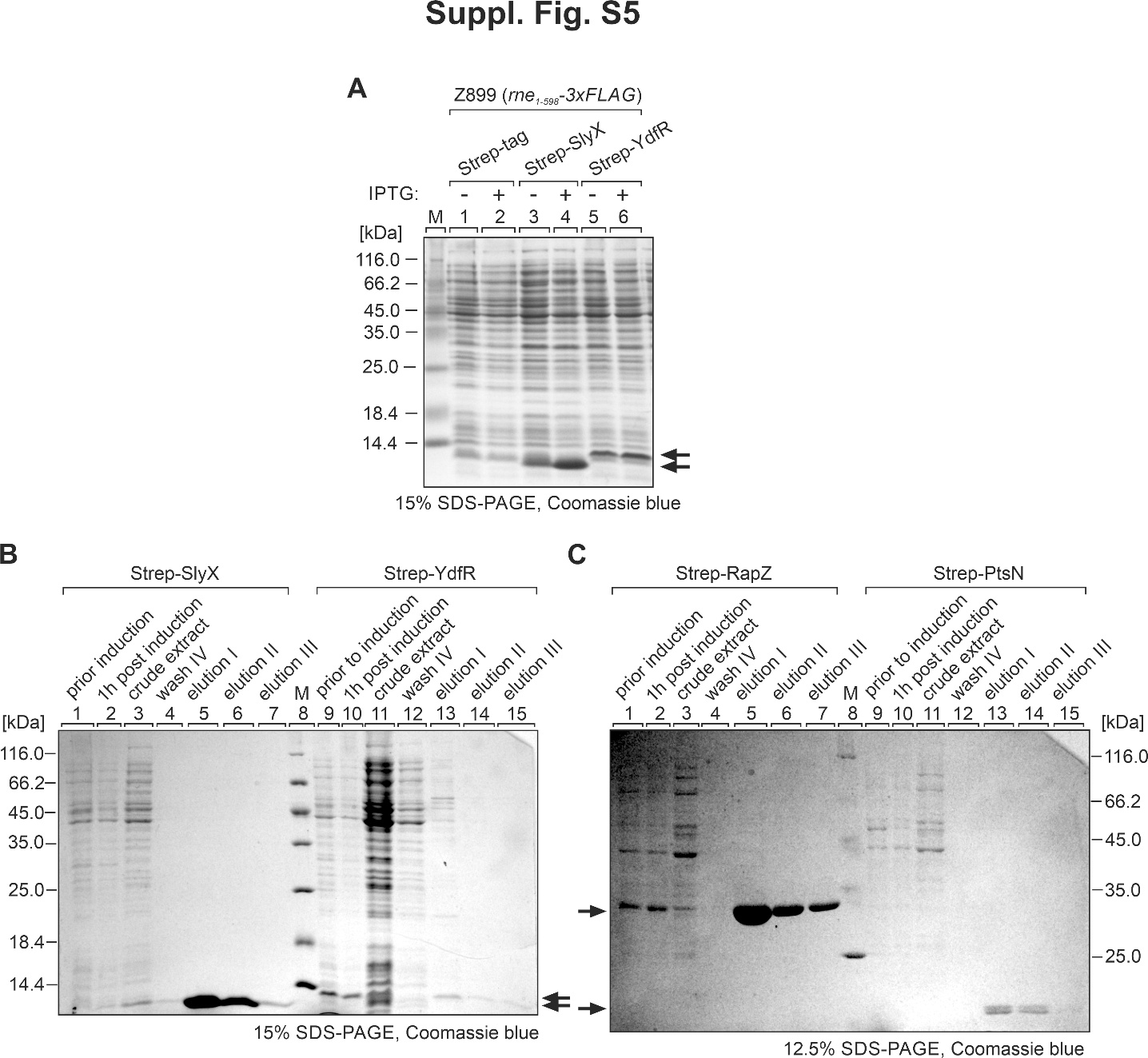


**Suppl. Fig. S5.** **Overproduction and StepTactin affinity purification of Strep-SlyX, Strep-YdfR, Strep-RapZ and Strep-PtsN.** **(A)** Overproduction of Strep-SlyX (MW = 9.54 kDa) and Strep-YdfR (MW = 13.42 kDa) in strain Z899. Transformants of strain Z899 carrying either plasmid pYG181 (*strep-slyX*, lanes 3-4), pYG182 (*strep-ydfR*, lanes 5-6) or the corresponding empty vector pBGG237 overproducing only the Strep-tag (lanes 1-2) were grown in LB until OD_600_ = 0.5-0.8. Following induction of protein overproduction with 1 mM IPTG as indicated and 1 h additional growth, the cells were harvested, and total protein extracts were separated by SDS-PAGE. The IPTG-treated cultures overproducing Strep-SlyX and Strep-YdfR (lanes 4, 6) were subjected to StrepTactin affinity purification shown in (B). **(B)** Analysis of samples collected at various steps during overproduction and StrepTactin affinity purification of Strep-YdfR and Strep-SlyX. **(C)** Analysis of samples collected during the overproduction and StrepTactin affinity purification of Strep-RapZ (MW = 33.82 kDa) and Strep-PtsN (MW = 19.29 kDa), which served as controls in the ligand fishing experiments. Strain Z899 carrying plasmid pBGG164 for overproduction of Strep-RapZ or plasmid pBGG217 for overproduction of Strep-PtsN was used.

**
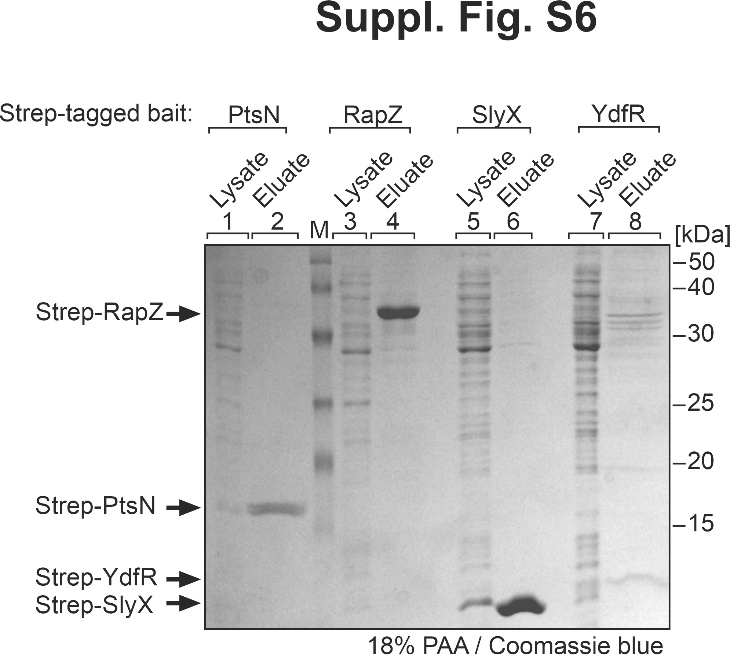
**

**Suppl. Fig. S6. Analysis of the samples of the copurification experiment shown in Fig. 3A by SDS-PAGE and Coomassie blue staining.** 10 μl of the samples analyzed in the ligand fishing experiments shown in Fig. 3A were separated on 18% SDS-PAA gels and gels were stained with Coomassie blue. Samples were loaded in the same order as in Fig. 3A. A protein molecular weight standard was loaded between lanes 2 and 3. Corresponding molecular weights [kDa] are indicated at the right.


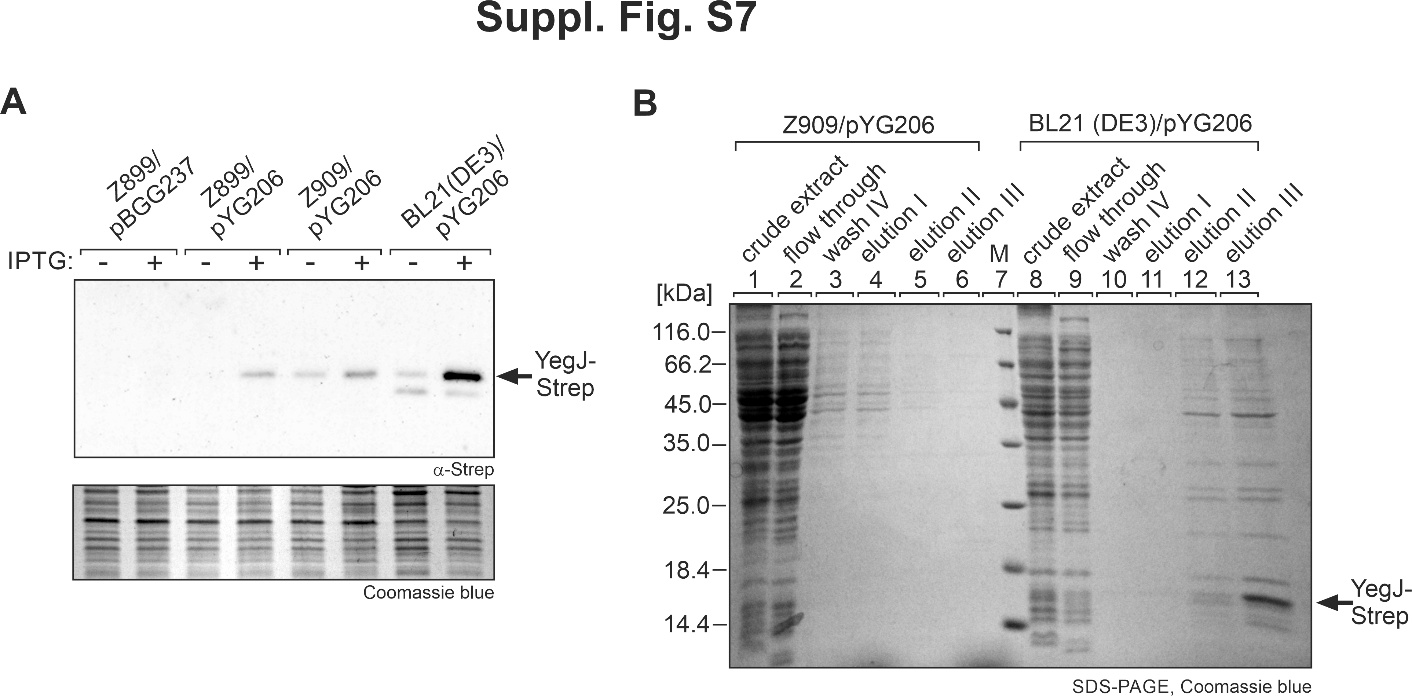


**Suppl. Fig. S7.** **Overproduction and StepTactin affinity purification of YegJ-strep. (A)** Overproduction of YegJ-strep (MW = 18.63) encoded on plasmid pYG206 in strains Z899, Z909 and BL21 (DE3). Strain Z899 carrying the empty vector pBGG237 served as control. The transformants were grown in LB until OD_600_ = 0.5-0.8. IPTG (1 mM) was added as indicated and following 1h growth, cells were harvested. Total protein extracts of the samples were separated on an SDS-PAA gel, which was subsequently analyzed by Western blotting using α-strep antiserum. A section of the Coomassie blue-stained PAA gel is shown to provide loading controls. **(B)** Analysis of samples collected at various steps during overproduction and StrepTactin affinity purification of Yeg-strep from strain Z909 (left half of the gel) and BL21 (DE3) (right half of the gel). A protein molecular weight standard was loaded in lane 7 and corresponding molecular weights [kDa] are indicated at the left.

**
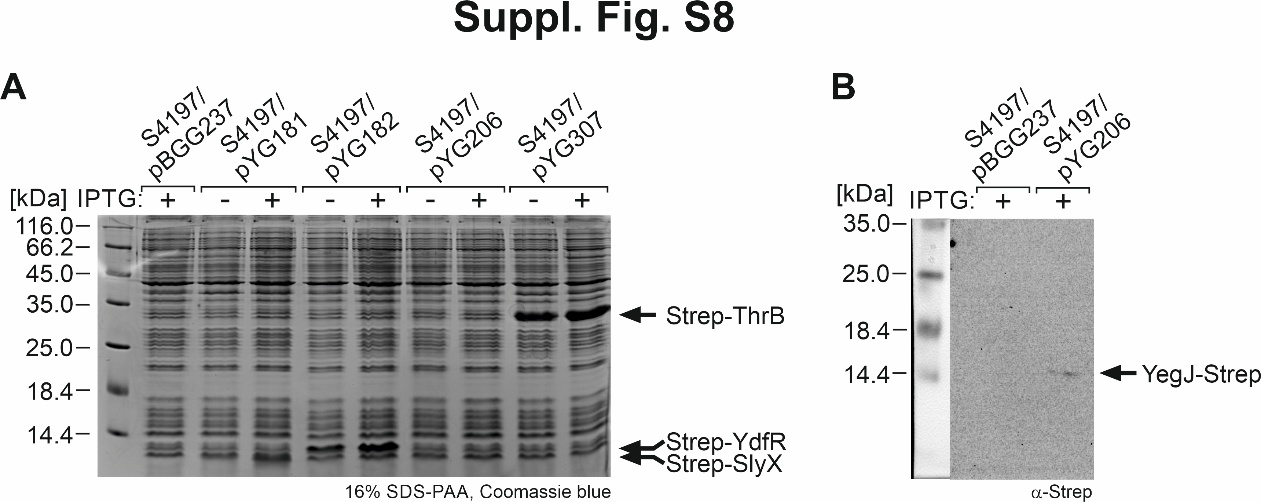
**

**Suppl. Fig. S8. Verification of overproduction of Strep-SlyX, Strep-YdfR, YegJ-Strep and Strep-ThrB in the cultures analyzed by RNA seq. (A)** SDS-PAGE analysis of total protein extracts of samples harvested from the cultures that were analyzed by RNA-seq. The indicated transformants were grown in LB until OD_600_ = 0.3. At this time, the “-IPTG” samples were harvested from the cultures. Following the addition of IPTG, a second set of samples was harvested 30 min later (+ IPTG samples). The SDS-PAA gel was stained with Coomassie blue to visualize proteins. A protein molecular weight standard was loaded in lane 1 and corresponding molecular weights [kDa] are indicated at the left. **(B)** Western blot analysis of IPTG-induced S4197/pYG206 cells using α-Strep antiserum. The transformant carrying the empty overexpression vector pBGG237 was included for comparison. The membrane showing the pre-stained bands of the protein molecular weight standard was added for size comparison.

**Suppl. Fig. S9**


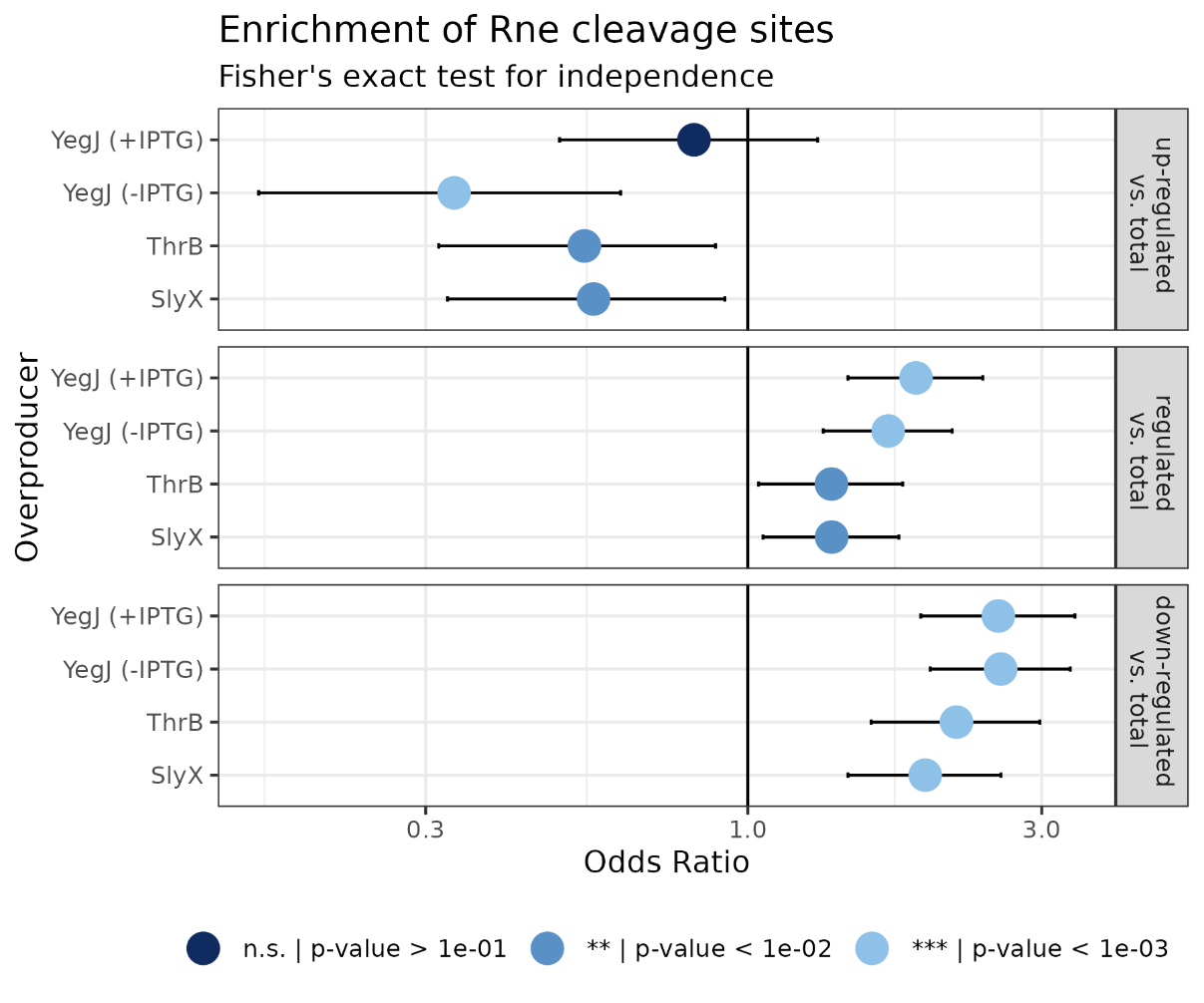


**Suppl. Fig. S9. Enrichment analysis of RNase E (Rne) cleavage sites across differentially expressed gene sets for multiple overproducers.** Up-regulated genes were defined as those with log₂ fold change ≥ 1 and adjusted p < 0.05, whereas down-regulated genes had log₂ fold change ≤ -1 and adjusted p < 0.05. Regulated genes comprise the combined set of up- and down-regulated genes. Each gene was additionally classified based on the presence or absence of an Rne cleavage site. Fisher's exact test was used to assess the association between Rne cleavage site presence and gene regulation category. Displayed is the estimated odds ratio, its 99% confidence interval, and the two-sided p-value, corrected for multiple testing using the Benjamini–Hochberg method (Benjamini and Hochberg 1995). Across all overproducers, down-regulated genes exhibit a significant enrichment of Rne cleavage site-containing genes (Fisher's exact test, adjusted p < 0.01, odds ratio > 1). Conversely, up-regulated genes exhibited a significant depletion of Rne cleavage site–containing genes for all overproducers except YegJ (+IPTG) (Fisher's exact test, adjusted p < 0.01, odds ratio < 1). Regulated genes exhibited again a significant enrichment of Rne cleavage site-containing genes for all overproducers (Fisher's exact test, adjusted p < 0.01, odds ratio > 1).


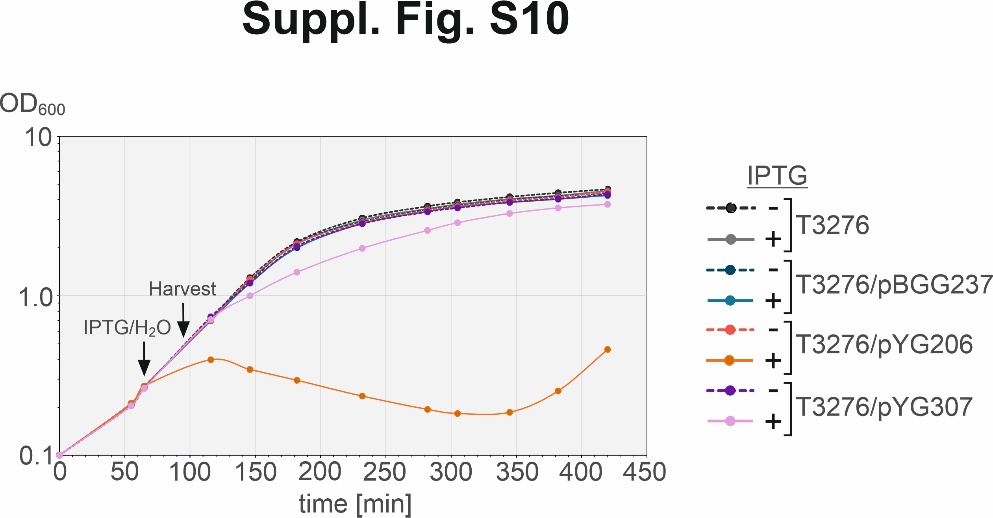


**Suppl. Fig. S10.** Exemplary growth curves for strain T3276 and its transformants carrying the vector control pBGG237, plasmid pYG206 for overexpression of *yegJ* or plasmid pYG307 for overexpression of *thrB* (referring to one set of replicates tested in Fig. 5E). The various strains were inoculated in LB to an initial OD_600_ = 0.1 and subsequently grown until cultures reached OD_600_~0.3. At this point, each culture was split into two subcultures, one of which received 1 mM IPTG for induction of plasmid-encoded gene expression, whereas the second culture received H_2_0 as mock. Growth was continued and the OD_600_ values were recorded at regular time intervals. The arrows refer to the time of addition of IPTG or H_2_O and to the time of harvesting cell suspensions for determination of ONPG hydrolysis rates, respectively.

**Suppl. Fig. S11**


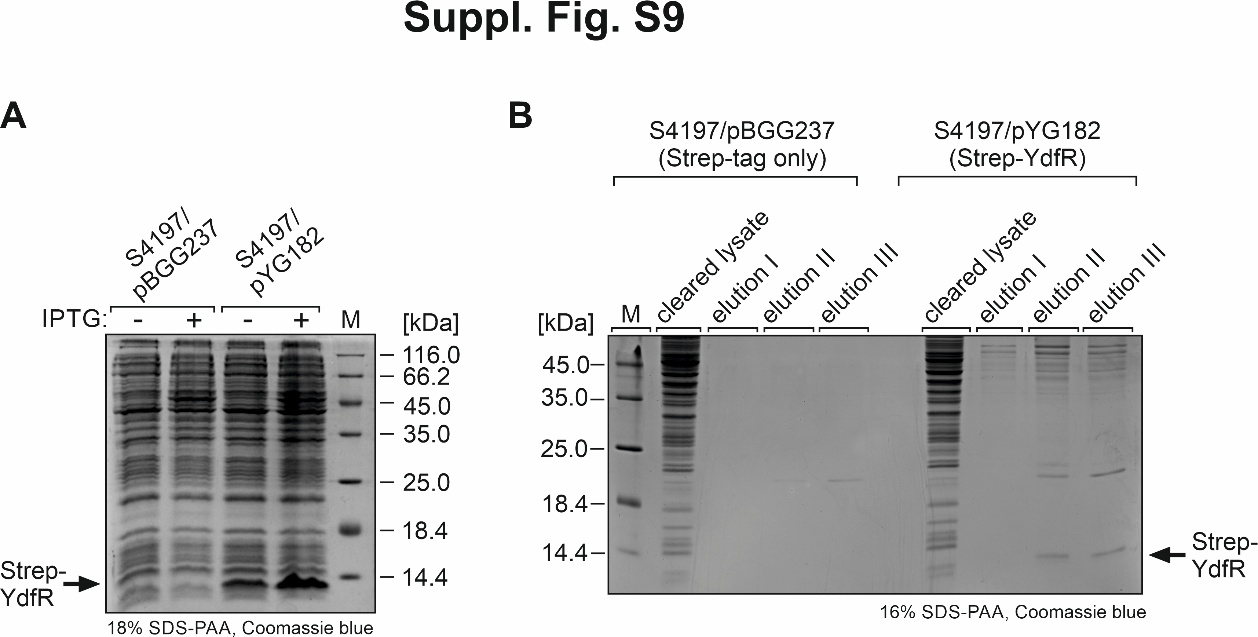


**Suppl. Fig. S11. Overproduction and StrepTactin affinity chromatography of Strep-YdfR.** **(A)** SDS-PAGE analysis of total protein extracts prepared from the transformants used for the subsequent StrepTactin affinity chromatography shown in (B). Cultures were grown in LB and induced with IPTG at mid-log growth phase. Cells were harvested after 1 h additional growth. A protein molecular weight standard was loaded in the last lane and corresponding molecular weights [kDa] are indicated at the right. **(B)** SDS-PAGE analysis of samples collected during StrepTactin affinity chromatography of Strep-YdfR. As a control, the same affinity purification was performed with cell extracts prepared from a transformant producing only the Strep peptide from an isogenic plasmid. The strain and plasmid names are indicated at the top. Nucleic acids were extracted from the elution fractions II. The measured nucleic acids concentrations were 150 ng/μl in the Strep-YdfR eluate and 8 ng/μl in the eluate of the Strep peptide purification. A protein molecular weight standard was loaded in lane 1 and corresponding molecular weights [kDa] are indicated at the left.

**Suppl. Fig. S12**

**
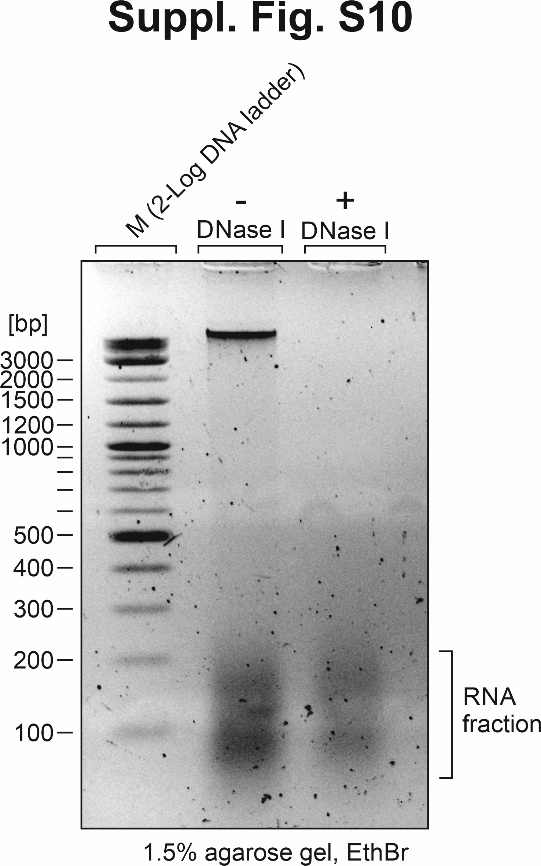
**

**Suppl. Fig. S12. Analysis of nucleic acids co-purifying with Strep-YdfR upon StrepTactin affinity chromatography by 1.5 % agarose gel electrophoresis and ethidium bromide staining.** Nucleic acids were extracted from elution fraction II of the Strep-YdfR purification shown in Suppl. Fig. S5B. 10 μl aliquots of the obtained nucleic acid solutions were separated by agarose gel electrophoresis. One of these aliquots was treated with 1 unit DNase I at 37°C for 1 h prior loading onto the gel.

**Suppl. Fig. S13**


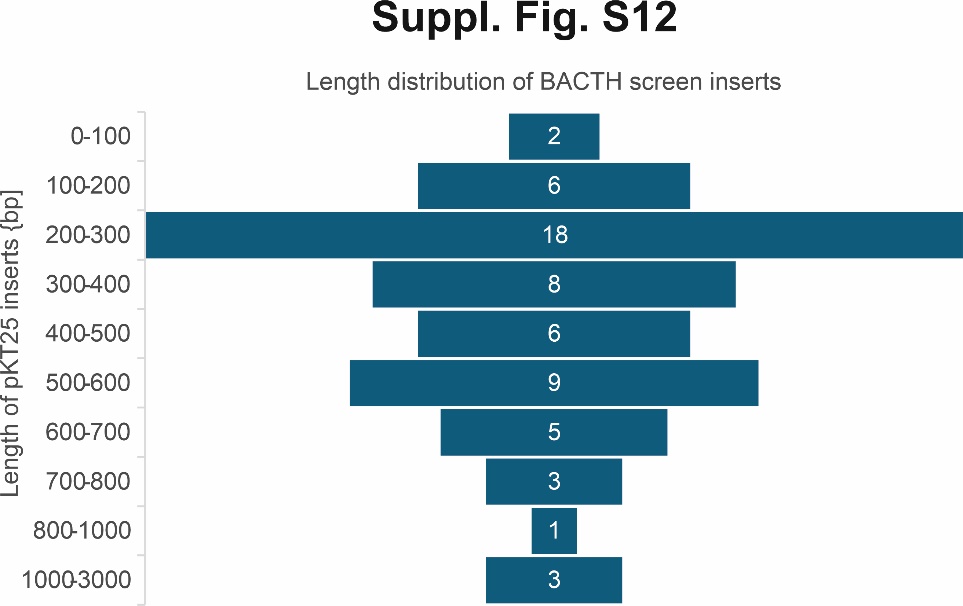


**Suppl. Fig. S13. Length distribution of the different inserts in the pKT25 library that were recovered in the BACTH screens for interaction with T18-rne bait constructs.** The length of the recovered DNA fragments is indicated on the Y-axis. The bars and the numbers in the bars indicate the number of different inserts matching the indicated length range.

**SUPPLEMENTARY TABLES**

**Suppl. Table S1.** Oligonucleotides used in this study.

| **Name^a^** | **Sequence^b,c,d^** | **Res. sites** | **Position^e^** |
| --- | --- | --- | --- |
| BG646 fwd | ttccgcgactcggcgcgc |  | pKT25 927-944 |
| BG647 rev | ggggatgtgctgcaaggcg |  | pKT25 1124-1106 |
| BG858 rev | aggGGTACCTTATAAGGTTGGGGTTTCTTCCC | KpnI | *rne* +1494 to +1475 |
| BG1110 fwd | gcgTCTAGA**G**atgaaaagaatgttaatcaacgc | XbaI | *rne* +1 to +23 |
| BG1111 rev | cgcGGTACCTTACTCAACAGGTTGCGGAC | KpnI | *rne* +3186 to +3167 |
| BG1114 rev | cgcGGTACCTTATTTCGGTGCTGGTTGCTCGG | KpnI | *rne* +1791 to 1774 |
| BG1130 rev | ggctctagaTTAAACCGAGTATCTTTGTGAATAT | XbaI | *csrD* +1941 to +1917 |
| BG1131 fwd | GGCTtctaga**G**atgagattaacgacgaaattttcG | XbaI | *csrD* +1 to +25 |
| BG1135 fwd | GGCTtctaga**G**atgaaccccatcgtaattaatcg | XbaI | *rnc* +1 to +23 |
| BG1136 rev | cgcGGTACCTCATTCCAGCTCCAGTTTTTTC | KpnI | *rnc* +681 to +660 |
| BG1137 fwd | GGCTtctaga**G**atgacggctgaattgttagtaaac | XbaI | *rng* +1 to +24 |
| BG1138 rev | cgcGGTACCTTACATCATTACGACGTCAAACTG | KpnI | *rng* +1470 to +1447 |
| BG1167 fwd | GGCTtctaga**G**atgAATTACGAATTACTGACCAC | XbaI | *rtcB* +1 to +23 |
| BG1168 rev | cgcGGTACCttaTCCTTTTACGCACACCACC | KpnI | *rtcB* +1227 to +1206 |
| BG1169 fwd | GGCTtctaga**G**atgCTGAGCGCATTTCAACTG | XbaI | *corA* +1 to +21 |
| BG1170 rev | cgcGGTACCTTACAACCAGTTCTTCCGCTTAAAG | KpnI | *corA* +951 to +927 |
| BG1171 fwd | GGCTtctaga**G**atgAAAAAGATATTACTTATGTTGAG | XbaI | *yegJ* +1 to +26 |
| BG1172 rev | cgcGGTACCTTAGCGAACTAATTTTGCTCTTAG | KpnI | *yegJ* +462 to +439 |
| BG1185 fwd | ggctTCTAGA**G**ATGAAAATTATACTCTGGGCTG | XbaI | *yohP* +1 to +22 |
| BG1186 rev | aaaaCTGCAGGGTACCttaAAATATCATCTTAAATACG | PstI, KpnI | *yohP* +84 to +63 |
| BG1189 fwd | ggctTCTAGA**G**ATGAAAAATATAAAATTCAGCCTGG | XbaI | *gltP* +1 to +25 |
| BG1190 rev | aaaaCTGCAGGGTACCttaTTGATCCGCAGTTTTATCAA | PstI, KpnI | *gltP* +1314 to +1292 |
| BG1191 fwd | ggctTCTAGA**G**ATGACGCAATACTCCTCTCTC | XbaI | *dacC* +1 to +21 |
| BG1192 rev | aaaaCTGCAGGGTACCttaAGAGAACCAGCTGCCGAAC | PstI, KpnI | *dacC* +1203 to +1181 |
| BG1198 fwd | ggctTCTAGA**G**ATGAGTGGTCAAGGAAAGCG | XbaI | *murG* +1 to +22 |
| BG1199 rev | aaaaCTGCAGGGTACCttaCGCCCGGGCAACCCG | PstI, KpnI | *murG* +1068 to +1051 |
| BG1202 fwd | ggctTCTAGA**G**ATGGCTGCAACCAAGCCTG | XbaI | *pstC* +1 to +19 |
| BG1203 rev | aaaaCTGCAGGGTACCttaGCGTGCCCCCTCATTC | PstI, KpnI | *pstC* +960 to +942 |
| BG1204 fwd | ggctTCTAGA**G**ATGAAAAAATTAGAAGATGTTGGTG | XbaI | *yqjF* +1 to +25 |
| BG1205 rev | aaaaCTGCAGGGTACCttaCCACTTTTTATTCAGCAGGC | PstI, KpnI | *yqjF* +393 to +371 |
| BG1206 fwd | ggctTCTAGA**G**ATGGCAAGTATTTCATCGCTG | XbaI | *fliD* +1 to +21 |
| BG1207 rev | aaaaCTGCAGGGTACCttaCTTGGAATTACTGTTGTTTTC | PstI, KpnI | *fliD* +1407 to + 1383 |
| BG1208 fwd | CTCAGGACACCTCCAAAGC |  | *fliD* +794 to +812 |
| BG1217 fwd | ggctTCTAGA**G**gcagaagcgaaaccggaacg | XbaI | *rne* +1792 to +1811 |
| BG1218 fwd | ggctTCTAGA**G**gaagaaaccaaaccgaccgag | XbaI | *rne* +1756 to +1776 |
| BG1226 fwd | gcTCTAGAgatgcaggatttatcattggaagc | XbaI | *slyX* +1 to +23 |
| BG1227 rev | gcccGGTACCtcaataatgtggcggtggcg | KpnI | *slyX* +219 to +199 |
| BG1228 fwd | gcTCTAGAGatgactcaagactatgaactgg | XbaI | *ydfR* +1 to +22 |
| BG1229 rev | gccGGTACCtcatagatatttatgatgaggacg | KpnI | *ydfR* +312 to +288 |
| BG1252 fwd | GGCTgctagcatgCAGGATTTATCATTGGAAGC | NheI | *slyX* +1 to +23 |
| BG1253 rev | tggCTGCAGtctagattatcaATAATGTGGCGGTGGCG | PstI, XbaI | *slyX* +219 to +200 |
| BG1254 fwd | GGCTgctagcatgACTCAAGACTATGAACTGG | NheI | *ydfR* +1 to +22 |
| BG1255 rev | cccAAGCTTTCTAGAttatcaTAGATATTTATGATGAGGAC | HindIII, XbaI | *ydfR* +312 to +290 |
| BG1308 mut | [P]-gggctgttgagccgc**GC**C**GC**Cggcgcactgaaagc |  | *rne* +1705 to +1739 |
| BG1335 fwd | ctcgtactcatatgAAAAAGATATTACTTATGTTG | NdeI | *yegJ* +1 to +24 |
| BG1336 rev | GGCTgctagcGCGAACTAATTTTGCTCTTAG | NheI | *yegJ* +462 to +436 |
| BG1566 fwd | gcgTCTAGA**g**atgCAGAAAGAACAACTTTCCGC | XbaI | *rseA* +1 to +23 |
| BG1567 rev | GGCCTGCAGGGTACCttaCTGCGATTGCGTTCCTAAAG | PstI, KpnI | *rseA* +705 to +629 |
| BG1568 fwd | gcgTCTAGA**g**atgGTTAAAGTTTATGCCCCGG | XbaI | *thrB* +1 to +22 |
| BG1569 rev | GGCCTGCAGGGTACCttaGTTTTCCAGTACTCGTGCG | PstI, KpnI | *thrB* +933 to +912 |
| BG1638 fwd | gcgTCTAGA**ga**tgACCATTACGAAACTTGCATG | XbaI | *ycbZ* +2 to +23 |
| BG1639 rev | gcgTCTAGAGGTACCttaGTTCGGAATAAACCAGTTCAG | XbaI, KpnI | *ycbZ* +1759 to +1738 |
| BG1640 fwd | gcgTCTAGA**g**atgCTAGTTGTAGAACTCATCAT | XbaI | *dcuA* +1 to +23 |
| BG1641 rev | gcgTCTAGAGGTACCttaCAGCATGAAGCTACCCAG | XbaI, KpnI | *dcuA* +1302 to +1282 |
| BG1642 fwd | ggctgctagcatgGTTAAAGTTTATGCCCCGG | NheI | *thrB* +1 to +22 |
| BG1643 fwd | gcgTCTAGA**g**atgCAAGATTTAAGCGGTTTCTC | XbaI | *wcaF* +1 to +23 |
| BG1644 rev | GGCCTGCAGGGTACCttaTTCAGTTTCAACGCGTTCG | PstI, KpnI | *wcaF* +549 to +528 |
| OB604 | GGAAGGCGAAGCGGCATGCATTTACGTTGACACCATCGAATGGCGCgtgtaggctggagctgcttcg |  |  |
| OB605 | CGGCCAGTGAATCCGTAATCATGGTCATAGCTGTTTCCTGTGTGAAATTttcccttcaggcgggattat |  |  |
| T462 | [P]‑TCGACTCACCCTTTGACGTGGTGATATGGATGACGGATAATCCCGCCTGAAGGGAAAG |  | *P_16_* |
| T463 | [P]‑GATCCTTTCCCTTCAGGCGGGATTATCCGTCATCCATATCACCACGTCAAAGGGTGAG |  | *P_16_* |

^a^ fwd: primer anneals to the antisense strand of the amplified gene; rev: primer anneals to the sense strand of the amplified gene; mut: mutagenesis primer used in CCR

^b^ Recognition sites of restriction endonucleases are underlined

^c^ [P]: Oligonucleotide carries a phosphate group at the 5’ OH of the 5’ terminal nucleotide

^d^ Nucleotide positions deviating from the *wild-type* sequence are in bold

^e^ annealing positions refer to the first bp of the indicated gene as annotated in the EcoCyc database (Karp et al. 2025) or plasmid.

**Suppl. Table S2.** Details on construction of plasmids.

| **Plasmid name** | **Vector backbone** | **Insert** | **Template for PCR or CCR** | **Restriction digest insert** |
| --- | --- | --- | --- | --- |
| pKES262 | pKD13 ΔSalI-BamHI | annealed oligonucleotides T462/T463 | n.a. | n.a. |
| pYG115 | pKT25/XbaI | pYG116 XbaI-XbaI (1948 bp) | n.a. | XbaI |
| pYG116 | pUT18C/XbaI | PCR BG1131 + BG1130 | chrom. DNA W3110 | XbaI |
| pYG121 | pKT25 ΔXbaI-KpnI | PCR BG1135 + BG1136 | chrom. DNA W3110 | XbaI + KpnI |
| pYG123 | pKT25 ΔXbaI-KpnI | PCR BG1137 + BG1138 | chrom. DNA W3110 | XbaI + KpnI |
| pYG124 | pUT18C ΔXbaI-KpnI | pYG123 XbaI-KpnI (1481 bp) | n.a. | XbaI + KpnI |
| pYG137 | pKT25 ΔXbaI-KpnI | PCR BG1167 + BG1168 | chrom. DNA W3110 | XbaI + KpnI |
| pYG139 | pKT25 ΔXbaI-KpnI | PCR BG1169 + BG1170 | chrom. DNA W3110 | XbaI + KpnI |
| pYG141 | pKT25 ΔXbaI-KpnI | PCR BG1171 + BG1172 | chrom. DNA W3110 | XbaI + KpnI |
| pYG144 | pKT25 ΔXbaI-KpnI | pSD3 XbaI-KpnI (1259 bp) | n.a. | XbaI + KpnI |
| pYG147 | pKT25 ΔXbaI-KpnI | PCR BG1185 + BG1186 | chrom. DNA W3110 | XbaI + KpnI |
| pYG151 | pKT25 ΔXbaI-KpnI | PCR BG1189 + BG1190 | chrom. DNA W3110 | XbaI + KpnI |
| pYG152 | pKT25 ΔXbaI-KpnI | PCR BG1191 + BG1192 | chrom. DNA W3110 | XbaI + KpnI |
| pYG155 | pKT25 ΔXbaI-KpnI | PCR BG1198 + BG1199 | chrom. DNA W3110 | XbaI + KpnI |
| pYG157 | pKT25 ΔXbaI-KpnI | PCR BG1202 + BG1203 | chrom. DNA W3110 | XbaI + KpnI |
| pYG158 | pKT25 ΔXbaI-KpnI | PCR BG1204 + BG1205 | chrom. DNA W3110 | XbaI + KpnI |
| pYG159 | pKT25 ΔXbaI-KpnI | PCR BG1206 + BG1207 | chrom. DNA W3110 | XbaI + KpnI |
| pYG162 | pUT18C ΔXbaI-KpnI | PCR BG1217 + BG1111 | chrom. DNA W3110 | XbaI + KpnI |
| pYG163 | pUT18C ΔXbaI-KpnI | PCR BG1218 + BG1111 | chrom. DNA W3110 | XbaI + KpnI |
| pYG165 | pUT18C ΔXbaI-KpnI | PCR BG1226 + BG1227 | chrom. DNA W3110 | XbaI + KpnI |
| pYG166 | pUT18C ΔXbaI-KpnI | PCR BG1228 + BG1229 | chrom. DNA W3110 | XbaI + KpnI |
| pYG168 | pUT18C ΔXbaI-KpnI | PCR BG1110 + BG858 | chrom. DNA W3110 | XbaI + KpnI |
| pYG174 | pKT25 ΔXbaI-KpnI | pYG165 XbaI-KpnI (230 bp) | n.a. | XbaI + KpnI |
| pYG175 | pKT25 ΔXbaI-KpnI | pYG166 XbaI-KpnI (323 bp) | n.a. | XbaI + KpnI |
| pYG181 | pBGG164 ΔNheI-PstI | PCR BG1252 + BG1253 | pYG174 | NheI + PstI |
| pYG182 | pBGG164 ΔNheI-PstI | PCR BG1254 + BG1255 | pYG175 | NheI + PstI |
| pYG195 | pYG97 ΔAflII-KpnI | CCR BG1110 + BG1114 + BG1308 | pYG97 | AflII + KpnI |
| pYG206 | pYG191 ΔNdeI-NheI | PCR BG1335 + BG1336 | pYG141 | NdeI + NheI |
| pYG286 | pKT25 ΔXbaI-KpnI | PCR BG1566 + BG1567 | chrom. DNA W3110 | XbaI + KpnI |
| pYG287 | pKT25 ΔXbaI-KpnI | PCR BG1568 + BG1569 | chrom. DNA W3110 | XbaI + KpnI |
| pYG307 | pBGG164 ΔNheI-PstI | PCR BG1642 + BG1569 | chrom. DNA W3110 | NheI + PstI |
| pYG308 | pKT25 ΔXbaI-KpnI | PCR BG1643 + BG1644 | chrom. DNA W3110 | XbaI + KpnI |
| pYG309 | pKT25 ΔXbaI-KpnI | PCR BG1638 + BG1639 | chrom. DNA W3110 | XbaI + KpnI |
| pYG310 | pKT25 ΔXbaI-KpnI | PCR BG1640 + BG1641 | chrom. DNA W3110 | XbaI + KpnI |

**Suppl. Table S3.** Prey clones identified in the BACTH screens for interaction partners of Rne (or Rng) with libraries in pKT25.

| **Amino acids fused to T25 in pKT25****^*^** | **UniProt acc. no.** | **Function of the full-length protein** | **Genome coordinates of insert in pKT25^$^** | **Bait plasmid / screened library (identified no.)** | **Names of representative isolates** |
| --- | --- | --- | --- | --- | --- |
| Aer 36-220^*^ | P50466 | aerotaxis receptor | 3.218.976-3.218.434 | pYG97 / GB4 (1) | rneN-10 |
| AmpE 219-284^*^ | P0AE14 | uncharacterized | 119.936-120.163 | pYG97 / GB1 (1) | RneM-9 |
| BaeS 1-36^*^ | P30847 | histidine kinase | 2.162.778-2.162.982 | pYG97 / GB4 (1) | rneN-100 |
| CadC 106-175^*^ | P23890 | transcript. activator | 4.361.621-4.361.408 | pYG195 / GB-BstZ17I (2) | pYG195_D_c70, c75 |
| CorA 118-316^*^ | P0ABI4 | Mg^2+^ transp. | 4.001.778-4.002.432 | pYG99 / GB1 (14)  pYG195 / GB-BstZ17I (1) | Rnef-9, 10, 11, 14, 19, 20, 21, 22, 23, 25, 26, 27, 31, 33  pYG195_D_c35 |
| CydH (= YnhF) 1-29 (FL) ^*^ | A5A618 | SU cytochrome *bd*-I complex | 1.737.565-1.737.054 | pYG195 / GB-BstZ17I (1) | pYG195_D_c36 |
| DacA 347-403^*†^ | P0AEB2 | PBP5 | 662.927-662.643 | pYG97 / GB1 (1) | 97_N_1 |
| DacA 385-403^*^ | P0AEB2 | PBP5 | 662.813-662.428 | pYG195 / GB-BstZ17I (2) | pYG195_D_c34, c59 |
| DacC 270-400^*†^ | P08506 | PBP6 | 881.535-882.247 | pYG97 / GB4 (1)  pYG97 / GB5 (1) | rneN-8  rneN-152 |
| DcuA 377-433**^**^**^†^ | P0ABN5 | C4-dicarboxylate transp. | 4.365.647-4.365.401 | pYG97 / GB1 (1)  pYG144 / GB1 (1) | RneM-5  Rne415-5 (=rne415-1.38) |
| DgcN (= YfiN) 15-189^*^ | P46139 | diguanylate cyclase | 2.742.423-2.742.954 | pYG97 / GB5 (1) | rneN-157 |
| EptA 128-271^*^ | P30845 | pEtN transferase | 4.335.208-4.334.778 | pYG99 / GB1 (1) | Rnef-3 |
| FliD 266-420 | P24216 | flagellar filament capping protein | 2.004.665-2.005.131 | pYG97 / GB5 (34)^a^  pYG195 / GB-BstZ17I (4) | rneN-128, rneN-131, rneN-138, rneN-159  pYG195_D_c15 |
| FtsN 22-197^*^ | P29131 | cell division protein | 4.123.278-4.122.749 | pYG195 / GB-BstZ17I (1) | pYG195_D_c54 |
| GlpC 1-69 + 21aa from *gshA* rc strand | P0A996 | anaerobic glycerol-3-phosphate dehydrog. | 2.355.521- 2.355.727+  2.816.130- 2.816.472 | pYG144 / GB4 (1) | Rne415-6 (=rne415-4.1) |
| GltP 1-437 (FL) ^*^ | P21345 | Glu/Asp: H^+^ symporter | 4.294.473-4.297.055 | pYG97 / GB4 (2)  pYG97 / GB5 (16)  pYG195 / GB-BstZ17I (1) | rneN-5, rneN-19  rneN-151  pYG195_D_c14 |
| GltP 255-434^*^ | P21345 | Glu/Asp: H^+^ symporter | 4.295.243-4.295.775 | pYG144 / GB1 (1) | Rne415-2 (=rne415-1.13) |
| MurG 2-133 | P17443 | UDP-GlcNAc transf. | 99.645-100.043 | pYG97 / GB4 (2) | rneN-26, rneN-52 |
| NarQ 1-32^*†^ | P27896 | histidine kinase | 2.585.726-2.585.825 | pYG195 / GB-BstZ17I (2) | pYG195_D_c57, c83 |
| NimT (= YeaN) 4-393^*^ | P76242 | 2-nitroimidazole exporter | 1.875.680-1.877.927 | pYG97 / GB4 (1) | rneN-29 |
| PstC 14-35^*^ | P0AGH8 | SU phosphate ABC transp. | 3.910.361-3.910.298 | pYG97 / GB4 (1)  pYG97 / GB5 (1) | rneN-73  rneN-161 |
| Rne 506-598 | P21513 | endoribonuclease E | 1.142.851-1.142.573 | pYG195 / GB-BstZ17I (2) | pYG195_D_c67, c62 |
| RseA 63-216^*^ | P0AFX7 | anti-sigma-E factor | 2.709.217-2.708.629 | pYG195 / GB-BstZ17I (9) | pYG195_D_c3, c31, c43, c65 |
| RtcB 113-205 | P46850 | 3'-5' RNA ligase | 3.557.745-3.557.466 | pYG99 / GB1 (1) | Rnef-6 |
| SlyX 1-72 (FL) | P0A8R4 | uncharacterized | 3.477.638-3.477.975 | pYG124 / GB1 (1) | pKT25-GB1-10 |
| ThrB 268-310 | P00547 | homoserine kinase | 3600-3953 | pYG195 / GB-BstZ17I (4) | pYG195_D_c10, c30, c78 |
| TorC 1-124^*^ | P33226 | *c*-type cytochrome | 1.058.064-1.058.457 | pYG195 / GB-BstZ17I (1) | pYG195_D_c84 |
| TtdT (=YgjE) 455-487^*^ | P39414 | tartrate/succinate antiport | 3.209.372-3.209.608 | pYG97 / GB4 (2)  pYG97 / GB5 (1) | rneN-2, rneN-3  rneN-150 |
| UbiD 344-393 | P0AAB4 | ubiquinol synthesis | 4.026.015-4.026.166 | pYG97 / GB1 (1) | RneN-M1 |
| UspB 50-111^*^ | P0A8S5 | universal stress protein | 3.639.575-3.639.276 | pYG195 / GB-BstZ17I (1) | pYG195_D_c96 |
| WcaF 10-182 | P0ACD2 | acetyltransferase | 2.128.863-2.128.328 | pYG97 / GB1 (1) | RneN-M3 |
| XapR 98-294 | P23841 | transcript. activator | 2.522.188-2.521.246 | pYG195 / GB-BstZ17I (1) | pYG195_D_c28 |
| YbbP 1-267^*^ | P77504 | SU of putative transp. | 520.324-520.947 | pYG97 / GB5 (1) | rneN-174 |
| YbhG 1-97 | P75777 | put. drug efflux transp. | 830.055-829.681 | pYG195 / GB-BstZ17I (1) | pYG195_D_c51 |
| YcbZ 565-586 | P75867 | put. protease | 1.016.603-1.016.343 | pYG97 / GB5 (1) | rneN-212 |
| YdiN 193-382^*^ | P76198 | put. transporter | 1.773.074-1.773.656 | pYG99 / GB1 (1) | Rnef-2 |
| YdfR 1-72 | P76160 | Qin prophage protein | 1.640.418-1.640.151 | pYG124 / GB1 (1)  pYG124 / GB5 (2) | pKT25-GB1-9  pKT25-GB5-6, pKT25iii1-2 |
| YegJ 16-153 | P76394 | DUF2314 domain-containing protein; uncharacterized | 2.151.228-2.151.658 | pYG99 / GB1 (3)  pYG97 / GB1 (11)^a^  pYG144 / GB1 (34)^a^ | Rnef-15, 17, 29  Rne-M2, M4, M6, M10, M12, M15, M21, M24, M29, M31  Rne415-1 |
| YfcC 464-506^*^ | P39263 | put. Transp. | 2.418.468-2.418.818 | pYG195 / GB-BstZ17I (1) | pYG195_D_c52 |
| YfgM 1-206 (FL)^*^ | P76576 | ancillary SU of SecYEG | 2.639.309-2.638.572 | pYG195 / GB-BstZ17I (1) | pYG195_D_c87 |
| YfjP 259-287 | P52131 | CP4-57 prophage prot. | 2.768.485-2.768.711 | pYG195 / GB-BstZ17I (2) | pYG195_D_c2, c56 |
| YgaZ 107-249^*^ | P76630 | L-valine export | 2.809.927-2.810.394 | pYG97 / GB5 (1) | rneN-197 |
| YjhF 414-449^*^ | P39357 | put. transp. | 4.520.783-4.520.286 | pYG97 / GB4 (1) | rneN-16 |
| YohP 1-27 (FL)^*^ | C1P609 | uncharacterized | 2.228.950-2.229.248 | pYG97 / GB4 (3) | rneN-1, rneN-4, rneN-7 |
| YphA 1-140 (FL)^*^ | P0AD47 | uncharacterized | 2.673.314-2.673.769 | pYG195 / GB-BstZ17I (5) | pYG195_D_c5, c29, c61 |
| YqjF 1-35^*^ | P42619 | uncharacterized | 3.250.467- 3.250.656 | pYG97 / GB4 (1)  pYG97 / GB5 (1) | rneN-97  rneN-158 |

**^*^** Protein fragment fused to T25 contains at least one transmembrane domain

**^**^**Protein fragment contains a hydrophobic region that is discussed to form a TMD or to associate with the membrane (Golby et al. 1998).

^†^ Soluble part of this protein fragment is thought to be localized in the periplasm

**^$^** Inserts in SmaI-site of pKT25; positions refer to the genome sequence of strain MG1655 (GenBank accession number U00096.3)

^a^ Only representative isolates were analyzed by sequencing; the total number was obtained by testing clones that yielded fragments of identical length in PCRs with oligos BG646+BG647 through an additional PCR using a primer combination specific for the insert: BG1208+BG647 for *fliD* and BG646+BG1172 for *yegJ*.

**Suppl. Table S4.** Prey clones identified in the BACTH screens for interaction partners of Rne or Rng that carry non-annotated *orfs* or the reverse complement of annotated *orfs* fused to *T25*.

| **Genome coord. of insert in pKT25^$^** | **Description of *orf* fused in frame to *T25*** | **Bait plasmid / screened library (identified no.)** | **Names of isolates** |
| --- | --- | --- | --- |
| 129.957-130.692 | 39 aa *orf* on opposite strand of *yacH* | pYG99 / GB1 (1) | Rnef-1 |
| 335.037-334.706 | 56 aa *orf* overlapping with *yahC*, but different frame | pYG195 / GB-BstZ17I (1) | pYG195_D_c53 |
| 823.305-823.069 | 68 aa *orf* within *clsB*, but different frame | pYG99 / GB1 (1) | Rnef-4 |
| 1.027.155-1.026.548 | 38 aa *orf* starting in the *yccT* 3’ end, but different frame | pYG97 / GB5 (1) | rneN-203 |
| 1.142.125-1.141.912 | 71 aa *orf* on opposite strand of *rne* | pYG195 / GB-BstZ17I (1) | pYG195_D_c38 |
| 1.434.173-1.434.325 | 34 aa *orf* starting on opposite strand of the *ynaE* 5’ end | pYG97 / GB1 (1) | RneM-38 |
| 1.578.308-1.578.127 | 38 aa *orf* within *yddA,* but different frame | pYG97 / GB4 (1) | rneN-116 |
| 2.575.982-2.576.334 | 17 aa *orf* on opposite strand of *maeB-eutS*-IGS | pYG97 / GB4 (1) | rneN-78 |
| 2.872.468-2.872.692 | 78 aa orf on opposite strand of *ispD*-*ftsB* | pYG99 / GB1 (1) | Rnef-7 |
| 3.118.790-3.118.982 | 66 aa orf on opposite strand of *yghJ* | pYG195 / GB-BstZ17I (2) | pYG195_D_c72, c74 |
| 3.993.558-3.991.098 | *cyaA* aa 1-802 on opposite strand of insert | pYG124 / GB5 (2) | pKT25-GB5-34; pKT25-GB1-2 |
| 4.154.134-4.153.874 | 30 aa *orf* within *argE*, but different frame | pYG195 / GB-BstZ17I (1) | pYG195_D_c48 |
| 4.293.656-4.293.897 | 86 aa *orf* within *nrfG*, but different frame | pYG97 / GB1 (1) | RneM-30 |
| 4.501.355-4.501.656 | 48 aa orf starting in the *yjgZ* 3’ end, but different frame | pYG99 / GB1 (1) | Rnef-18 |
| 4.629.791-4.629.634 | 57 aa *orf* within *ettA*, but different frame | pYG195 / GB-BstZ17I (1) | pYG195_D_c91 |

**^$^** Inserts in SmaI-site of pKT25; positions refer to the genome sequence of strain MG1655 (GenBank accession number U00096.3)

**Suppl. Table S5**: Interaction of full-length candidate proteins fused to T25 with various RNase E truncations fused to T18. The reported values refer to the β-galactosidase activities (in Miller units ± standard deviation) determined for strain BTH101 carrying the indicated plasmid combinations. White/blue (wh./bl.) refers to the colony phenotypes observed in spotting assays on LB X-Gal and colorless/red (co./re.) to the colony phenotypes observed in spotting assays on MacConkey maltose plates, respectively. The β-galactosidase activities and phenotypes determined for the T25-enolase (Eno), T25-CsrD and T25-RapZ fusion constructs were added for comparison. Blue labels indicate higher β-galactosidase activities as compared to the negative control (empty plasmids pKT25/pUT18C). Yellow labels indicate β-galactosidase activities close to the negative control.

| **Candidate** | **Plasmid with T25 fusion** | **Rne-FL (pYG99)** | **Rne 1-597 (pYG97)** | **Rne 1-597^FF-AA^ (pYG195)** | **Rne 1-498 (pYG168)** | **Rne 1-415 (pYG144)** | **Rne 499-1061**  **(pYG98)** | **Rne 586-1061 (pYG163)** | **Rne598-1061 (pYG162)** |
| --- | --- | --- | --- | --- | --- | --- | --- | --- | --- |
| **Eno** | pYG95 | 573±194, bl. | 83±27, wh. | 174±69 | 75±36 | 85±36, wh. | 1504±491, bl. | 497±200, bl. | 557±236, bl. |
| YqjF | pYG158 | bl. | 549±223, bl. | 648±228 | 74±20 | 83±29, wh. | 1178±550, bl. | 71±15 | 163±88 |
| YohP | pYG147 | bl. | 645±244, bl. | 765±425 | 72±21 | 67±20, wh. | 1102±565, bl. | 64±6 | 64±7 |
| CorA | pYG139 | 4429±291, re. | 2023±519, re. | 1280±735 | 40±3 | 63±9, co. | 3928±954, re. | 62±13 | 47±14 |
| YfgM | pYG195_c87 | n.d. | 2514±261 | 2411±68 | 94±37 | 163±67 | 2815±1001 | 94±26 | 68±16 |
| GltP | pYG151 | bl. | 2606±40, bl. | 2520±114 | 376±220 | 174±84, wh. | 2065±102, bl. | 87±32 | 89±45 |
| MurG | pYG155 | bl. | 2675±517, bl. | 2496±337 | 112±49 | 189±42, wh. | 4157±1166, bl. | 101±11 | 128±81 |
| CydH | pYG195_c36 | n.d. | 3330±223 | 2838±847 | 157±47 | 116±34 | 5296±775 | 105±18 | 140±29 |
| YphA | pYG195_c5 | n.d. | 3628±531 | 2459±435 | 186±150 | 93±17 | 6114±1059 | 83±8 | 118±42 |
| PstC | pYG157 | bl. | bl. | n.d. | n.d. | wh. | bl. | n.d. | n.d. |
| **CsrD** | pYG115 | 4179±634, bl./re. | 3540±1132, bl./re. | 1634±409 | 88±21 | 63±11, wh./co. | 2447±229, bl./re. | 67±15, wh. | 49±3, wh. |
| ThrB | pYG287 | n.d. | 1167±72 | 934±123 | 132±62 | 70±22 | 82±26 | 77±25 | 78±23 |
| YdfR | pYG175 | 117±78 | 833±434 | 1300±460 | 216±112 | 148±119 | 76±27 | 123±3 | 83±0 |
| SlyX | pYG174 | 1108±517 | 673±238 | 2163±89 | 1223±277 | 436±136 | 105±32 | 93±11 | 64±3 |
| YegJ | pYG141 | 1454±292, bl./re. | 2586±1096, bl./re. | 3375±97 | 5012±1306 | 4555±769, bl./re. | 90±9, wh./co. | 80±25, wh. | 114±44 |
| **RapZ** | pBGG348 | 2836±736, bl. | 2398±837, bl. | 2721±176 | 1246±592 | 2636±921, bl. | 121±51, wh. | 78±26, wh. | 136±72, wh. |
| WcaF | pYG308 | n.d. | 3380±159 | 2952±422 | 1903±387 | 1711±261 | 4660±462 | 125±75 | 85±39 |
| YcbZ | pYG309 | n.d. | 3116±10 | 3167±175 | 2679±262 | 1804±154 | 5064±504 | 51±7 | 48±2 |
| DacC | pYG152 | wh. | 95±33, wh. | 73±16 | 57±4 | 56±6, wh. | 159±88, wh. | 67±4 | 47±0 |
| DcuA | pYG310 | n.d. | 123 | 155 | 113 | 128 | 139 | 57 | 109 |
| FliD | pYG159 | wh. | wh. | n.d. | n.d. | wh. | wh. | n.d. | n.d. |
| RseA | pYG286 | 51±4 | n.d. | 48±1 | n.d. | n.d. | n.d. | n.d. | n.d. |
| RtcB | pYG137 | 73 | 67 | n.d. | n.d. | n.d. | 68 | n.d. | n.d. |

n.d. = not determined.

**Suppl. Table S6**. Genes that are differentially regulated upon overexpression of *slyX* as compared to empty vector (S4197 pBGG237) and are not similarly regulated by *ydfR*, *yegJ* or *thrB* overexpression. Genes are listed, which are regulated in the RNA-seq analysis of the SlyX overproducer with log2 fold change ≥ 1.5 or ≤ -1.5 (adjusted p-value ≤ 0.05). From this list, all genes were eliminated that are also regulated by at least one of the other overproducers (YdfR, ThrB or YegJ) in the same direction (same algebraic sign) with a log2 fold change ≥ 0.5 or ≤ -0.5 (adjusted p-value ≤ 0.1), respectively.

| **Gene** | **Funktion** | **log2 fold change** | **adjusted**  **p-value** | **∅ normalized reads in overproducer** | **∅ normalized reads in S4197 pBGG237** |
| --- | --- | --- | --- | --- | --- |
| *slyX* | uncharacterized | 8,745 | 6,28E-234 | 688076.7 | 1603.9 |
| *yjdN* | uncharacterized | 2.032 | 0,00542 | 26.7 | 6.6 |
| *yibT* | uncharacterized | 2,019 | 0,0000000319 | 4872.8 | 1202.2 |
| *umuD* | DNA pol. V (SOS) | 2,018 | 0,0000000407 | 154.2 | 38.2 |
| *mdtP* | multidrug efflux pump | 1,896 | 0,000000164 | 101.7 | 27.2 |
| *yidI* | uncharacterized | 1,787 | 0,000000000000277 | 423.5 | 122.5 |
| *yhhZ*^1^ | putative endonuclease | 1,684 | 0,0273 | 24.1 | 7.5 |
| *hsrA* | Multidrug efflux pump | 1,664 | 0,0000000000000000674 | 1006.4 | 317.4 |
| *yhhY*^1^ | N-acetyltransferase | 1,655 | 0,0000169 | 503.1 | 159.4 |
| *dgoT* | galactonate H^+^ symporter | 1,576 | 0,00000119 | 160.3 | 53.7 |
| *sulA* | cell division inhibitor (SOS) | 1,522 | 0,00000000000491 | 1960.0 | 681.9 |
| *rsxG^2^* | SoxR [2Fe-2S] reducing prot. | -1,559 | 0,00000000000463 | 255.1 | 751.8 |
| *yciH* | putative translation factor | -1,581 | 0,000147 | 91.9 | 275.6 |
| *trmD*^3^ | tRNA methyltransferase | -1,618 | 0,0000000157 | 16265.1 | 49924.0 |
| *yraQ* | uncharacterized | -1,645 | 0,0000000192 | 278.1 | 869.9 |
| *rapA* | RNAP recycling factor | -1,670 | 0,0000138 | 1578.0 | 5022.2 |
| *rpsP*^3^ | ribosomal S16 protein | -1,689 | 0,0000000000734 | 5768.9 | 18598.8 |
| *rimM*^3^ | 30S assembly factor | -1,693 | 0,000000232 | 11424.6 | 36930.2 |
| *ydgI* | amino acid/polyamine antiporter | -1,698 | 0,0013 | 179.3 | 582.1 |
| *yegQ* | tRNA hydroxylation protein | -1,714 | 0,00000334 | 693.3 | 2274.8 |
| *nth^2^* | endonuclease III | -1,723 | 0,0000000000000000185 | 281.4 | 929.2 |
| *rsxE^2^* | SoxR [2Fe-2S] reducing prot. | -1,765 | 0,00000000000000666 | 194.6 | 661.6 |
| *rimO* | S12 methylthiotransferase | -1,841 | 0,000000000106 | 760.1 | 2723.8 |
| *obgE* | GTPase | -1,868 | 0,000000446 | 879.4 | 3211.0 |
| *rpsF* | ribosomal S6 protein | -1,895 | 0,00000000000000629 | 23393.1 | 87003.7 |
| *rplC* | ribosomal L3 protein | -1,933 | 0,000000000000538 | 37763.7 | 144180.0 |
| *ydiY* | outer membrane receptor | -2,003 | 0,0000000507 | 380.2 | 1524.7 |
| *rluB* | 23S rRNA pseudouridine synth. | -2,283 | 0,000000000179 | 485.9 | 2365.2 |
| *mgtA* | Mg^2+^ transporting P-type ATPase | -2,330 | 0,00000022 | 883.4 | 4443.6 |
| *yoeI* | uncharacterized | -3,018 | 0,013 | 83.4 | 675.8 |

^1^genes that are likely co-transcribed together (operon) are indicated by same superscript numbers.

**Suppl. Table S7**. Genes that are differentially regulated upon overexpression of *ydfR* as compared to empty vector (S4197 pBGG237) and are not similarly regulated by *slyX*, *yegJ* or *thrB* overexpression. Genes are listed that are regulated in the RNA-seq analysis of the YdfR overproducer with log2 fold change ≥ 1.5 or ≤ -1.5 (adjusted p-value ≤ 0.05). From the latter list, all genes were eliminated that are also regulated by at least one of the other overproducers (SlyX, ThrB or YegJ) in the same direction (same algebraic sign) with a log2 fold change ≥ 0.5 or ≤ -0.5 (adjusted p-value ≤ 0.1), respectively.

| **Gene name** | **Function** | **log2 fold change** | **adjusted**  **p-value** | **mean normalized reads in overproducer** | **mean normalized reads in S4197 pBGG237** |
| --- | --- | --- | --- | --- | --- |
| *ydfR** | uncharacterized | 15,706 | 2,52E-239 | 373135.5 | 7.0 |
| *ascF*^1^ | β-glucoside PTS enzyme II | 2,582 | 2,65E-36 | 1040.7 | 173.7 |
| *ascB*^1^ | cryptic 6-P-β-glucosidase | 2,470 | 3,77E-40 | 1203.6 | 217.1 |

**ydfR* is also upregulated in the *yegJ* (-IPTG) RNA-seq analysis with log2 fold change = 1,244 (p-value adj. = 0,06); ^1^genes that are likely co-transcribed together (operon) are indicated by same superscript numbers.

**Suppl. Table S8**. Genes that are differentially regulated upon overexpression of *thrB* as compared to empty vector (S4197 pBGG237) and are not similarly regulated by *slyX*, *ydfR* or *yegJ* overexpression. Genes are listed that are regulated in the RNA-seq analysis of the ThrB overproducer with log2 fold change ≥ 1.5 or ≤ -1.5 (adjusted p-value ≤ 0.05). From the latter list, all genes were eliminated that are also regulated by at least one of the other overproducers (SlyX, YdfR or YegJ) in the same direction (same algebraic sign) with a log2 fold change ≥ 0.5 or ≤ -0.5 (adjusted p-value ≤ 0.1), respectively.

| **Gene name** | **Function** | **log2 fold change** | **adjusted**  **p-value** | **mean normalized reads in overproducer** | **mean normalized reads in S4197 pBGG237** |
| --- | --- | --- | --- | --- | --- |
| *thrB* | homoserine kinase | 12,251 | 0 | 4092484.8 | 839.1 |
| *ytcA* | putative lipoprotein | 4,355 | 0,0484 | 3.8 | 0.0 |
| *ydiN* | putative transporter | 4,006 | 0,00492 | 16.0 | 1.0 |
| *ybeR* | uncharacterized | 2,626 | 0,0143 | 21.9 | 3.6 |
| *insP* | pseudogene | 2,043 | 0,0467 | 14.4 | 3.5 |
| *ydhY* | ferredoxin-like protein | 2,011 | 0,0411 | 22.6 | 5.7 |
| *csgE*^1^ | curli assembly component | 1,924 | 0,00316 | 32.3 | 8.5 |
| *fimC*^2^ | type 1 fimbriae chaperone | 1,897 | 0,000284 | 273.6 | 73.5 |
| *ycjU* | β-phosphoglucomutase | 1,876 | 0,0158 | 21.9 | 6.0 |
| *ybeU* | uncharacterized | 1,755 | 0,00888 | 42.4 | 12.6 |
| *sfmZ* | LuxR family regulator | 1,752 | 0,0237 | 22.0 | 6.6 |
| *ydfX* | Qin prophage protein | 1,658 | 0,0293 | 21.9 | 6.9 |
| *gfcB* | lipoprotein | 1,646 | 0,00042 | 104.1 | 33.4 |
| *tmpR* | Rac prophage protein | 1,639 | 0,0000288 | 125.4 | 40.2 |
| *ompG* | outer membrane protein | 1,615 | 0,00105 | 57.9 | 18.9 |
| *yjfJ* | PspA family protein | 1,599 | 0,000247 | 73.0 | 24.2 |
| *mcbR* | transcriptional regulator | 1,571 | 0,00000185 | 159.2 | 53.7 |
| *phoE* | outer membrane porin | 1,559 | 0,000254 | 102.1 | 34.8 |
| *fimI*^2^ | putative fimbrial protein | 1,558 | 0,00173 | 407.7 | 138.5 |
| *ypdE* | aminopeptidase | 1,555 | 0,04 | 22.0 | 7.5 |
| *fimG*^2^ | type 1 fimbriae subunit | 1,548 | 0,00709 | 57.0 | 19.6 |
| *ivbL* | *ilvBN* leader peptide | 1,529 | 0,0045 | 2764.0 | 957.6 |
| *ydbD* | uncharacterized | 1,521 | 0,000000168 | 209.9 | 73.0 |
| *csgD*^1^ | transcriptional regulator | 1,51 | 0,000018 | 154.3 | 54.1 |
| *yehA* | putative fimbrial adhesin | 1,524 | 0,0171 | 27.3 | 9.5 |
| *nrdA*^3^ | ribonucleoside-PP reduct. | -1,505 | 0,00000000000428 | 3611.0 | 10247.9 |
| *yihL* | transcriptional regulator | -1,532 | 0,0000128 | 79.7 | 229.9 |
| *rbsR*^4^ | transcription factor *rbs* | -1,550 | 0,000000277 | 311.6 | 912.2 |
| *ymgG* | uncharacterized | -1,587 | 0,0000159 | 27.8 | 83.6 |
| *rbsC*^4^ | ribose ABC transporter | -1,663 | 0,0000000132 | 556.8 | 1762.3 |
| *yhdV* | lipoprotein | -1,669 | 0,00056 | 21.5 | 68.5 |
| *yfaE*^3^ | [2Fe-2S] cluster protein | -1,762 | 9,77E-19 | 329.9 | 1119.2 |
| *pfo* | putative pyruvate-flavodoxin oxidoreductase | -1,971 | 2,34E-22 | 691.2 | 2709.3 |
| *rbsA*^4^ | ribose ABC transporter | -1,982 | 0,0000195 | 694.2 | 2742.5 |
| *yiaD* | lipoprotein | -2,048 | 2,73E-18 | 448.7 | 1854.9 |
| *ptsG* | glucose transporter | -2,211 | 1,11E-26 | 3272.1 | 15149.2 |
| *cpxP* | Cpx regulator | -2,851 | 0,00000000000000012 | 210.4 | 1518.2 |

**thrB* is also upregulated in the *yegJ* (+IPTG) dataset with log2 fold change = 0,609 (p-value adj. = 0,0917); ^1^genes that are likely co-transcribed together (operon) are indicated by same superscript numbers.

**Suppl. Table S9**. Genes that are differentially regulated upon overexpression of *yegJ* (-IPTG data set) as compared to empty vector (S4197 pBGG237) and are not similarly regulated by *slyX*, *ydfR* or *thrB* overexpression. Genes are listed that are regulated in the RNA-seq analysis of the YegJ overproducer (-IPTG) with log2 fold change ≥ 1.5 or ≤ -1.5 (adjusted p-value ≤ 0.05). From the latter list, all genes were eliminated that are also regulated by at least one of the other overproducers (SlyX, YdfR or ThrB) in the same direction (same algebraic sign) with a log2 fold change ≥ 0.5 or ≤ -0.5 (adjusted p-value ≤ 0.1), respectively. For comparison, the log2 fold changes observed in the YegJ (+IPTG) data set are also listed.

| **Gene name** | **Function** | **log2 fold change**  **yegJ -IPTG** | **log2 fold change**  **yegJ +IPTG** | **adjusted**  **p-value (*yegJ* -IPTG)** | **mean normalized reads in overproducer** | **mean normalized reads in S4197 pBGG237** |
| --- | --- | --- | --- | --- | --- | --- |
| *yegJ* | uncharacterized | 9,311 | 14,178 | 1,57E-91 | 31056,2 | 48,7 |
| *uhpT* | hexose-P transporter | 4,766 | 4,348 | 7,76E-36 | 2447 | 90 |
| *ndh* | NADH:quinone oxidoreductase II | 3,573 | 3,115 | 1,14E-20 | 9620,2 | 808,7 |
| *yegD* | HSP70 family protein | 3,017 | 1,777 | 0,0000000000197 | 1333 | 164,9 |
| *satP* | acetate:H^+^ symporter | 2,530 | 1,478 | 0,0000000000537 | 2238,3 | 387,9 |
| *yqaE***^†^** | predicted membrane protein | 2,508 | 2,616 | 0,00000000000000409 | 522,3 | 92,2 |
| *yjdQ* | pseudogene | 2,143 | 0,986^★^ | 0,00668 | 60,9 | 13,9 |
| *pdhR* | Transcriptional regulator | 1,871 | 1,053 | 0,0000528 | 10837 | 2963,9 |
| *yecR* | uncharacterized lipoprotein | 1,836 | 1,127^★^ | 0,00499 | 32,3 | 9 |
| *purP* | adenine:H^+^ symporter | 1,782 | 1,084 | 0,000000000012 | 2901,8 | 843,9 |
| *yfhR* | putative peptidase | 1,778 | 0,623^★^ | 0,00223 | 189,3 | 55,5 |
| *yafT* | uncharacterized lipoprotein | 1,693 | 0,597^★^ | 0,0000277 | 399,1 | 123,7 |
| *dusB*^1^ | tRNA-dihydrouridine synthase | 1,690 | 0,807 | 0,00000241 | 25242,6 | 7825,1 |
| *ackA* | acetate kinase | 1,674 | 1,364 | 0,00000000000000156 | 29259 | 9172,2 |
| *cydX* | cytochrome bd-I access. SU | 1,616 | 1,235 | 0,0000000333 | 1446,3 | 471,5 |
| *nirC* | nitrite transporter | 1,580 | 1,253 | 0,00000218 | 168,5 | 56,2 |
| *fis*^1^ | transcriptional dual regulator | 1,569 | 0,310^★^ | 0,0000000049 | 21378,7 | 7207,8 |
| *mntP* | Mn^2+^ exporter | 1,540 | 0,657^★^ | 0,000449 | 492,5 | 169,7 |
| *intR* | Rac prophage protein | 1,536 | 0,673^★^ | 0,000951 | 503 | 173,6 |
| *yjjY* | uncharacterized | 1,533 | -0,202^★^ | 0,0433 | 36,2 | 12,6 |
| *yicT* | pseudogene | 1,525 | 1,073^★^ | 0,0159 | 77,9 | 27,2 |
| *brnQ* | branched chain amino acid transporter | 1,516 | 0,667 | 0,000000000047 | 4435 | 1551,4 |
| *lpxT* | lipid A core phosphotransf. | 1,505 | 0,673 | 0,0000000128 | 3080,3 | 1085,6 |
| *sgcX* | putative endoglucanase | -1,510 | -1,337 | 0,0144 | 15,3 | 43 |
| *ycjS*^2^ | D-glucoside dehydrogenase | -1,542 | -0,690^★^ | 0,0000923 | 25,3 | 73,4 |
| *allS* | transcriptional activator | -1,559 | -1,477 | 0,0216 | 15,3 | 45,3 |
| *ycjP^2^* | putative ABC transporter | -1,560 | -0,548^★^ | 0,00551 | 13,8 | 40,8 |
| *xdhC*^3^ | putative xanthine dihydrogen. | -1,572 | -1,234 | 0,00153 | 25,9 | 76,6 |
| *lsrB*^4^ | AI-2 ABC transporter | -1,582 | -1,546 | 0,00605 | 17,4 | 52,3 |
| *allD* | Ureidoglycolate dehydrog. | -1,661 | -1,581 | 0,0292 | 9,6 | 30,3 |
| *ybgD* | putative fimbrial protein | -1,673 | -1,368^★^ | 0,0409 | 6 | 18,9 |
| *araD* | L-ribulose-5-P 4-epimerase | -1,722 | -0,674^★^ | 0,0136 | 7 | 23 |
| *lsrK*^5^ | autoinducer-2 kinase | -1,723 | -0,947 | 0,000000000296 | 78 | 257,3 |
| *yqcE* | putative transport protein | -1,738 | -2,486 | 0,00754 | 11,3 | 37,5 |
| *aceA* | isocitrate lyase | -1,743 | -1,327 | 0,0000000000587 | 1800,1 | 6025 |
| *ydjK* | putative transporter | -1,744 | -0,975 | 0,00163 | 12,7 | 42,8 |
| *preA*^6^ | dihydropyrimidine dehydrog. | -1,765 | -1,548 | 0,0000000847 | 61,2 | 207,6 |
| *gatR*^7^ | transcriptional repressor | -1,766 | -1,917 | 0,0000000249 | 257,6 | 875,9 |
| *cytR* | transcriptional repressor | -1,789 | -0,787 | 0,00000000208 | 1425,7 | 4925,5 |
| *frlC* | fructoselysine 3-epimerase | -1,790 | -0,900 | 0,0000173 | 22 | 75 |
| *paaG*^8^ | phenylacetate-CoA isomer. | -1,833 | -1,170^★^ | 0,00765 | 6,6 | 23,1 |
| *ycjT*^2^ | kojibiose phosphorylase | -1,841 | -0,254^★^ | 0,00000143 | 22,1 | 80,2 |
| *ygcO* | putative 4Fe-4S cluster prot. | -1,887 | -1,713^★^ | 0,0279 | 7,4 | 27,4 |
| *ycjQ*^2^ | D-guloside 3-dehydrogenase | -1,893 | -1,079 | 0,00000106 | 21,6 | 81 |
| *ygeW* | putative carbamoyltransf. | -1,897 | -1,592 | 0,00623 | 6,4 | 23,9 |
| *bglG* | antiterminator | -1,913 | -1,767 | 0,0000935 | 25,2 | 94,9 |
| *xdhB*^3^ | putative xanthine dihydrogen. | -1,962 | -1,597 | 0,000115 | 26,5 | 102,7 |
| *ydcJ* | uncharacterized protein | -1,967 | -1,603 | 0,000062 | 39,2 | 153,4 |
| *ycjR*^2^ | 3-dehydro-D-guloside 4-epim. | -1,974 | -0,681^★^ | 0,00000212 | 18,1 | 69,8 |
| *ycgR* | flagellar brake protein | -1,977 | -0,397^★^ | 0,0304 | 3,8 | 14,8 |
| *ftnB* | putative ferritin-like protein | -2,018 | 1,037 | 0,0000000000000102 | 249,2 | 1008,6 |
| *rspA*^9^ | dehydratase | -2,044 | -1,639 | 0,00000000296 | 22 | 90,5 |
| *rhmD* | L-rhamnonate dehydratase | -2,058 | -1,509 | 0,00278 | 7,2 | 29,2 |
| *lsrD*^4^ | AI-2 ABC transporter | -2,060 | -1,050^★^ | 0,00225 | 6,6 | 27 |
| *yniA* | putative kinase | -2,079 | -1,822 | 0,00000437 | 142,4 | 601,6 |
| *paaF*^8^ | 2,3-dehydroadipyl-CoA hydrat. | -2,130 | -1,329 | 0,000106 | 9,1 | 39,9 |
| *rspB*^9^ | putative dehydrogenase | -2,140 | -1,348 | 0,0000133 | 10,3 | 45,1 |
| *lsrC*^4^ | AI-2 ABC transporter | -2,164 | -1,135 | 0,00263 | 9 | 41,8 |
| *yjiL* | putative ATPase | -2,284 | -1,066 | 0,000361 | 13,5 | 65,2 |
| *galP***^†^** | galactose:H^+^ symporter | -2,331 | -2,537 | 0,000000000853 | 450,8 | 2267,2 |
| *ygcW* | putative deoxygluconate dehydrog, | -2,348 | -1,236^★^ | 0,0289 | 2,5 | 13,3 |
| *paaE*^8^ | phenylacetate-CoA oxyg. | -2,362 | -1,718 | 0,0000000297 | 15,6 | 79,9 |
| *preT*^6^ | dihydropyrimidine dehydrog. | -2,578 | -2,111 | 0,00000000023 | 64,9 | 386,5 |
| *uidB* | glucuronide:H^+^ symporter | -2,585 | -1,459 | 0,0000000255 | 21,4 | 128,4 |
| *gcvP*^10^ | glycine cleavage system | -2,604 | -0,926 | 0,000000000000217 | 1009,7 | 6138,5 |
| *lsrR*^5^ | transcriptional repressor | -2,687 | -1,066 | 0,000000000011 | 39 | 249,9 |
| *ycjO*^2^ | putative ABC transporter | -2,710 | -1,235 | 0,00000368 | 7,1 | 45,7 |
| *ybdD* | uncharacterized protein | -2,801 | -2,071 | 0,00000052 | 43,9 | 303,6 |
| *gcvH*^10^ | glycine cleavage system | -2,968 | -1,588 | 0,0000000000000000921 | 517,9 | 4049,3 |
| *gcvT*^10^ | glycine cleavage system | -3,194 | -1,557 | 0,00000000000000351 | 686,9 | 6286,1 |
| *cmtB* | EIIA domain | -3,202 | -1,469 | 0,0000606 | 3,3 | 29,7 |
| *dadX* | alanine racemase 2 | -3,205 | -2,116 | 2,18E-18 | 591,2 | 5449,3 |
| *ddpX* | D-alanyl-D-alanine dipeptid. | -3,210 | -1,445^★^ | 0,00378 | 2,6 | 24,3 |
| *paaD*^8^ | phenylacetate-CoA oxygen. | -3,288 | -2,525 | 0,0000179 | 3,5 | 36,1 |
| *eutS* | microcompartment shell prot. | -3,397 | -2,308^★^ | 0,0261 | 1,1 | 11,2 |
| *ykgR* | uncharact. membr. prot. | -3,590 | -3,040 | 0,0000547 | 5,5 | 65,3 |
| *phoH* | ATP-binding protein | -3,652 | -3,096 | 0,000164 | 88,5 | 1111,7 |
| *lacA* | galactoside O-acetyltransf. | -3,779 | 0,839^★^ | 9,78E-19 | 38,1 | 522,5 |
| *paaC*^8^ | phenylacetate-CoA oxygen. | -4,254 | -2,314 | 0,000000231 | 2,7 | 51,1 |
| *gatD*^7^ | L-galactitol-1-P 5-dehydrogen. | -4,912 | -3,193 | 9,86E-66 | 177,4 | 5341,1 |

**^†^**regulated by CpxR~P; ^★^p-value > 0,05 = not significant; ^1^genes present in the same transcription unit (operon) are indicated by same superscript numbers. Grey shaded boxes indicate a similar regulation in the YegJ (+IPTG) RNA-seq analysis (log2 fold change ≥ 1.0 or ≤ -1.0; adjusted p-value ≤ 0.05).

**Suppl. Table S10**. Genes that are differentially regulated upon overexpression of *yegJ* (+IPTG data set) as compared to empty vector (S4197 pBGG237) and are not similarly regulated by *slyX*, *ydfR* or *thrB* overexpression. Genes are listed that are regulated in the RNA-seq analysis of the YegJ overproducer (+IPTG) with log2 fold change ≥ 1.5 or ≤ -1.5 (adjusted p-value ≤ 0.05). From the latter list, all genes were eliminated that are also regulated by at least one other overproducer (SlyX, YdfR or ThrB) in the same direction (same algebraic sign) with a log2 fold change ≥ 0.5 or ≤ -0.5 (adjusted p-value ≤ 0.1), respectively. For comparison, the log2 fold changes observed in the YegJ (-IPTG) data set are also listed.

| **Gene name** | **Function** | **log2 fold change**  **yegJ +IPTG** | **log2 fold change**  **yegJ -IPTG** | **adjusted**  **p-value (*yegJ* +IPTG)** | **mean normalized reads in overproducer** | **mean normalized reads in S4197 pBGG237** |
| --- | --- | --- | --- | --- | --- | --- |
| *yegJ* | uncharacterized | 14,178 | 9,311 | 2,83E-213 | 906097,6 | 48,7 |
| *yncJ* | uncharacterized | 4,861 | 0,188^★^ | 2,32E-42 | 464,5 | 16 |
| *uhpT* | hexose-P transporter | 4,348 | 4,766 | 1,81E-29 | 1831,8 | 90 |
| *degP***^†^** | periplasmic serine protease | 4,059 | -0,616 | 2,96E-68 | 69999,2 | 4198,2 |
| *yebE***^†^** | inner membrane protein | 3,919 | 0,069^★^ | 1,2E-80 | 2250,5 | 148,8 |
| *cpxP***^†^** | periplasmic stress protein | 3,851 | 0,326^★^ | 8,08E-33 | 21900,9 | 1518,2 |
| *ldtC***^†^** | L,D-transpeptidase | 3,417 | -0,049^★^ | 1,69E-46 | 2335 | 218,8 |
| *ndh* | NADH:quinone oxidoreduct. II | 3,115 | 3,573 | 0,00000000000000117 | 7005,9 | 808,7 |
| *alx* | Mn^2+^ exporter | 2,711 | 1,168 | 0,000000000000117 | 2451,1 | 374,7 |
| *nhaA*^1^ | Na^+^:H^+^ antiporter | 2,691 | 1,263 | 1,78E-24 | 8228,9 | 1274,9 |
| *yqaE***^†^** | predicted membrane protein | 2,616 | 2,508 | 0,000000000000000376 | 563 | 92,2 |
| *ymgG*^2^ | uncharacterized | 2,569 | 0,535^★^ | 1,15E-20 | 496,6 | 83,6 |
| *chaA***^†^** | Na^+^/K^+^:proton antiporter | 2,522 | 0,547 | 6,57E-39 | 8642,5 | 1505,1 |
| *ymgD*^2^ | uncharacterized | 2,105 | 0,467^★^ | 0,000000000000000352 | 757,5 | 176,2 |
| *spy***^†^** | periplasmic chaperone | 1,920 | -0,756 | 3,02E-19 | 1076,3 | 284,4 |
| *hycD* | hydrogenase subunit | 1,919 | 0,489^★^ | 0,00397 | 32 | 8,4 |
| *secA* | translocation ATPase | 1,913 | 0,372^★^ | 1,92E-27 | 48077,4 | 12767,7 |
| *pspB*^3^ | phage shock protein | 1,912 | -0,077^★^ | 0,00000133 | 220,2 | 58,4 |
| *pspC*^3^ | phage shock protein | 1,884 | -0,223^★^ | 0,000000000689 | 345,8 | 93,6 |
| *yegD* | HSP70 family protein | 1,777 | 3,017 | 0,000217 | 564,5 | 164,9 |
| *yjbG* | capsule biosynthesis | 1,766 | 0,678^★^ | 0,0455 | 17,1 | 5,1 |
| *sbmA***^†^** | peptide antibiotic transporter | 1,737 | 0,133^★^ | 0,0000000000000866 | 1088,9 | 326,8 |
| *mdtI* | multidrug efflux pump | 1,667 | -1,136 | 0,00000145 | 472 | 148,5 |
| *nhaR*^1^ | transcriptional activator | 1,644 | 0,529 | 0,000000000000765 | 1809,7 | 579 |
| *rbsA* | ribose ABC transporter | 1,590 | 0,148^★^ | 0,000713 | 8254,7 | 2742,5 |
| *acrD***^†^** | multidrug efflux pump | 1,554 | 0,413^★^ | 0,000000000121 | 910,2 | 310,4 |
| *pspG* | phage shock protein | 1,544 | 0,803^★^ | 0,0000342 | 208 | 71,6 |
| *rhmD* | L-rhamnonate dehydratase | -1,509 | -2,058 | 0,0273 | 10,3 | 29,2 |
| *yiaO* | periplasmic binding protein | -1,523 | -0,838^★^ | 0,0412 | 9 | 26,1 |
| *lsrB* | AI-2 ABC transporter | -1,546 | -1,582 | 0,00869 | 17,7 | 52,3 |
| *hiuH* | hydroxyisourate hydrolase | -1,547 | -0,771 | 0,0000247 | 33,2 | 96,7 |
| *preA*^4^ | dihydropyrimidine dehydrog. | -1,548 | -1,765 | 0,0000033 | 71 | 207,6 |
| *yahO* | uncharacterized | -1,553 | -1,071 | 0,000000656 | 88,8 | 261,3 |
| *gcvT*^5^ | glycine cleavage system | -1,557 | -3,194 | 0,000367 | 2135,3 | 6286,1 |
| *allD* | Ureidoglycolate dehydrog. | -1,581 | -1,661 | 0,0453 | 10,1 | 30,3 |
| *gcvH*^5^ | glycine cleavage system | -1,588 | -2,968 | 0,000026 | 1346,8 | 4049,3 |
| *ygeW* | putative carbamoyltransf. | -1,592 | -1,897 | 0,0227 | 7,8 | 23,9 |
| *xdhB* | putative xanthine dehydrog. | -1,597 | -1,962 | 0,00206 | 33,7 | 102,7 |
| *ydcJ* | uncharacterized protein | -1,603 | -1,967 | 0,00142 | 50,4 | 153,4 |
| *paaE*^6^ | phenylacetate-CoA oxygen. | -1,718 | -2,362 | 0,000024 | 24,1 | 79,9 |
| *bglG* | antiterminator | -1,767 | -1,913 | 0,00035 | 27,5 | 94,9 |
| *yeiL* | transcriptional activator | -1,819 | -1,147 | 0,000123 | 18,7 | 65,9 |
| *yniA* | putative kinase | -1,822 | -2,079 | 0,0000759 | 170,1 | 601,6 |
| *gatR*^7^ | transcriptional repressor | -1,917 | -1,766 | 0,00000000155 | 232 | 875,9 |
| *ybdD* | uncharacterized protein | -2,071 | -2,801 | 0,000285 | 72,2 | 303,6 |
| *preT*^4^ | dihydropyrimidine dehydrog. | -2,111 | -2,578 | 0,000000268 | 89,5 | 386,5 |
| *dadX* | alanine racemase 2 | -2,116 | -3,205 | 0,0000000223 | 1256,3 | 5449,3 |
| *paaC*^6^ | phenylacetate-CoA oxygen. | -2,314 | -4,254 | 0,0000849 | 10,4 | 51,1 |
| *yqcE* | putative transport protein | -2,486 | -1,738 | 0,000417 | 6,5 | 37,5 |
| *paaD*^6^ | phenylacetate-CoA oxygen. | -2,525 | -3,288 | 0,000228 | 6,4 | 36,1 |
| *galP***^†^** | galactose:H^+^ symporter | -2,537 | -2,331 | 0,000000000026 | 390,5 | 2267,2 |
| *cadB*^8^ | lysine:cadaverine antiporter | -2,921 | -0,508^★^ | 0,00000024 | 41 | 310,5 |
| *cadA*^8^ | lysine decarboxylase | -2,954 | -0,471^★^ | 0,000513 | 82,3 | 638,5 |
| *ykgR* | uncharacterized membr. prot. | -3,040 | -3,590 | 0,000458 | 7,9 | 65,3 |
| *phoH* | ATP-binding protein | -3,096 | -3,652 | 0,00196 | 129,9 | 1111,7 |
| *gatD*^7^ | L-galactitol-1-P 5-dehydrog. | -3,193 | -4,912 | 2,43E-29 | 583,9 | 5341,1 |

**^†^** regulated by CpxR~P; ^★^p-value > 0,05 = not significant. ^1^genes that are likely co-transcribed together (operon) are indicated by same superscript numbers. Grey shaded boxes indicate a similar regulation in the YegJ (-IPTG) RNA-seq analysis (log2 fold change ≥ 1.0 or ≤ -1.0; adjusted p-value ≤ 0.05).

**SUPPLEMENTARY RESULTS**

**Sequences and features of non-annotated peptides/proteins fused to T25, identified in the BACTH screen for Rne interaction partners.**

Protein features i.e. pI, aliphatic index and grand average of hydropathicity (GRAVY) were deduced by using <https://web.expasy.org/cgi-bin/protparam/protparam>. The aliphatic index of a protein is defined as the relative volume occupied by aliphatic side chains (alanine, valine, isoleucine, and leucine). It may be regarded as a positive factor for the increase of thermostability of globular proteins. The GRAVY value for a peptide or protein is calculated as the sum of [hydropathy values](https://web.expasy.org/protscale/pscale/Hphob.Doolittle.html) of all the amino acids, divided by the number of residues in the sequence. In respect to amino acid composition, the four most abundant amino acids are listed.

Renf-1:

KPLCRCLTLGISCNDWCGVCSLLKLICCRLTLAVARWGR*

Theoretical pI: 9.08; Aliphatic index: 120.00; GRAVY: 0.731

20.5% Leu, 17.9% Cys, 10.3% Arg, 7.7% Gly

This peptide (in bold below) is present within a 64 aa long *orf* encoded on the opposite strand of *yacH* and could potentially start with the underlined Leu (TTG). A suitable Shine-Dalgarno sequence is present 7 nt upstream (gagcgg)

LLLGQWLATAEVVEVVIAPIVFSFI**KPLCRCLTLGISCNDWCGVCSLLKLICCRLTLAVARWGR***

Renf-4:

RITRYALKGRLSKIFSSLSWKTCLDRARHDAGGDVITKRKRTASREKRRYCWSGAITKNIAMILNAII*

Theoretical pI: 11.25; Aliphatic index: 89.12; GRAVY: -0.431

14.7% Arg, 11.8% Ile, 10.3% Ala, 10.3% Lys

In the genome, the *orf* is not extended further upstream. The first two Leu codons are TTG and could potentially serve as start codons.

Rnef-7

IQMSGCHVNSRADLSIVLPRRLCALRRVRHQTIKSFARPGHAEFITRTLFERLLAAIEIVNFGKKLIVSRFKFRGVPK*

Theoretical pI: 11.77; Aliphatic index: 105.00; GRAVY: 0.032

14.1% Arg, 10.3% Leu, 9.0% Ile, 7.7% Ala (Phe, Val)

In the genome the *orf* (in bold below) extends beyond the 3’ end of the insert in rnef-7. The Met at pos. 3 could serve as start codon, potentially encoding a 100 aa long protein.

**IQMSGCHVNSRADLSIVLPRRLCALRRVRHQTIKSFARPGHAEFITRTLFERLLAAIEIVNFGKKLIVSRFKFR**VCSLLCRHIIIDAGIVMYTVLTEPQRIL*

Rnef-18

HNNICERAIRPVVMGRKAWLFAGSLVAGNRAAQIMSLLGTAVWSHMLG*

Theoretical pI: 11.54; Aliphatic index: 103.75; GRAVY: 0.388

14.6% Ala, 10.4% Leu, 10.4% Gly, 8.3% Arg, 8.3% Val

This peptide is part of a longer potential *orf* overlapping with *yjgZ*. However, there is no suitable start codon. Met14 (underlined) within the peptide fused to T25 could serve as start codon.

RneN-78

LYRLLFFIIRKLSNKLY*

Theoretical pI: 10.45; Aliphatic index: 160.59; GRAVY: 0.582

29.4% Leu, 11.8% Arg, 11.8% Ile, 11.8% Lys, 11.8% Phe, 11.8% Tyr

In the genome, there is no significant extension of this short *orf*.

RneN-116

MSSINSLNSALRISLKIANMRYKWLMRVFVRLIIRSY*

Theoretical pI: 11.84; Aliphatic index: 126.49; GRAVY: 0.349

16.2% Ser, 13.5% Arg, 13.5% Ile, 13.5% Leu

In the genome, this *orf* (in bold below) could be extended further upstream:

*AGLFINMTNLLNWLRLSIAC**MSSINSLNSALRISLKIANMRYKWLMRVFVRLIIRSY***

RneN-203

KYFPAVGGKTTIFLRFLCLCAGFFGLYLAAISAYSNL*

Theoretical pI: 9.24; Aliphatic index: 105.68; GRAVY: 1.030

16.2% Leu, 13.5% Ala, 13.5% Phe, 10.8% Gly

Val6 (GTG) could serve as start codon. In the genome, the *orf* does not extend further upstream.

RneM-30

RISVSAIRYISLPASKPRKRSFRHCRIKSALIHKTANSGRYWASTICGKTITAIRCWRTVRRCNYVVRTLNCMRRWRRCFIGYLSN*

Theoretical pI: 11.68; Aliphatic index: 79.42; GRAVY: -0.347

18.6% Arg, 10.5% Ile, 10.5% Ser, 7.0% Ala, 7.0% Cys, 7% Thr.

In the genome, this *orf* does not extend further upstream.

RneM-38

LTSGSCAGNHFRVSFDFMKIFLKILLIFIAILYV*

Theoretical pI: 9.20; Aliphatic index: 137.65; GRAVY: 1.300

14.7% Ile, 14.7% Leu, 14.7% Phe, 8.8% Ser

In the genome, this *orf* does not extend further upstream.

pYG195_D_c38

LVTIATTTLTTYAQVTRRAARTTRHATVVTVISIVLFLLFWCSLRYDWQRDFHQFCAWSWRFLNNWFGSDRGT*

Theoretical pI: 10.69; Aliphatic index: 85.48; GRAVY: 0.110

16.4% Thr, 11% Arg, 9.6% Leu, 8.2% Ala, 8.2% Phe, 8.2% Val

This peptide (in bold below) is encoded within a 551 long *orf* present on the opposite strand of *rne*. The underlined methionine could serve as start codon. A possible Shine-Dalgarno sequence (AGGAGC) is present 9 nt upstream of this methionine.

LRTRRSGRGMMCCRMTTCGTFTFEGKSRALPVTVTRCLRNIFRCWSARHWRGSVVMLNGDLRFDRSYRFNFCCDFSDYWRGRLSNNFRFSNYNLSSFDNFFNICRLFLRFNHRFCLFCNFLGYGNIRLGNHLRLFSYRGSDHFRMRRFNHFRLRFGNLNGGFYDTGHFFNNWRANNRFDSGGDRDLSHHRLHMYFFLFALLFNLYILRTYNWIADPDFARGQFRRRARYGQRHWRLGWIT**LVTIATTTLTTYAQVTRRAARTTRHATVVTVISIVLFLLFWCSLRYDWQRDFHQFCAWSWRFLNNWFGSD**SFFNHRCDYRFFGYALLVTHFLIELTLFTTRLNRTYTFFLFGFLNRLLFNIQRLRFLLRLTFIIITAAATFTTRRLLLVGGTRFFRYLSLLTALTRLGSLLLCLATITTVFFAIIAAFSTFTTGVAALITVTAVVLTRLATILLTFRFRFCFRCWLLGRFGFFTTAEQRFQCAEEAAQQPRLCRSCWCCRCSFRCWSYNRRGRFSWRWRRRHIRHGKGCQRRLFRTLTFSELFFRRQRHRFFMQLRQHVA*

pYG195_D_c48

STSGIFTVATLLTVFALAVSCIWIFVRCLA*

Theoretical pI: 7.80; Aliphatic index: 143.00; GRAVY: 1.837

13.3% Ala, 13.3% Leu, 13.3% Thr, 13.3% Val.

pYG195_D_c53

PRGEQDERTHGNGCHRRYLCRALAVSVFTCRALSRMGIIRNHWLRRLLQFLCRRRR*

Theoretical pI: 11.75; Aliphatic index: 78.39; GRAVY: -0.689

25% Arg, 12.5% Leu, 7.1% Cys, 7.1% Gly

pYG195_D_c72

TGSTSYPLASAQGQITRFRRNRFWLRFWCWFRIRYRFWRRLWVRRWLRRRLRVWCWIRFHFRQRGT*

Theoretical pI: 12.45; Aliphatic index: 59.09; GRAVY: -0.779.

28.8% Arg, 12.1% Trp, 10.6% Phe, 7.6% Leu

pYG195_D_c91

PPGWSRKISAWRRKLHKKRRVVSRLRKSWNGYVKVLKAVSRKVKHVWRALKNGYLSK*

Theoretical pI: 12.31, Aliphatic index: 81.93, GRAVY: -0.946

19.3% Lys, 15.8% Arg, 12.3% Val, 10.5% Ser

57 aa peptide encoded within *ettA*, but in different frame.

**SUPPLEMENTARY REFERENCES**

Benjamini Y, Hochberg Y. 1995. Controlling the False Discovery Rate: A Practical and Powerful Approach to Multiple Testing. *Journal of the Royal Statistical Society Series B* **51**: 289–300.

Golby P, Kelly DJ, Guest JR, Andrews SC. 1998. Topological analysis of DcuA, an anaerobic C4-dicarboxylate transporter of *Escherichia coli*. *J Bacteriol* **180**: 4821-4827.

Karp PD, Paley S, Caspi R, Kothari A, Krummenacker M, Midford PE, Moore LR, Subhraveti P, Gama-Castro S, Tierrafria VH et al. 2025. The EcoCyc database (2025). *EcoSal Plus* **13**: eesp00192024.
